# Supplementary material for: The L27 domain of MPP7 enhances TAZ-YY1 cooperation to renew muscle stem cells
Source: EMBO Rep. 2024 Nov 4;25(12):5667–86. doi: 10.1038/s44319-024-00305-4 (PMC11624273; doi:10.1038/s44319-024-00305-4)
Supplement: Supplementary file 1 — Appendix [file 44319_2024_305_MOESM1_ESM.pdf]

APPENDIX

Content:

- **Appendix Figure S1.** Additional data to support Figure 1……………………Page 2
- **Appendix Figure S2.** Additional data to support Figure 2……………………Page 4
- **Appendix Figure S3.** Additional data to support Figure 3……………………Page 6
- **Appendix Figure S4.** Additional data to support Figure 4……………………Page 8
- **Appendix Figure S5.** Additional data to support Figure 5……………………Page10
- **Appendix Figure S6.** Additional data to support Figure 6……………………Page12
- **Appendix Figure S7.** Additional data to support Figure 7……………………Page 14
- **Appendix Figure S8.** Additional data to support Figure 8……………………Page 16
- **Appendix Figure S9.** Antibody validation and background threshold………Page 17
- **Reference**…………………………………………………………………….Page 18

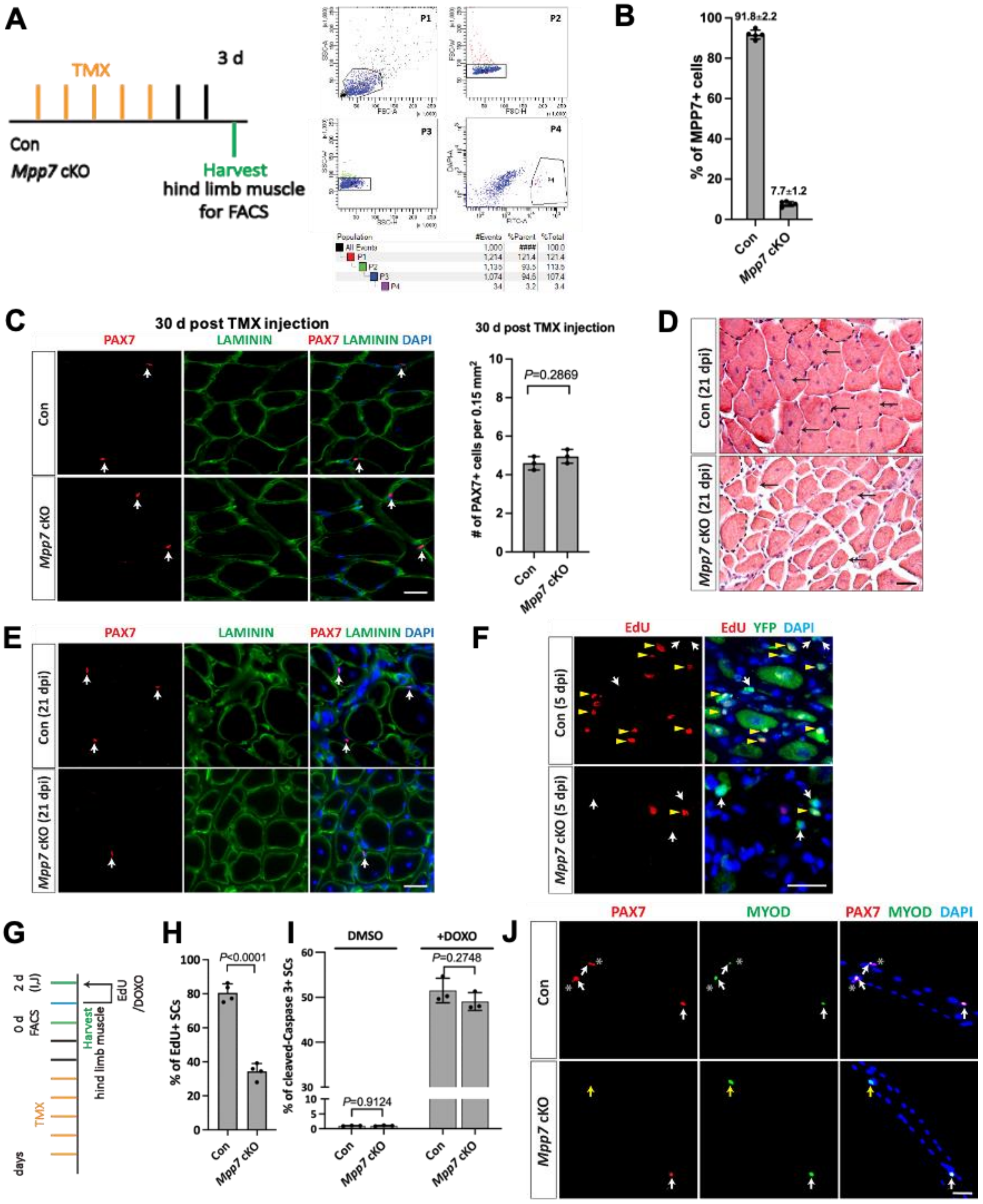

Appendix Figure S1. Additional data to support Fig. 1.

A Scheme for FACS isolation of YFP-marked Con and *Mpp7* cKO MuSCs from hindlimb muscles; FACS profiles to the right. **B** Percentages of MPP7$^+$ MuSCs from Con and *Mpp7* cKO immediately after FACS isolation. **C** Representative IF images of PAX7 and LAMININ for Con and *Mpp7* cKO TA muscles 30 d (days) after TMX regimen without injury; white arrows, PAX7$^+$ MuSCs; quantification to the right. **D, E** Representative images of H&E histology (**D**) and IF (**E**) at 21 dpi. Black arrows indicate regenerated

myofibers, and dashed lines, injury boundary in (**D**); white arrows, $PAX7^+$ cells in (**E**). **F** Representative images of EdU Click-reaction with IF of YFP; yellow arrowheads, EdU^+YFP^+ cells; white arrows, EdU^-YFP^+ cells. **G** Regimen to determine EdU incorporation and programmed cell death of MuSCs in culture. Percentages of EdU^+ (**H**) and cleaved-Caspase 3^+ cells (**I**), respectively, from experiment in (**F**, **G**). DOXO was used to demonstrate cleaved-Caspase 3 reactivity. **J** Representative IF images of PAX7 and MYOD with DAPI from single myofiber cultures; asterisks, $PAX7^+$ cells; white arrows, $PAX7^+MYOD^+$ cells; yellow arrows, $MYOD^+$ cells.
Data information: Scale bars = 25 µm in (**C-F, J**). (**B**) N = 5 mice, each. (**C, I**) N=3 mice, each. (**H**) N=4 mice, each. Error bars represent means ± SD. Student's *t*-test (two-sided).

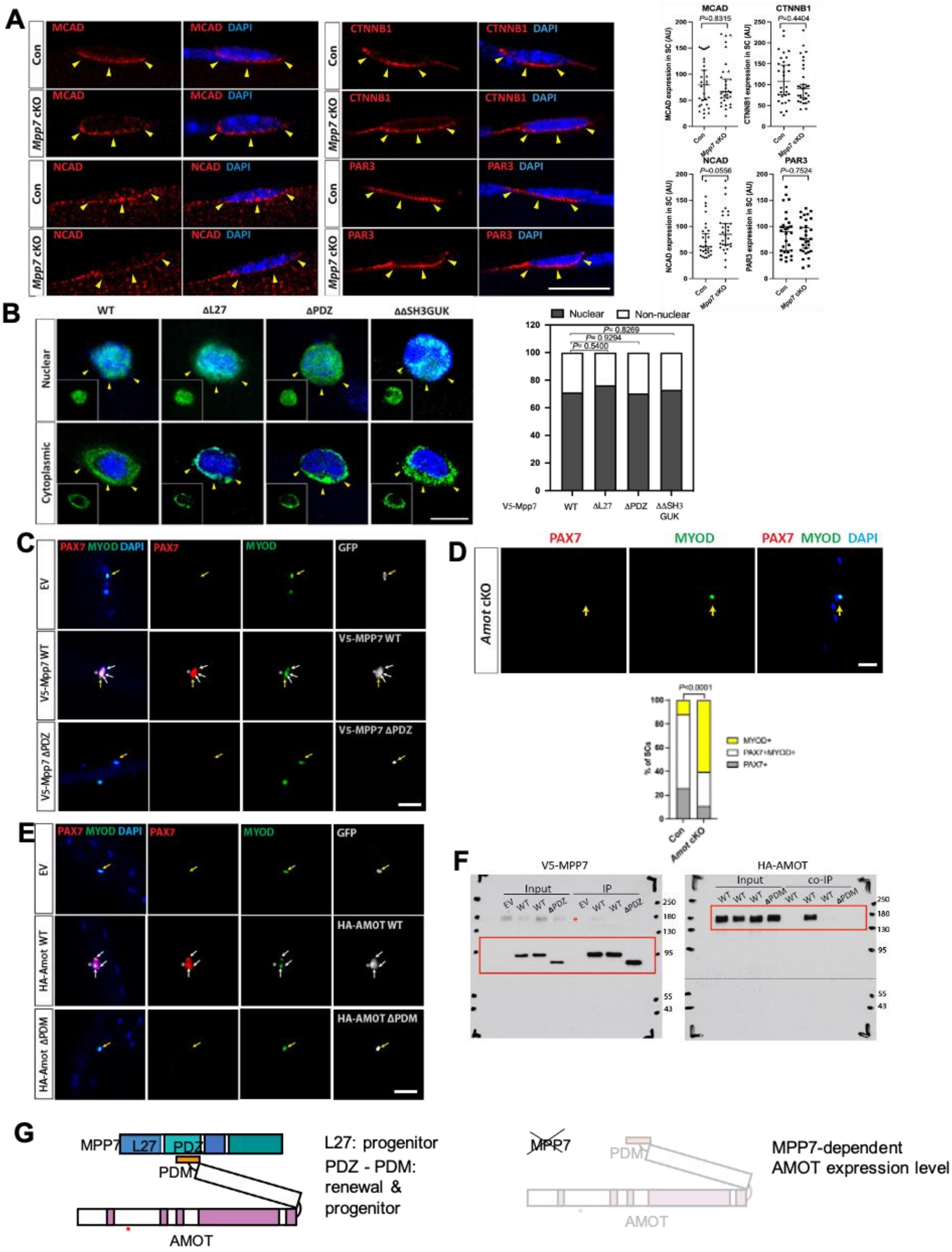

Appendix Figure S2: Additional data to support Fig. 2.

A IF of M-Cadherin (MCAD), N-Cadherin (NCAD), β-Catenin (CTNNB1), and PAR3 in Con (*Pax7*$^{CreERT2/+}$) and *Mpp7* cKO MuSCs immediately after single myofiber isolation; yellow arrowheads, apical side. Quantified fluorescent signals (AU) are to the right. **B**

Distribution pattern of V5-MPP7 variants in transfected *Mpp7* cKO MuSCs on SM was determined by IF of V5; yellow arrowheads, apical side. Relative fractions of nuclear versus non-nuclear MPP7 are to the right. **C** Representative IF images of SM complementation assay using empty vector (with IRES-mGFP), V5-tagged Mpp7 WT, and V5-tagged Mpp7 △PDZ transfected into *Mpp7* cKO MuSCs to determine cell fates, by co-staining for PAX7, MYOD, and counter stained with DAPI. **D** Representative IF images of *Amot* cKO SM culture stained for PAX7 and MYOD, counted stained with DAPI; quantification to the bottom; Con from Figure 1**J**. **E** Same assay as in (**c**), except for the use of empty vector, HA-Amot WT and HA-Amot △PDM for transfection into *Amot* cKO MuSCs. **F** Original western blots for Figure 2D; red rectangles outline the cropped images used; asterisks, non-specific bands. Boxed areas were cropped and used. **G** Summarized models: Top, MPP7 and AMOT interaction domains. Same color-coded domains of each protein as in Figure 2D, with only relevant domains labeled; * denotes S175 of AMOT. Bottom: AMOT level depends on MPP7.
Data information: Scale bars = 10 µm in (**A, B**), and 25 µm in (**C, D, E**). (**A**) 30 cells in each group. N = 2 Con mice; N = 3 *Mpp7* cKO mice. (**B**) WT, 52 cells; △L27, 55 cells; △PDZ, 54 cells; △△SH3GUK, 52 cells. Bars represent medians ± 95% CI (**A**). Mann–Whitney test in (**A**); Chi-square test in (**B, D**).

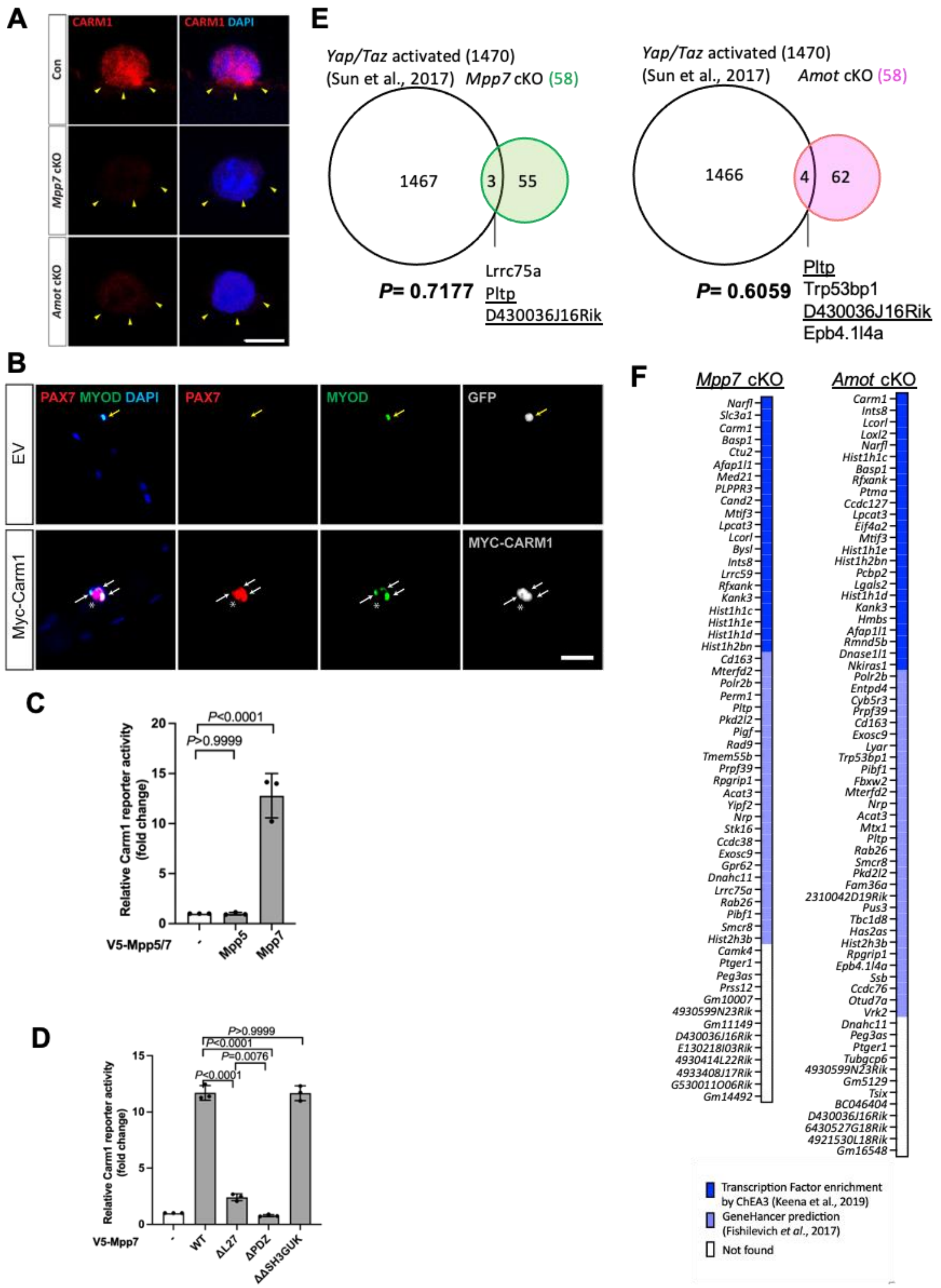

Appendix Figure S3. Additional data to support Figure 3.
A Representative IF images of CARM1 in *Mpp7* cKO and *Amot* cKO MuSCs in SM culture at 48 h; yellow arrowheads, apical side. **B** Representative IF images of *Mpp7* cKO MuSCs in SM culture transfected with either empty or Myc-Carm1 expressing vector (both with IRES-mGFP for identification by GFP), and co-IF for PAX7 and MYOD, with DAPI counter stain. **C** Relative activities of the Carm1-reporter with co-transfected with empty (-) or V5-Mpp7 expression constructs in 293T cells. **D** Relative

activities of the Carm1-reporter when co-transfected with WT and deletion mutants of Mpp7 expression constructs (x-axis) in 293T cells; (-), empty expression construct. **E** Venn Diagrams compare DEGs from myoblasts transduced by stabilized forms of YAP (S127A) and TAZ (S89A) (Sun *et al*, 2017) versus DEGs in *Mpp7* cKO (left) and *Amot* cKO (right) MuSCs. **F** TEAD binding site analysis of DEGs of the *Mpp7* cKO and the *Amot* cKO based on integrated experimental data sets (Keenan *et al*, 2019) and consensus sequence prediction (Fishilevich *et al*, 2017).
Data information: Scale bar = 10 µm in (**A**) and 25 µm in (**B**). (**C, D**) n=3 biological replicates. Error bars represent means ± SD. One-way ANOVA with Tukey's post hoc test was performed in (**C, D**). Hypergeometric test was performed in (**E**).

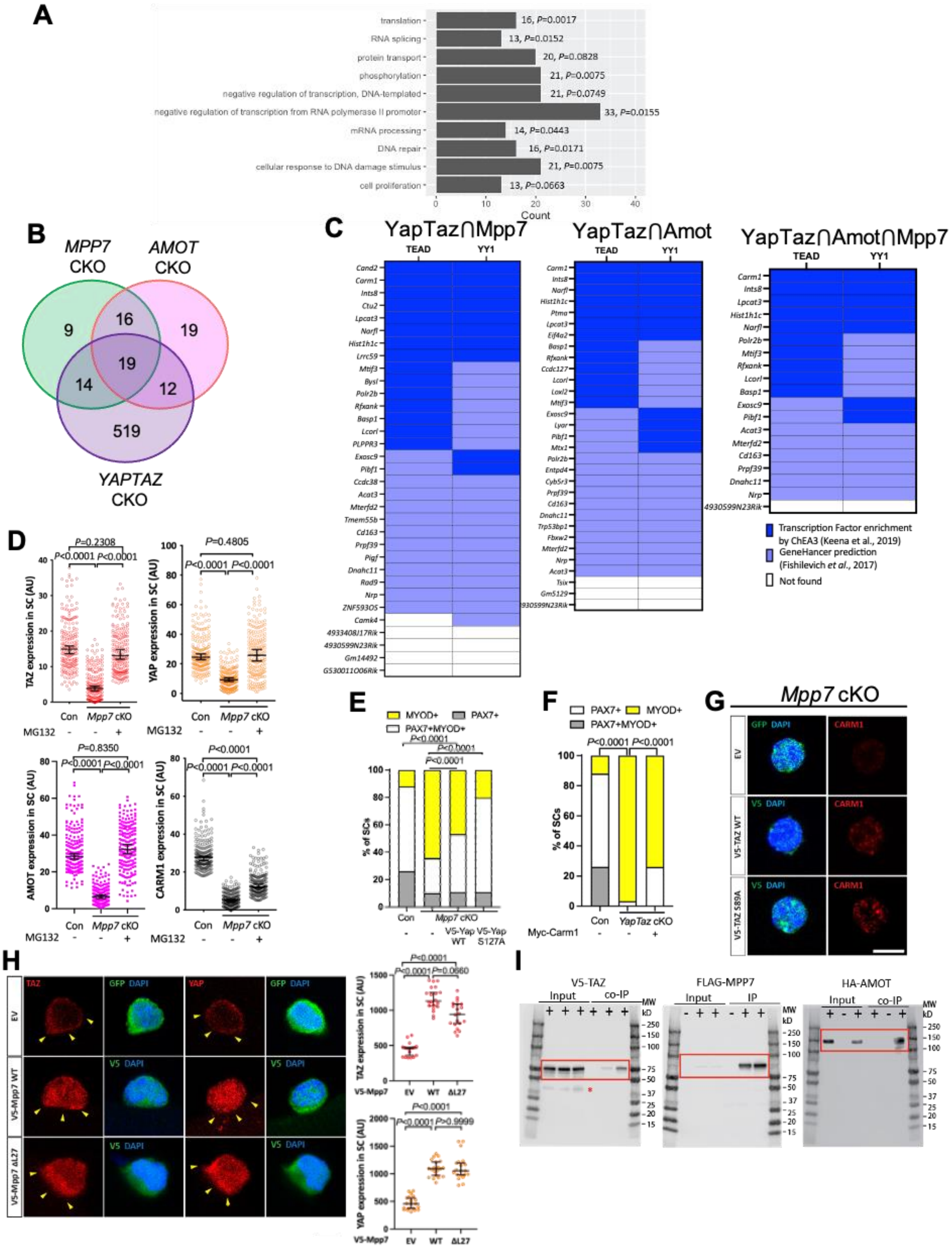

Appendix Figure S4: Additional data to support Fig. 4.

A Top 10 GO-term enriched pathways ranked by gene counts for the *YapTaz* cKO; gene counts and *P*-values on the right. **B** Venn diagram for overlapping DEGs in the *Mpp7* cKO, the *Amot* cKO, and the *YapTaz* cKO. **C** Enrichment of TEAD and YY1

binding sites in promoter regions of DEGs common between the *YapTaz* cKO and the *Mpp7* cKO, between the *YapTaz* cKO and the *Amot* cKO, and among all 3 cKOs; sources for analyses at the bottom. **D** Quantification of IF signals (AU) of TAZ, YAP, AMOT, and CARM1 of 48 h cultured Con (*Pax7*$^{CreERT2/+}$), *Mpp7* cKO, and *Mpp7* cKO MuSCs treated with MG132 (added 24 h prior to assay); Con (-), *Mpp7* cKO (-) from Figure 4C (TAZ), Figure 4D (YAP), Figure 2K (AMOT) and Figure 3G (CARM1). **E** Quantification of cell fate fractions in SM culture of control (Con), *Mpp7* cKO transfected with WT Yap or S127A Yap expression plasmids; Con (-) and *Mpp7* cKO from Figure 2C. **F** Quantification of cell fate fractions in SM culture of control (Con), *YapTaz* cKO transfected with Carm1 expression plasmid; Con (-) from Figure 2C. **G** Representative IF images of GFP, V5 and CARM1 of 48 h cultured *Mpp7* cKO cells after transfected with empty (with IRES-mGFP), V5-Taz and V5-Taz S89A expression vectors. **H** Representative images (left panels) and quantification (right 2 charts) of TAZ and YAP levels in *Mpp7* cKO cells in SM culture transfected by empty (Con), WT Mpp7, and ∆L27 Mpp7 vectors (all with IRES-GFP); yellow arrowheads, apical side. **I** Western blots used in Figure 4**E**; red rectangles outline the cropped images used.
Data information: Scale bar = 10 µm in (**G, H**). (**D**) 200 cells from 2-3 mice in each group; (**E, F**) >150 cells from 2 mice for each group. (**H**) 20 cells from 2 mice each group. Bars represent medians ± 95% CI. Kruskal–Wallis test followed by Dunn's multiple comparisons test in (**D, H**); Chi-square test in (**E, F**).

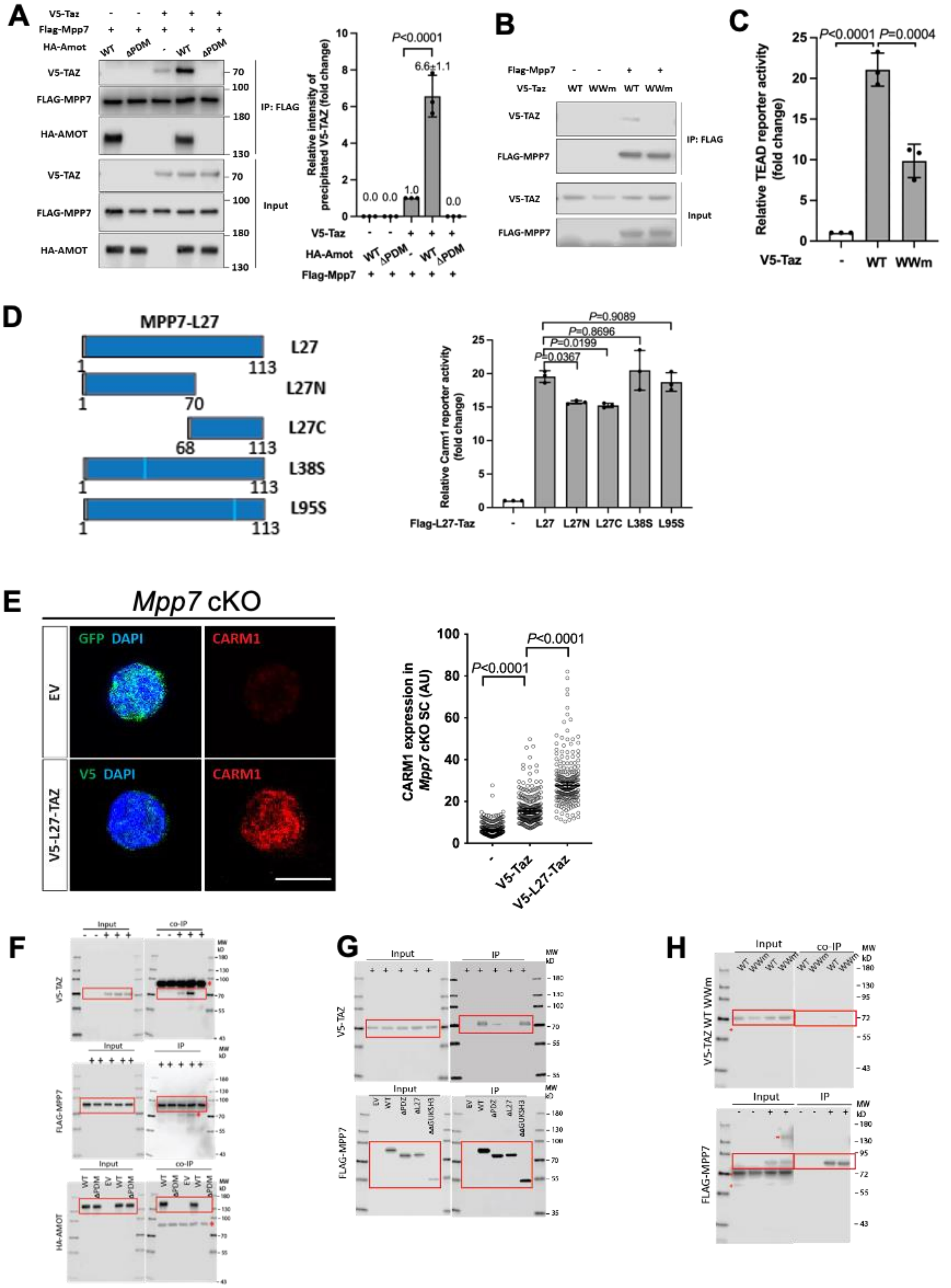

Appendix Figure S5: Additional data to support Fig. 5.

A MPP7-TAZ interaction is enhanced by WT AMOT, but disrupted by ΔPDM AMOT using the co-IP assay in 293T cells. Expression constructs and tagged epitopes for detection are indicated; (-), empty expression construct; quantification to the right. **B**

Mutation in the AMOT-interacting WW-domain of TAZ abolishes MPP7-TAZ interaction. Expression constructs and tagged epitopes for detection are indicated as in (**A**); WWm, WW domain of TAZ with a point mutation that disrupts its interaction with AMOT. N = 2. **C** Quantification of TEAD-reporter activity by empty (-), WT Taz and Taz WWm expression vectors. **D** MPP7's L27 domain has two L27 motifs, L27N and L27C. Taz-fusion constructs with either L27N or L27C were made. Taz-fusion constructs with L38S or L95S a.a. substitution in the L27 domain were also made. They were tested on the Carm1-reporter; expression constructs in the x-axis; (-), empty expression construct. **E** Empty (with IRES-GFP), V5-TAZ (-/V5-Taz from Figure 4**H**) and V5-L27-Taz vectors were transfected into cultured *Mpp7* cKO cells. IF of GFP or V5, together with CARM1 were performed (representative images on the left; Same images for V5-Taz already in Fig. S4f) and signals (AU) of CARM1 were quantified. **F** Western blots used in (**A**); red rectangles outline the cropped images used, and red asterisks non-specific bands. **G** Western blots used in Fig. 5**A**; red rectangles outline the cropped images used. **H** Western blots used in (**B**); red rectangles outline the cropped images used, and red asterisks non-specific bands.
Data information: Scale bar = 10 µm in (**E**). (**A, C, D**) n=3 biological replicates. (**E**) 200 cells in each group. Bars represent means ± SD in (**A, C, D**) or medians ± 95% CI in (**E**). One-way ANOVA with Tukey's post hoc test in (**A, C, D**); Kruskal–Wallis test followed by Dunn's multiple comparisons test in (**E**).

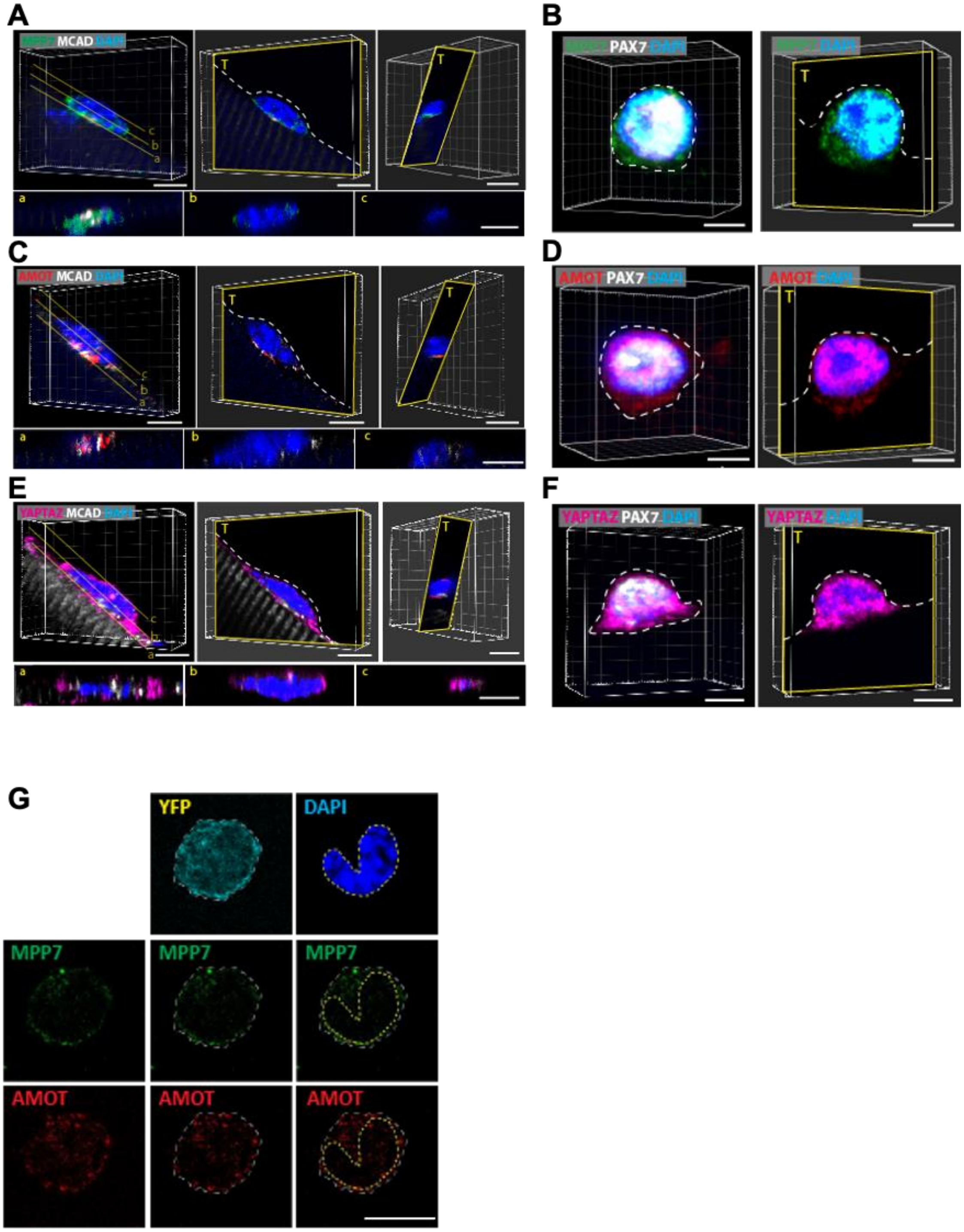

Appendix Figure S6: Additional data to support Fig. 6.
A-F Re-confirmation of MPP7 (**A**, **B**), AMOT (**C**, **D**), and YAP/TAZ (**E**, **F**) cellular localization in MuSCs at 0 h (**A**, **C**, **E**) and 48 h (**B**, **D**, **F**) by SM assay. Similar results were published(Li & Fan, 2017). Here, in addition to DAPI counter staining (for the nucleus), we also included co-IF with MCAD (in **A**, **C**, and **E** for apical membrane) or PAX7 (in **B**, **D**, and **F** for the nucleus) for sub-cellular localization assignment. Shown

are representative 3D reconstructed images with select single planes (as indicated). **G** An example for how nuclear MPP7 and AMOT immunostaining signals were quantified. FACS-isolated YFP-marked MuSCs were used. DAPI was used for counter stain to define the area of the nucleus (yellow dashed lines), and YFP was used to define the total area of a cell (white dashed lines). The percentage of nuclear signal is that of the signal overlapped with DAPI divided by total signals within YFP-marked area. Same images were shown in Fig. 6C (DMSO control).
Data information: Scale bars = 5 µm in (**A**-**F**) and 10 µm in (**F**).

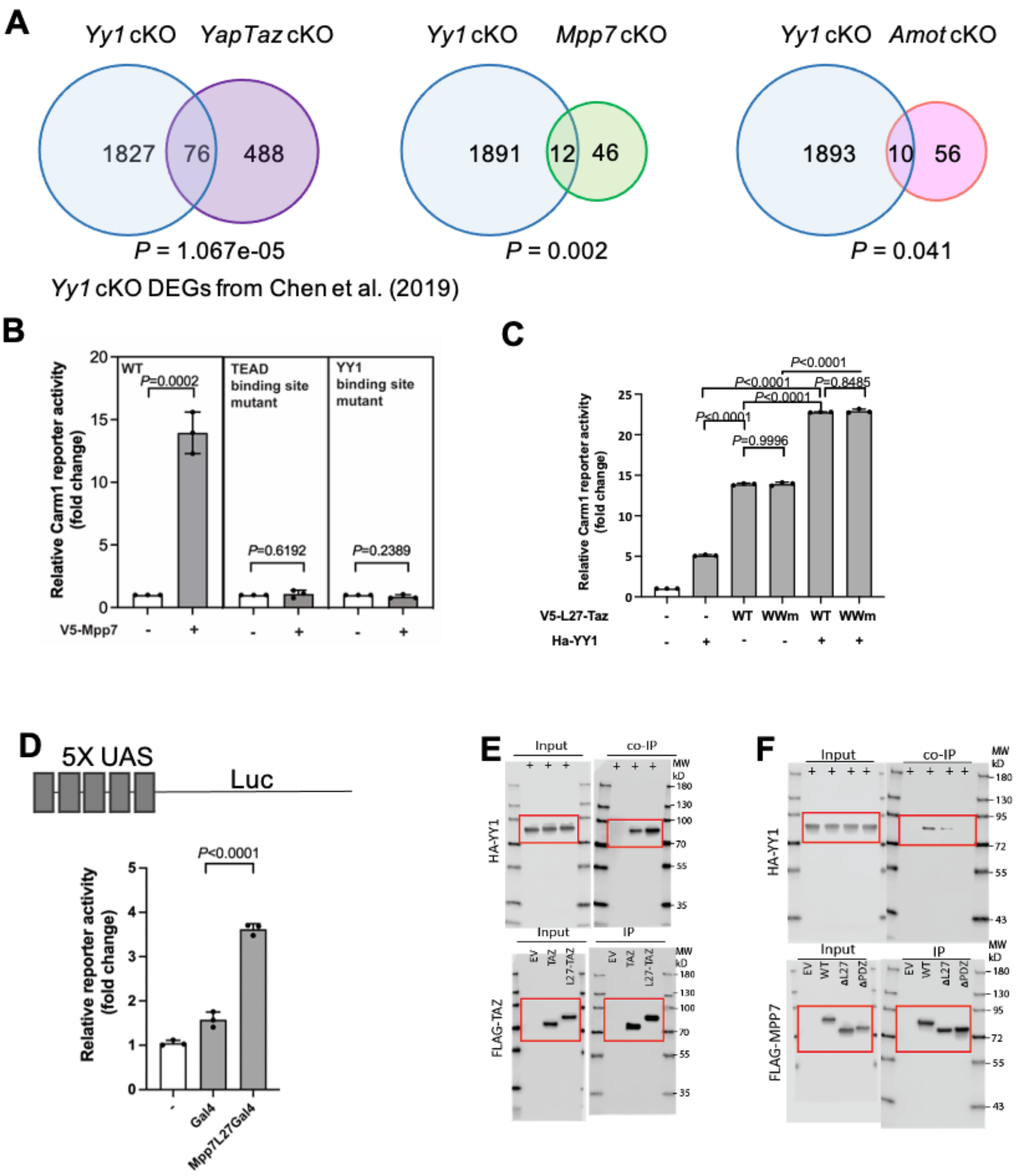

Appendix Figure S7: Additional data to support Fig. 7.

A Venn Diagrams of DEGs between the *Yy1* cKO and the *YapTaz* cKO, the *Yy1* cKO and the *Mpp7* cKO, and the *Yy1* cKO and the *Amot* cKO. The data set of *Yy1* cKO was performed at 36 h of culture(Chen *et al*, 2019). **B** WT, TEAD binding site mutated, and YY1 binding site mutated Carm1-reporters were tested for response to the V5-Mpp7 expression construct; (-), empty expression construct. **C** WT Carm1-reporter was tested for response to L27-Taz or L27-Taz with a mutated WW domain (WWm, no longer interacting with AMOT), with or without YY1 co-expression (x-axis); (-), empty expression construct. **D** Mpp7-L27 is a transcriptional activation domain when fused to Gal4 (Gal4-DBD); Gal4-UAS luciferase (Luc) reporter is diagrammed at the top. **E** Western blots used in Fig. 7**E**; red rectangles outline the cropped images used. **F** Western blots used in Fig. 7**F**; red rectangles outline the cropped images used.

Data information: Error bars represent means ± SD. (**B-D**) n=3 biological replicates. Hypergeometric test was performed in (**A**). Student's *t*-tests (two-sided) were performed in (**B**). One-way ANOVA with Tukey's post hoc test was performed in (**C, D**).

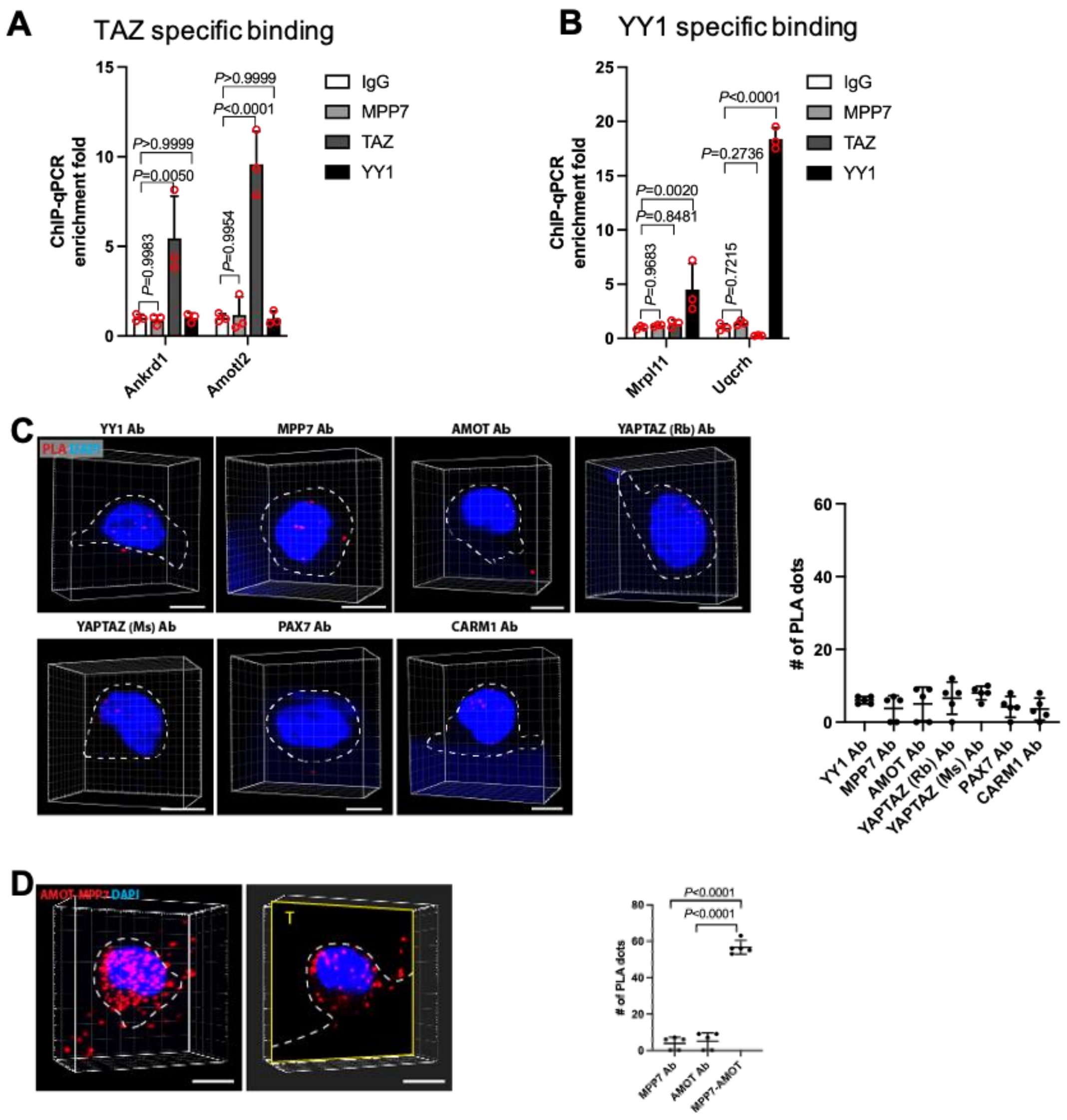

Appendix Figure S8: Additional data to support Fig. 8.
A ChIP-qPCR shows TAZ-specific binding to promoter regions of 2 DEGs(Zanconato *et al*, 2015) (x-axis) not shared with those of *Yy1*, *Amot*, or *Mpp7* cKOs. **B** ChIP-qPCR shows YY1-specific binding to promoter regions of 2 DEGs(Chen *et al.*, 2019) (x-axis) not shared with those of *YapTaz*, *Amot*, or *Mpp7* cKOs. In (**A**, **B**), keys to ChIP are to the right; IgG served as negative control. Bar graph showed fold-enrichment calculated by the Bio-Rad CFX Maestro software package. **C** Representative 3D images for each single antibody used for PLA reaction on MuSCs at 48 h in SM culture, as controls; quantification to the right. **D** Re-confirmation of AMOT-MPP7 association by PLA assay (3D and one plane); quantification to the right. Each black dot represents one cell with the number of PLA red dots quantified using 3D reconstructed images.
Data information: Scale bars = 5 µm. (**A, B**) n=3 biological replicates; (**C, D**) n=5 cells for each group. Error bars represent means ± SD. One-way ANOVA with Tukey's post hoc test was performed.

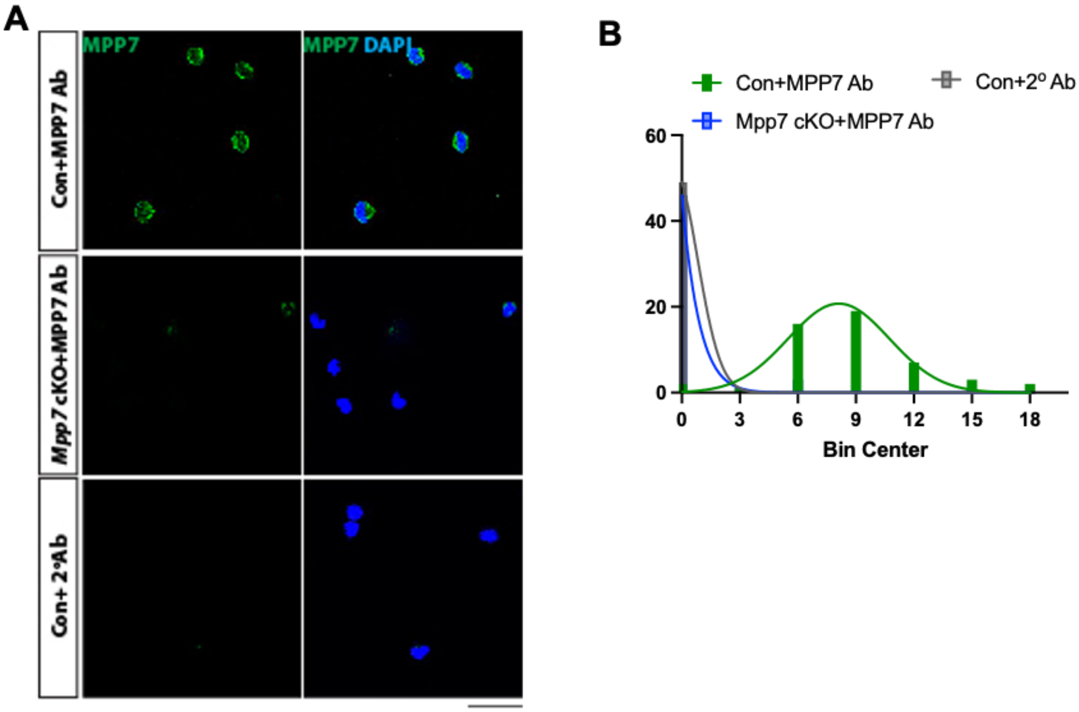

Appendix Fig. S9: Antibody validation and background threshold.
a IF images of FACS-isolated Con MuSCs (*Pax7*$^{CreERT2/+}$; top panel) stained with anti-MPP7 antibodies (Ab) and Alexa-488 conjugated secondary (2°) antibodies, *Mpp7* cKO MuSCs (middle panel) stained with anti-MPP7 Ab and 2° Ab, and Con MuSCs with secondary Ab (2^oAb) only. Note that we specifically included a rare MPP7$^+$ cell in the *Mpp7* cKO MuSCs to show our ability to distinguish positive vs negative signals and determine the KO efficiency. **b** Spectra distributions from stained cell populations in (**a**) were plotted to determine positive and negative signal cutoff value, i.e. background threshold.
Data information: Scale bars = 25 µm.

Reference

Chen F, Zhou J, Li Y, Zhao Y, Yuan J, Cao Y, Wang L, Zhang Z, Zhang B, Wang CC *et al* (2019) YY1 regulates skeletal muscle regeneration through controlling metabolic reprogramming of satellite cells. *EMBO J* 38

Fishilevich S, Nudel R, Rappaport N, Hadar R, Plaschkes I, Iny Stein T, Rosen N, Kohn A, Twik M, Safran M *et al* (2017) GeneHancer: genome-wide integration of enhancers and target genes in GeneCards. *Database (Oxford)* 2017

Keenan AB, Torre D, Lachmann A, Leong AK, Wojciechowicz ML, Utti V, Jagodnik KM, Kropiwnicki E, Wang Z, Ma'ayan A (2019) ChEA3: transcription factor enrichment analysis by orthogonal omics integration. *Nucleic Acids Res* 47: W212-W224

Li L, Fan CM (2017) A CREB-MPP7-AMOT Regulatory Axis Controls Muscle Stem Cell Expansion and Self-Renewal Competence. *Cell Rep* 21: 1253-1266

Sun C, De Mello V, Mohamed A, Ortuste Quiroga HP, Garcia-Munoz A, Al Bloshi A, Tremblay AM, von Kriegsheim A, Collie-Duguid E, Vargesson N *et al* (2017) Common and Distinctive Functions of the Hippo Effectors Taz and Yap in Skeletal Muscle Stem Cell Function. *Stem Cells* 35: 1958-1972

Zanconato F, Forcato M, Battilana G, Azzolin L, Quaranta E, Bodega B, Rosato A, Bicciato S, Cordenonsi M, Piccolo S (2015) Genome-wide association between YAP/TAZ/TEAD and AP-1 at enhancers drives oncogenic growth. *Nat Cell Biol* 17: 1218-1227
