# Supplementary figures and images for: The L27 domain of MPP7 enhances TAZ-YY1 cooperation to renew muscle stem cells (part 1 of 2)
Source: EMBO Rep. 2024 Nov 4;25(12):5667–86. doi: 10.1038/s44319-024-00305-4 (PMC11624273; doi:10.1038/s44319-024-00305-4)

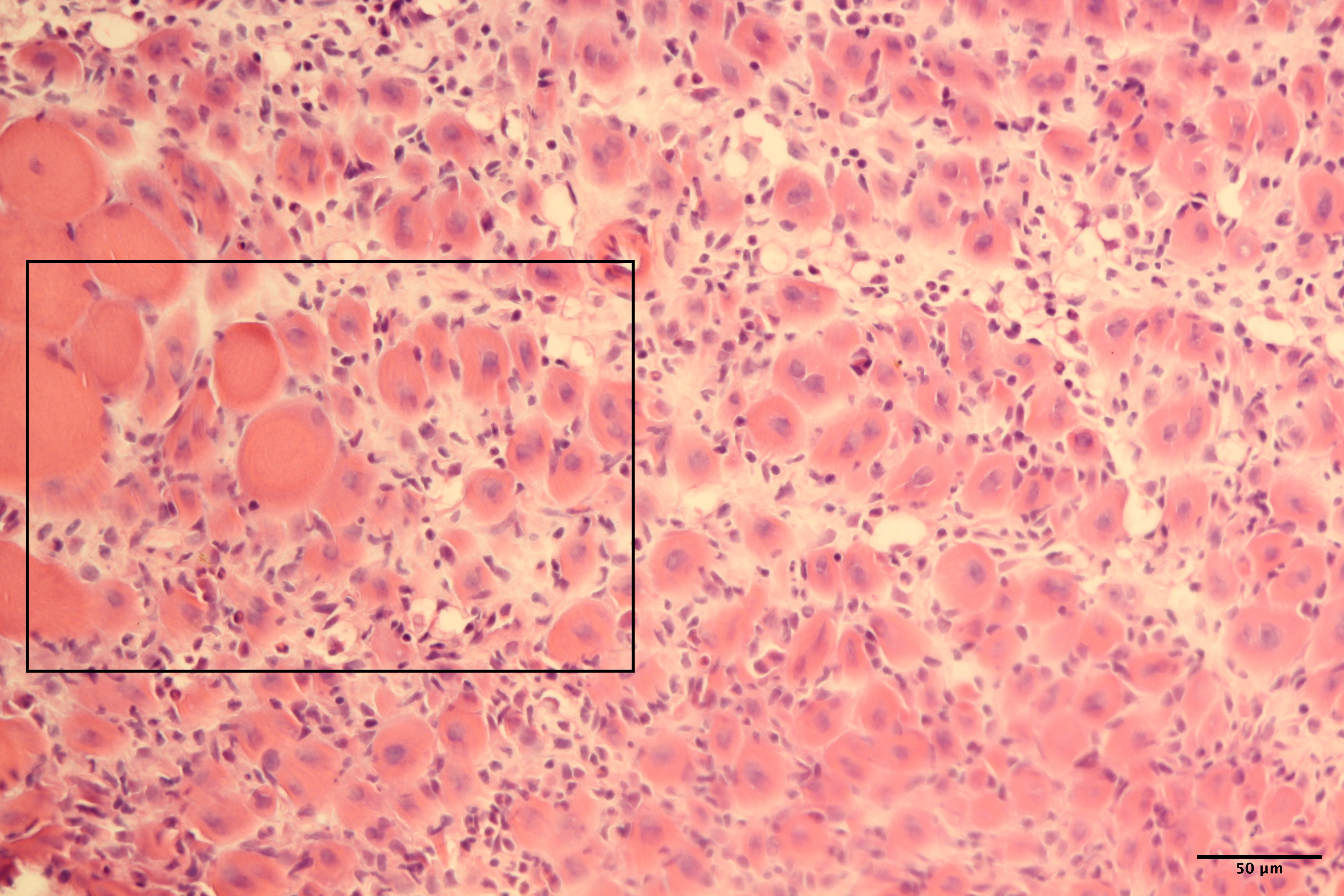

Supplement: Supplementary file 4 — Source data Fig. 1 [file 44319_2024_305_MOESM4_ESM.zip › Figure 1/1C Con Mpp7 histology/1C con 5dpi.jpg]

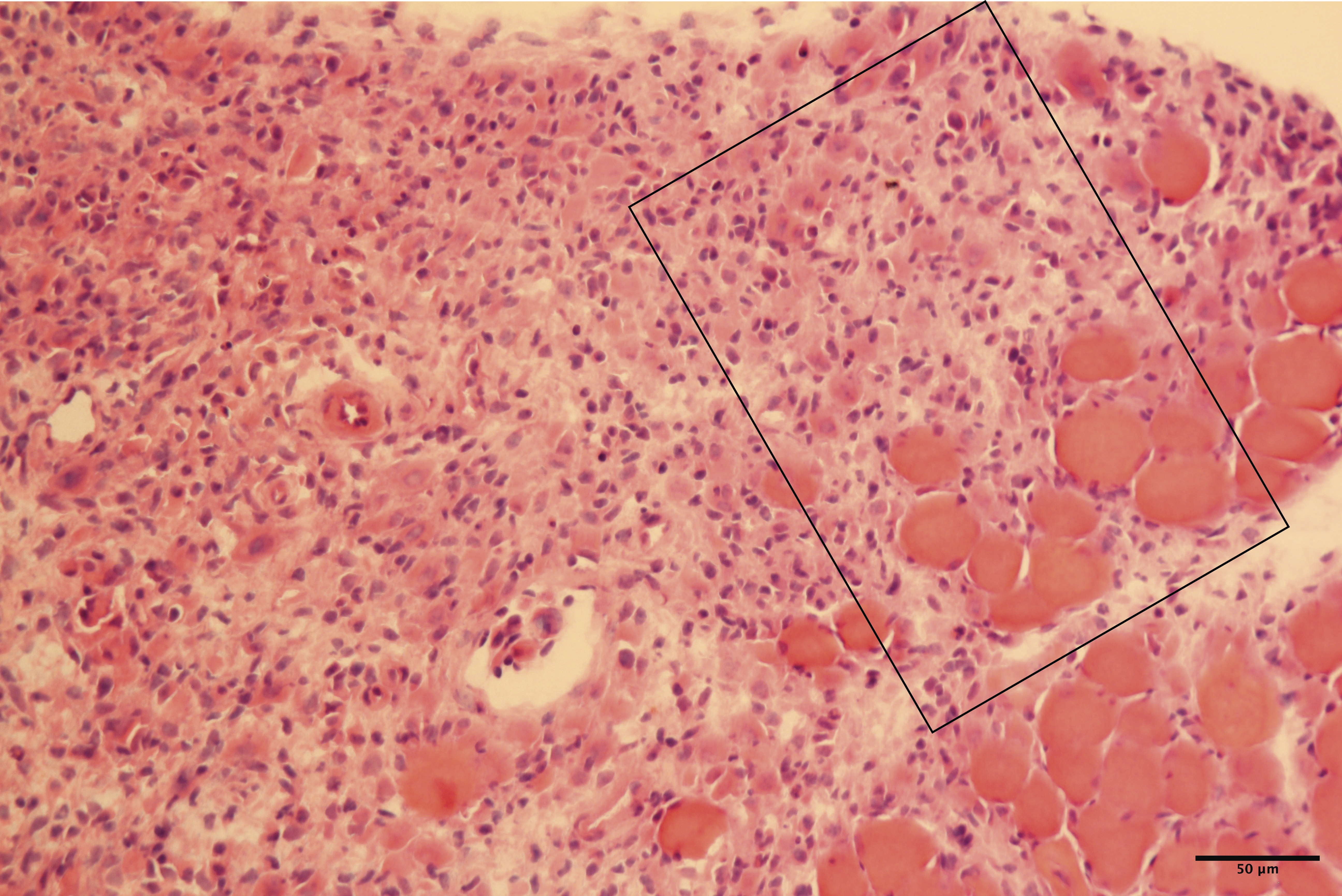

Supplement: Supplementary file 4 — Source data Fig. 1 [file 44319_2024_305_MOESM4_ESM.zip › Figure 1/1C Con Mpp7 histology/1C M7cKO 5dpi.jpg]

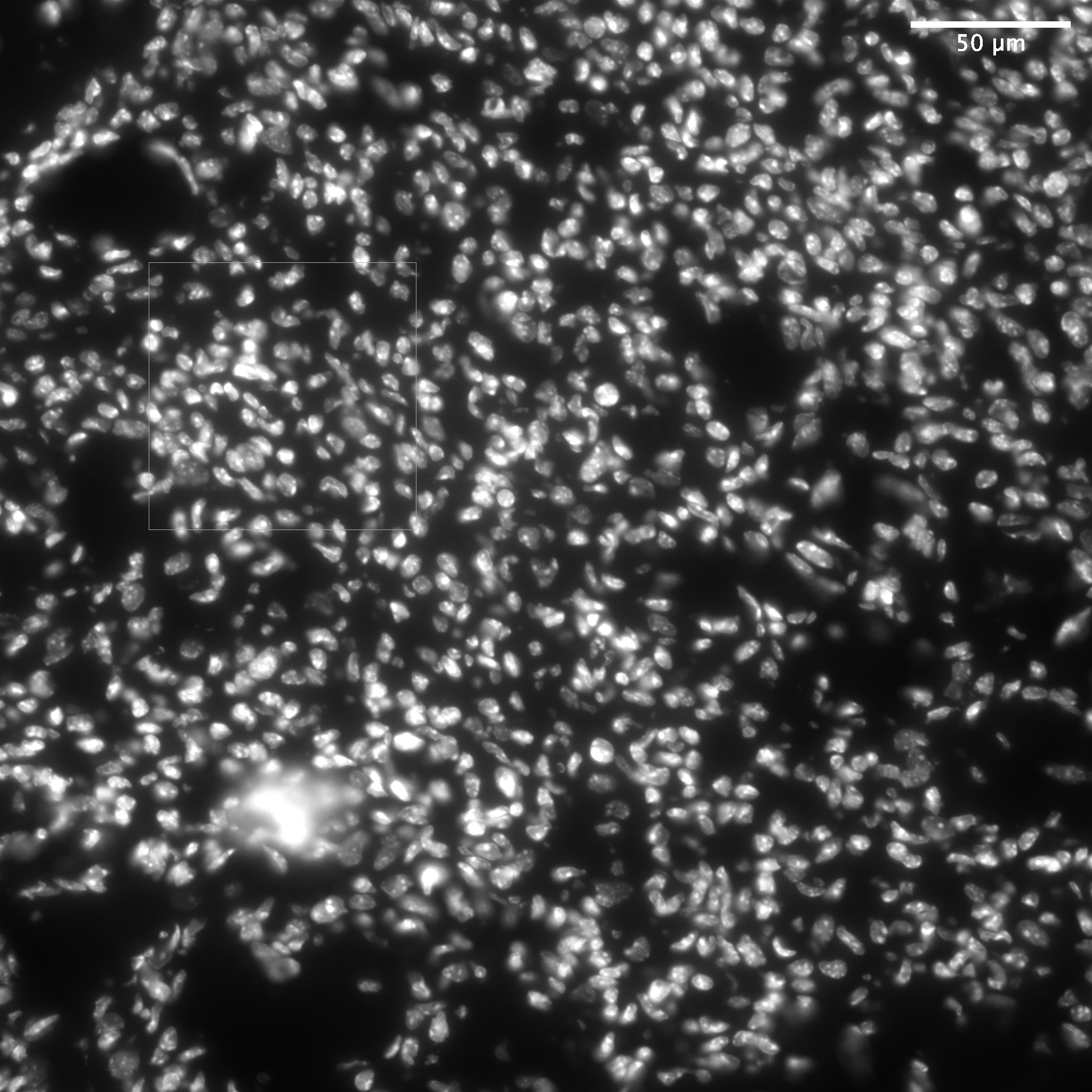

Supplement: Supplementary file 4 — Source data Fig. 1 [file 44319_2024_305_MOESM4_ESM.zip › Figure 1/1D Con Mpp7 5dpi IF/Mpp7 cKO 5 dpi/Figure 1D. Mpp7cKO_DAPI_5 dpi.tif]

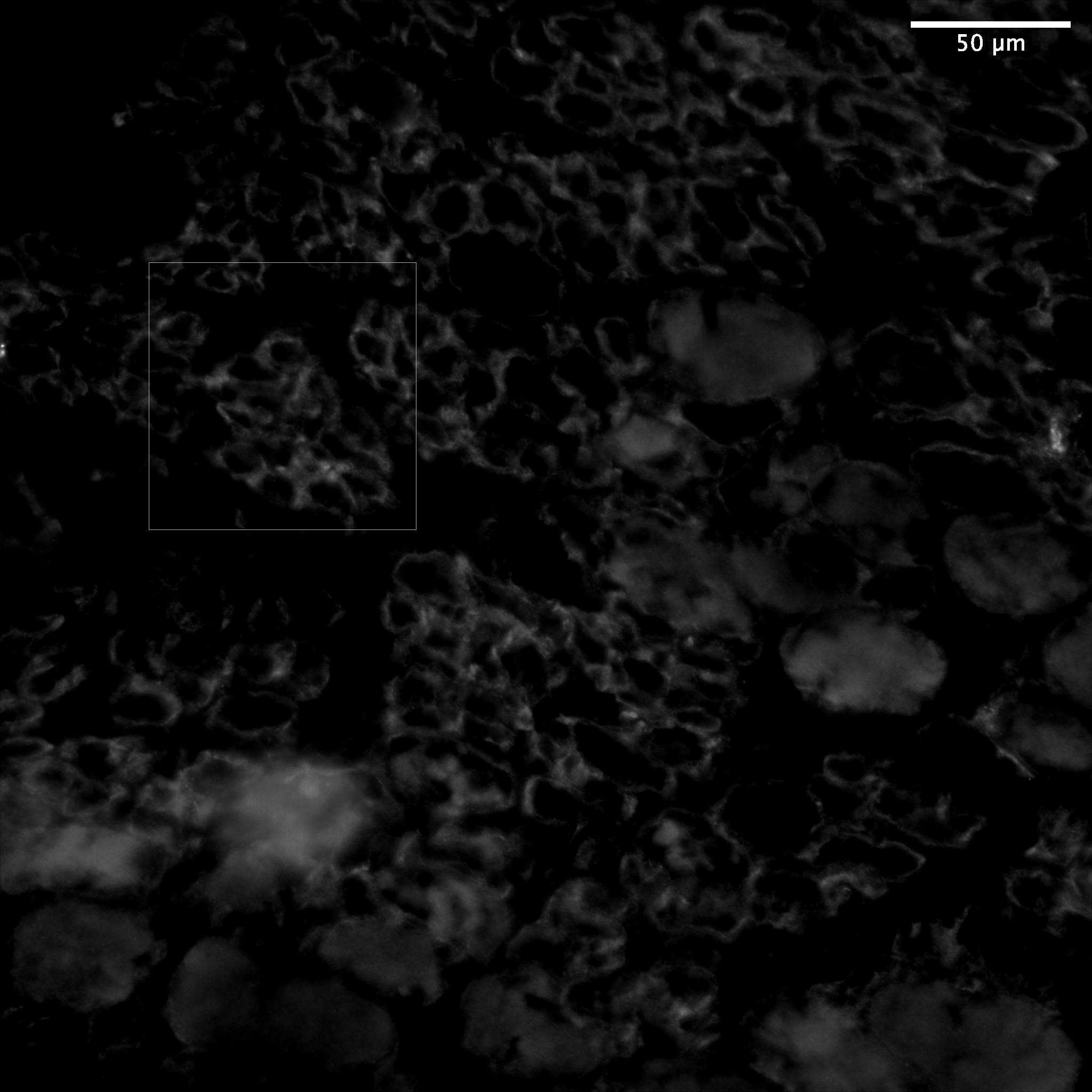

Supplement: Supplementary file 4 — Source data Fig. 1 [file 44319_2024_305_MOESM4_ESM.zip › Figure 1/1D Con Mpp7 5dpi IF/Mpp7 cKO 5 dpi/Figure 1D. Mpp7cKO_Laminin_5 dpi.tif]

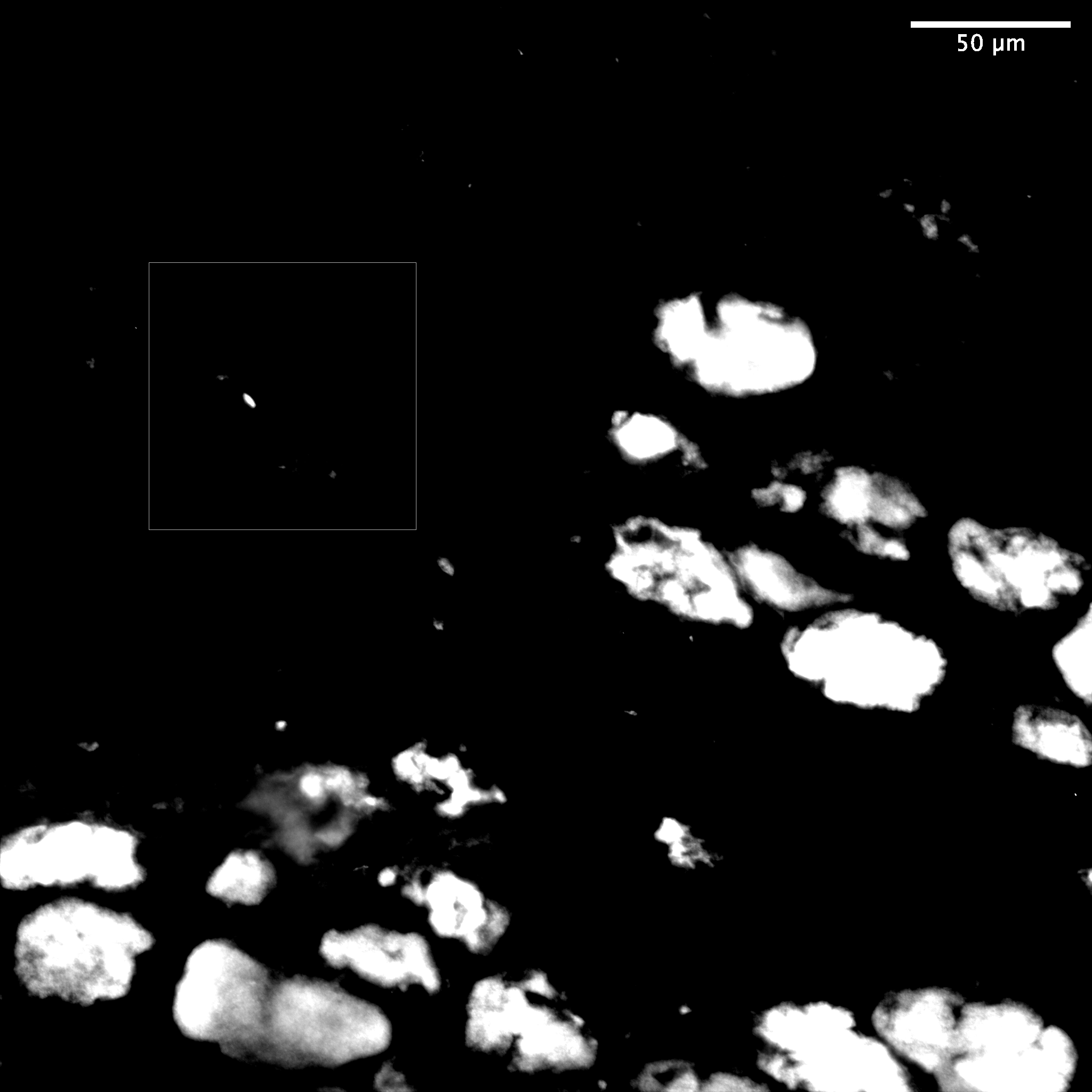

Supplement: Supplementary file 4 — Source data Fig. 1 [file 44319_2024_305_MOESM4_ESM.zip › Figure 1/1D Con Mpp7 5dpi IF/Mpp7 cKO 5 dpi/Figure 1D. Mpp7cKO_Pax7_5 dpi.tif]

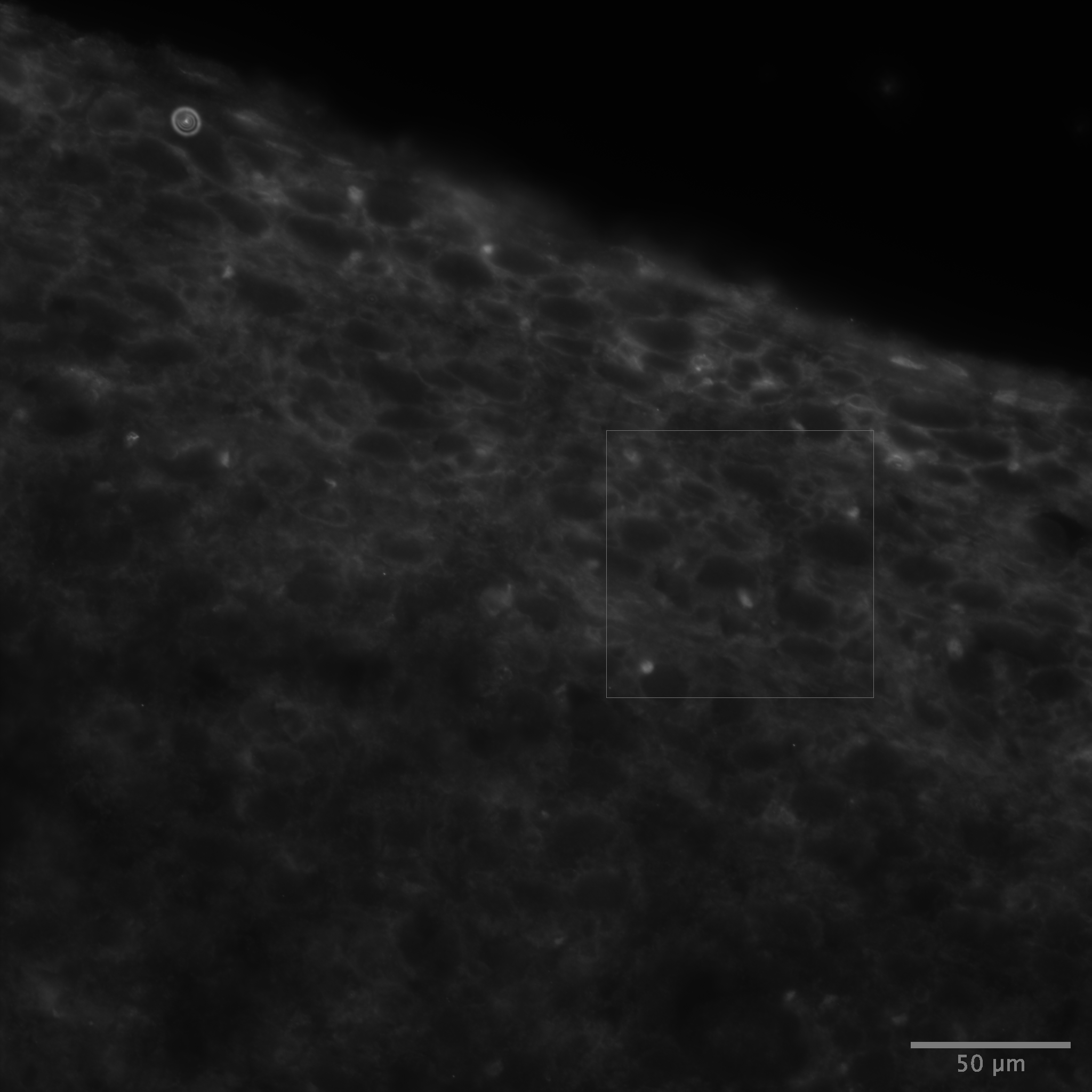

Supplement: Supplementary file 4 — Source data Fig. 1 [file 44319_2024_305_MOESM4_ESM.zip › Figure 1/1D Con Mpp7 5dpi IF/Con 5 dpi/Figure 1D. Con_Pax7_5 dpi.tif]

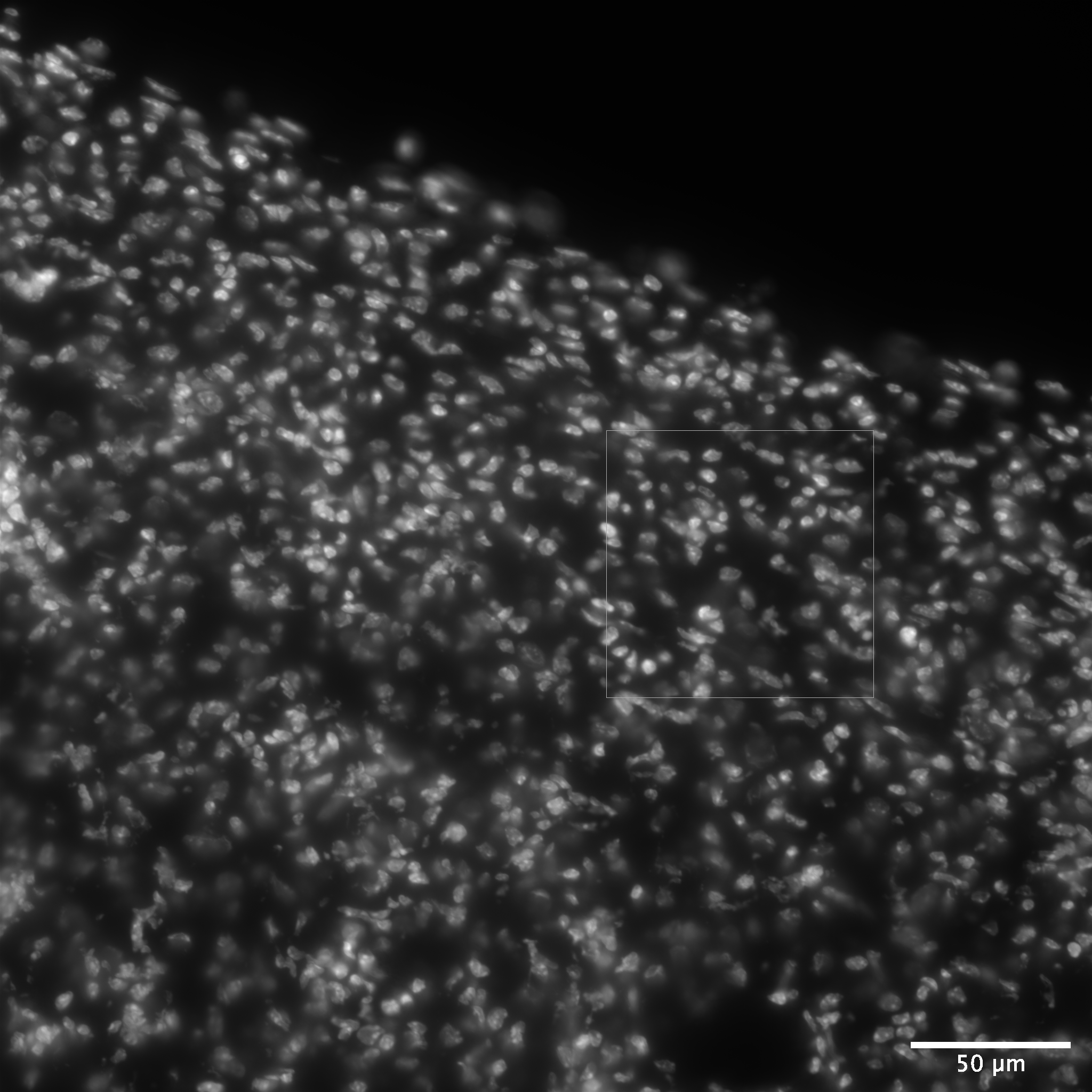

Supplement: Supplementary file 4 — Source data Fig. 1 [file 44319_2024_305_MOESM4_ESM.zip › Figure 1/1D Con Mpp7 5dpi IF/Con 5 dpi/Figure 1D. Con DAPI 5 dpi .tif]

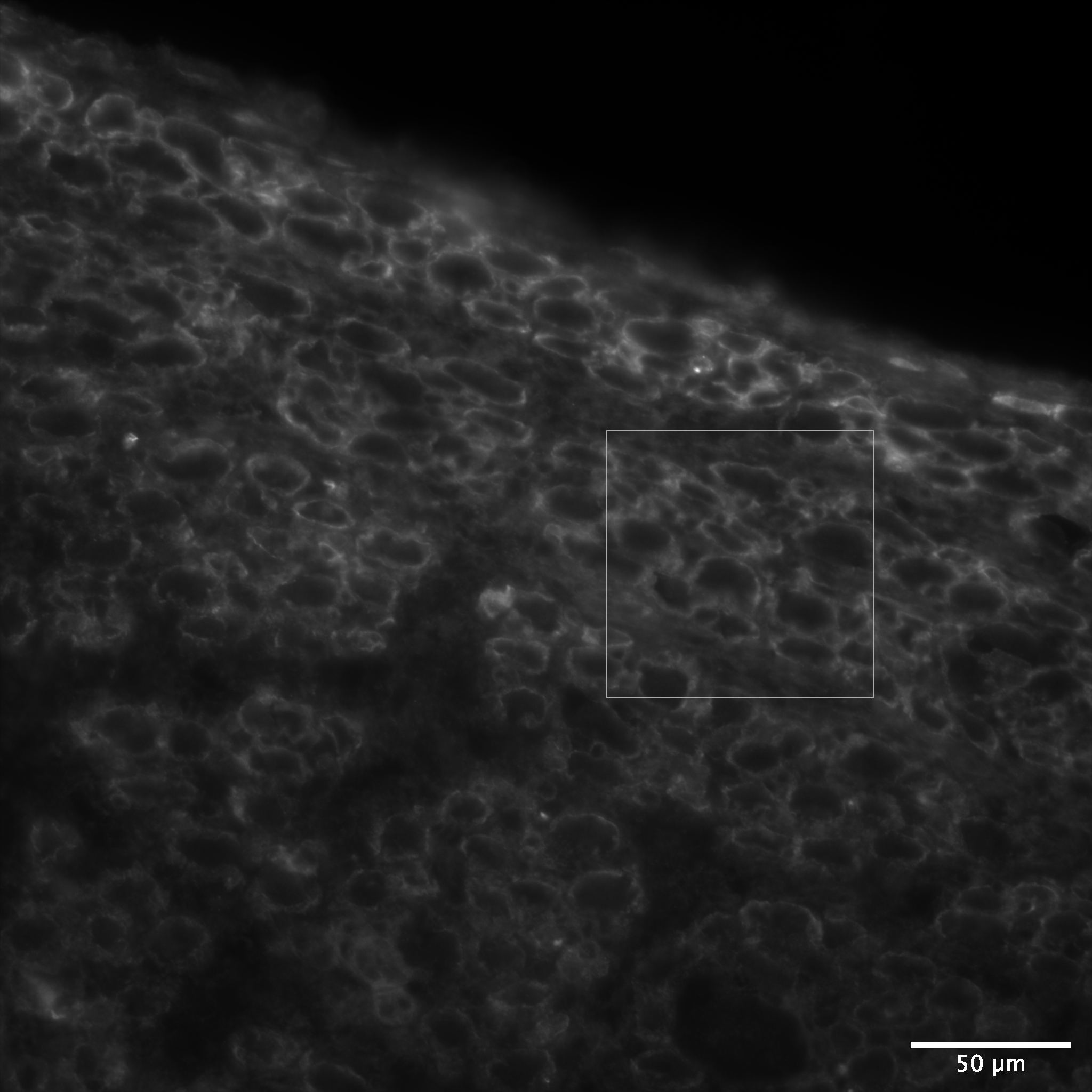

Supplement: Supplementary file 4 — Source data Fig. 1 [file 44319_2024_305_MOESM4_ESM.zip › Figure 1/1D Con Mpp7 5dpi IF/Con 5 dpi/Figure 1D. Con_Laminin_5 dpi.tif]

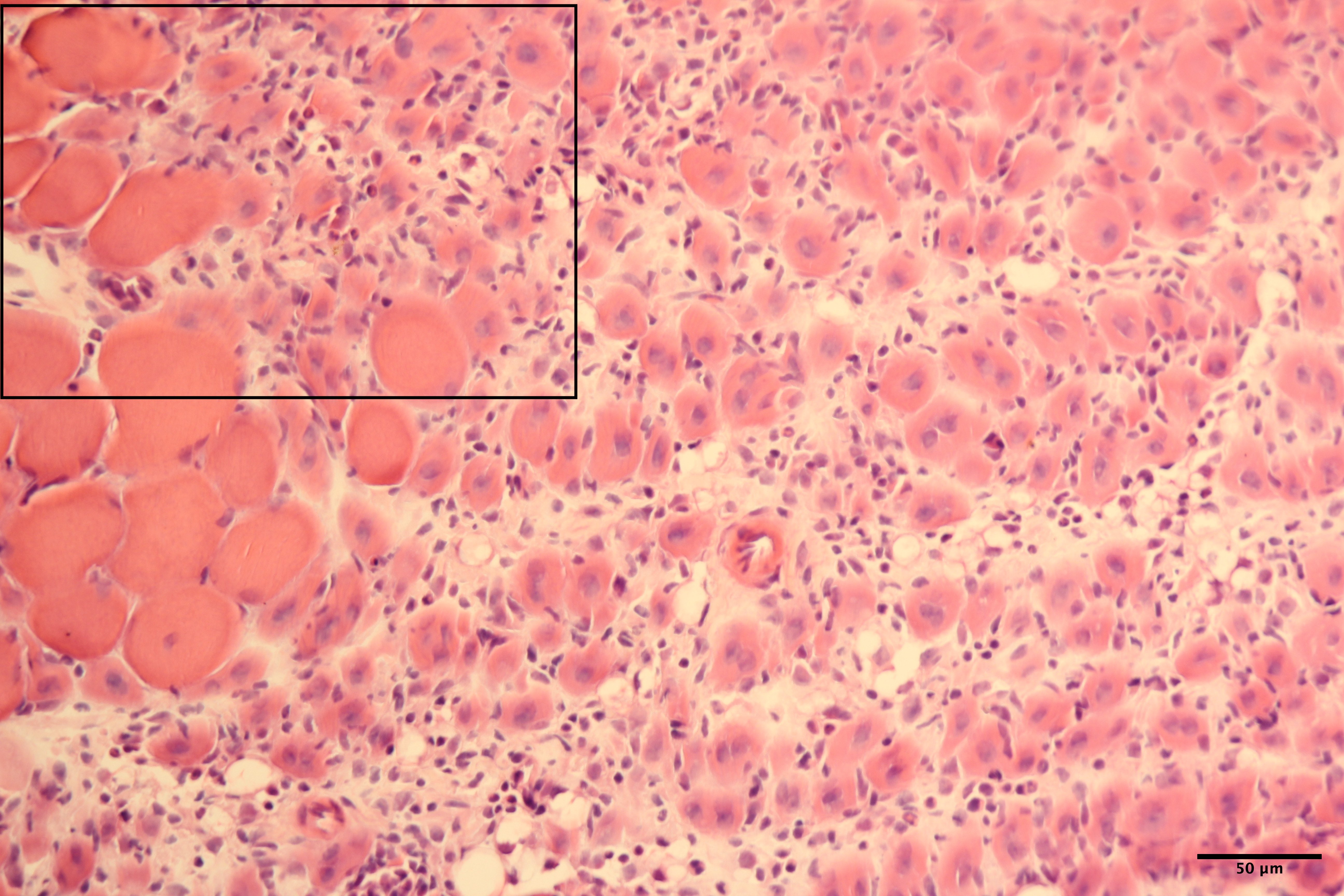

Supplement: Supplementary file 5 — Source data Fig. 2 [file 44319_2024_305_MOESM5_ESM.zip › Figure 2/2E Con Amot cKO histology/2E Con.jpg]

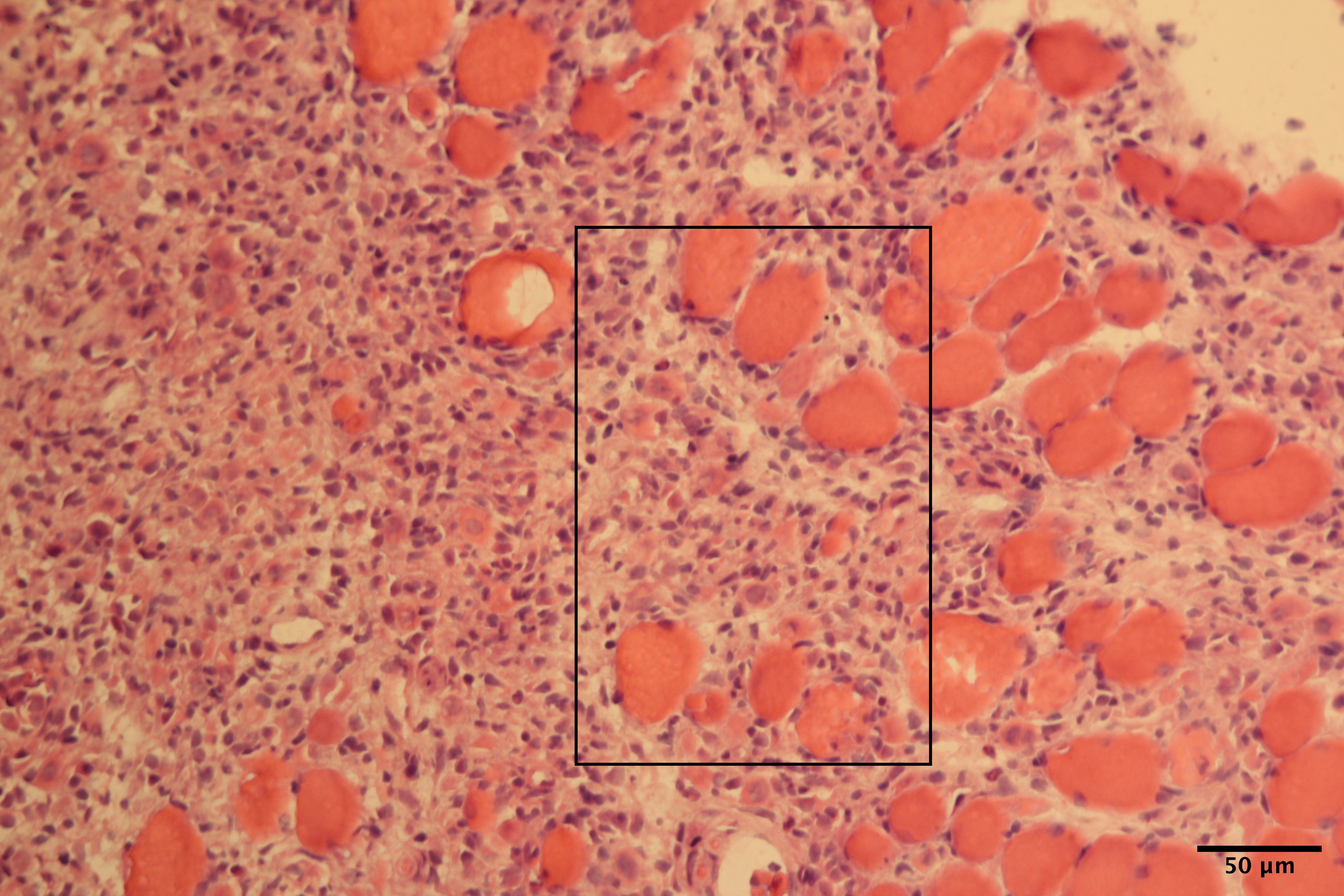

Supplement: Supplementary file 5 — Source data Fig. 2 [file 44319_2024_305_MOESM5_ESM.zip › Figure 2/2E Con Amot cKO histology/2E Ag cko.jpg]

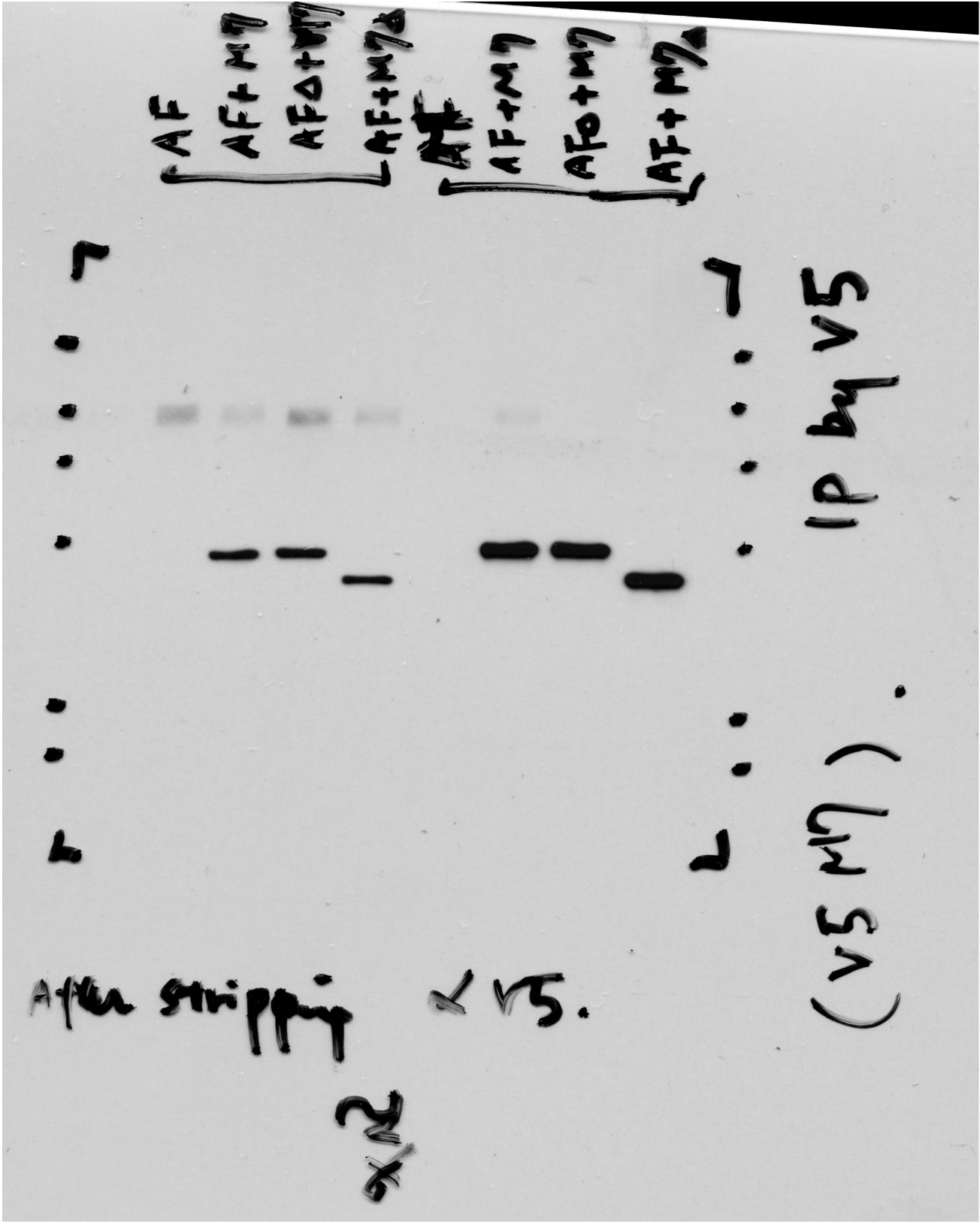

Supplement: Supplementary file 5 — Source data Fig. 2 [file 44319_2024_305_MOESM5_ESM.zip › Figure 2/2D Mpp7 Amot coIP/2D. blotting V5-M7.png]

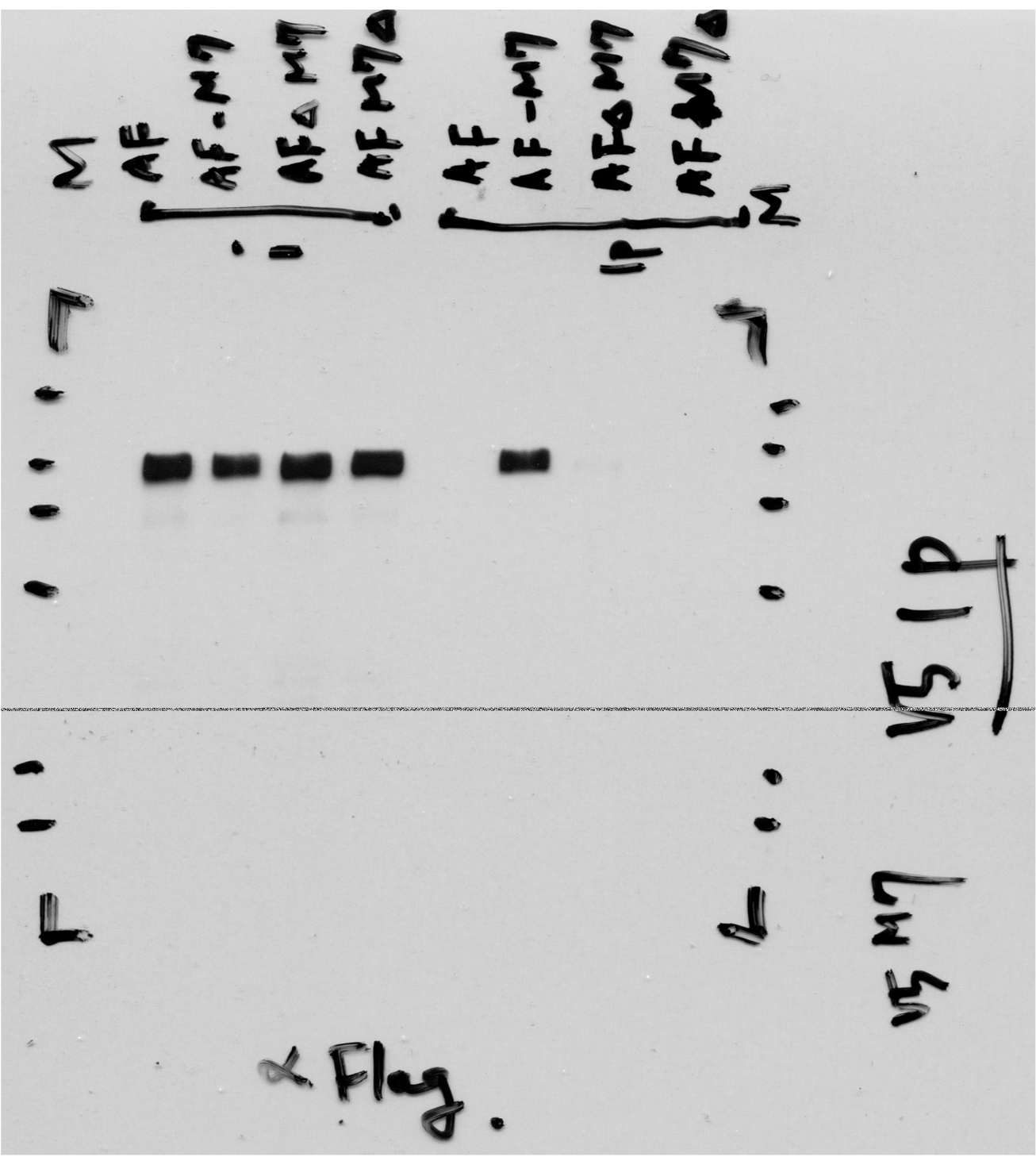

Supplement: Supplementary file 5 — Source data Fig. 2 [file 44319_2024_305_MOESM5_ESM.zip › Figure 2/2D Mpp7 Amot coIP/2D. blotting Ha-Ag.png]

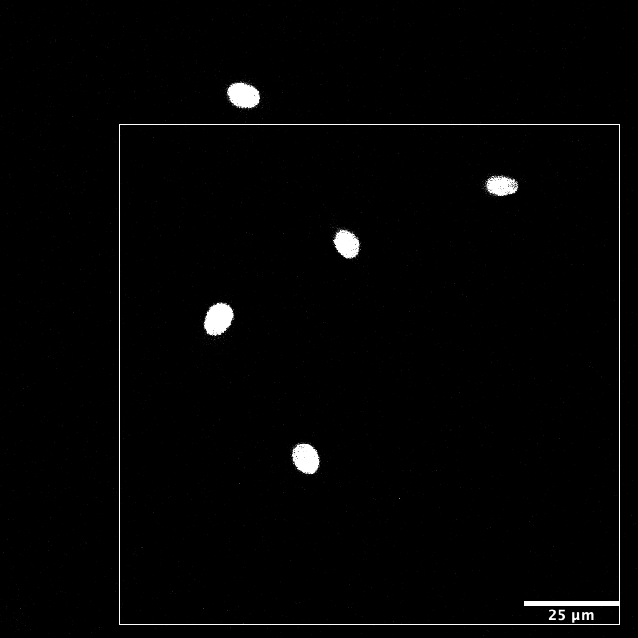

Supplement: Supplementary file 5 — Source data Fig. 2 [file 44319_2024_305_MOESM5_ESM.zip › Figure 2/2J Mpp7 expression in Con Amot cKO/Con IF/Control MuSCs_DAPI.tif]

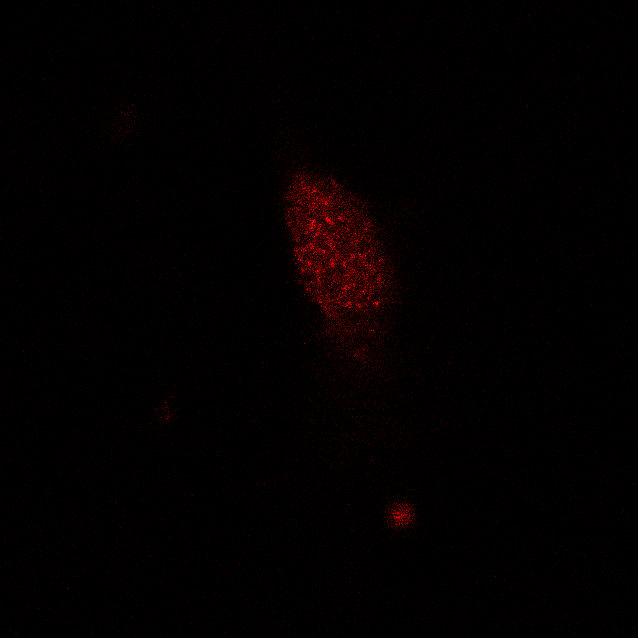

Supplement: Supplementary file 5 — Source data Fig. 2 [file 44319_2024_305_MOESM5_ESM.zip › Figure 2/2K Amot expression in Con Mpp7 cKO/Con IF/Control MuSCs 5X_AMOT.tif]

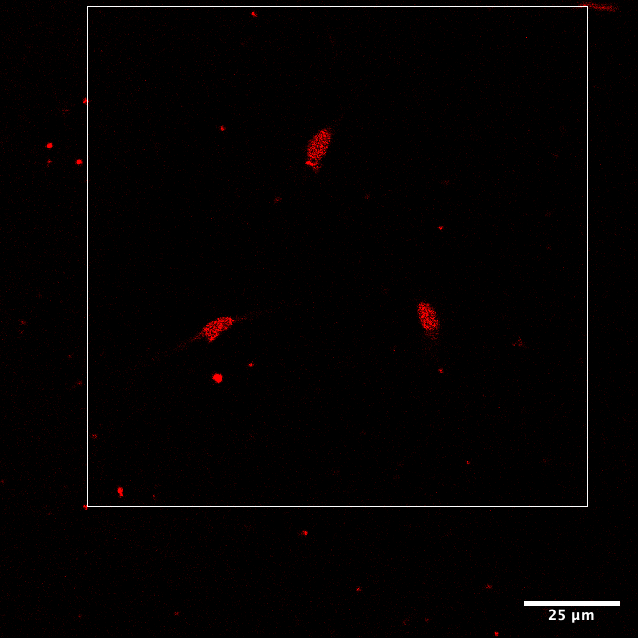

Supplement: Supplementary file 5 — Source data Fig. 2 [file 44319_2024_305_MOESM5_ESM.zip › Figure 2/2K Amot expression in Con Mpp7 cKO/Con IF/Control MuSCs_AMOT.tif]

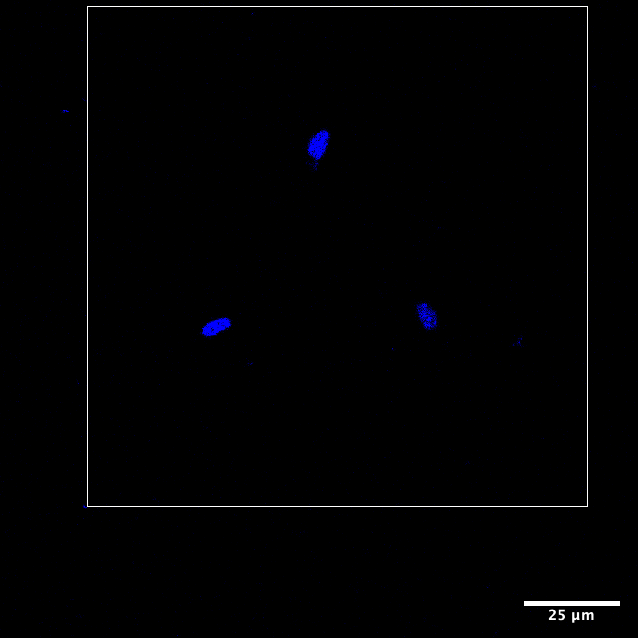

Supplement: Supplementary file 5 — Source data Fig. 2 [file 44319_2024_305_MOESM5_ESM.zip › Figure 2/2K Amot expression in Con Mpp7 cKO/Con IF/Control MuSCs_DAPI.tif]

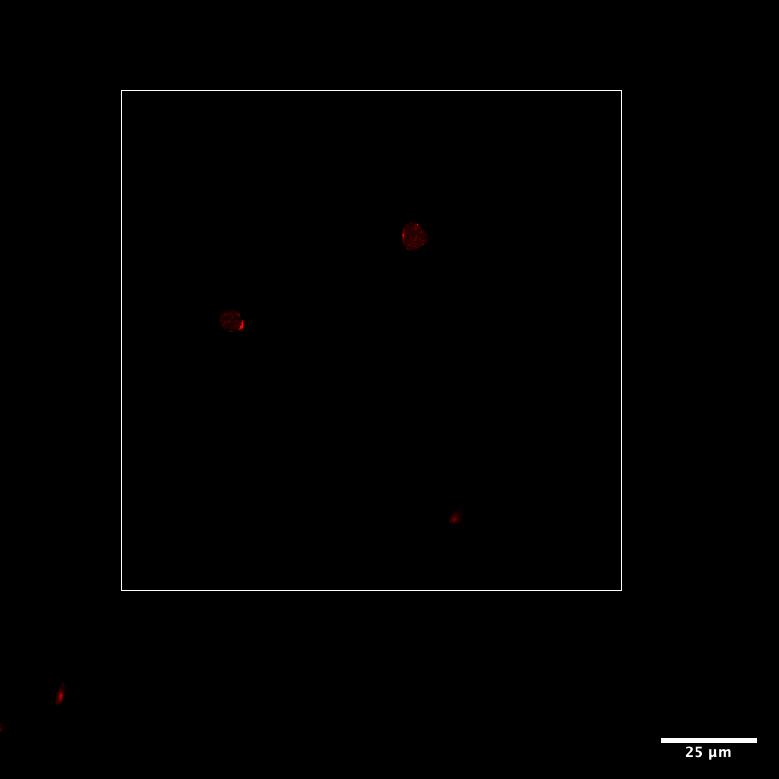

Supplement: Supplementary file 5 — Source data Fig. 2 [file 44319_2024_305_MOESM5_ESM.zip › Figure 2/2K Amot expression in Con Mpp7 cKO/Mpp7 cKO IF/Mpp7cKO MuSCs_AMOT.tif]

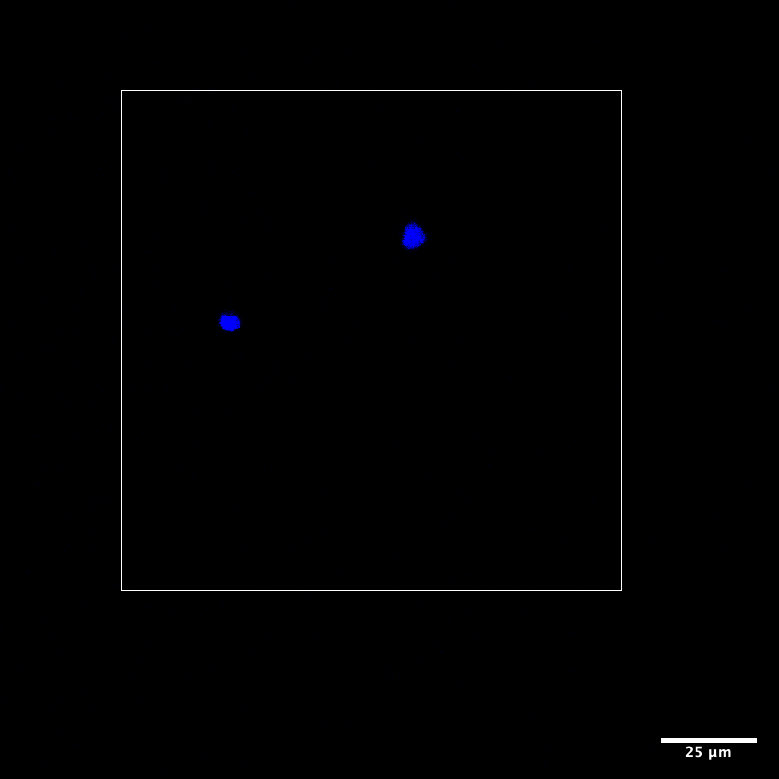

Supplement: Supplementary file 5 — Source data Fig. 2 [file 44319_2024_305_MOESM5_ESM.zip › Figure 2/2K Amot expression in Con Mpp7 cKO/Mpp7 cKO IF/Mpp7cKO MuSCs_DAPI.tif]

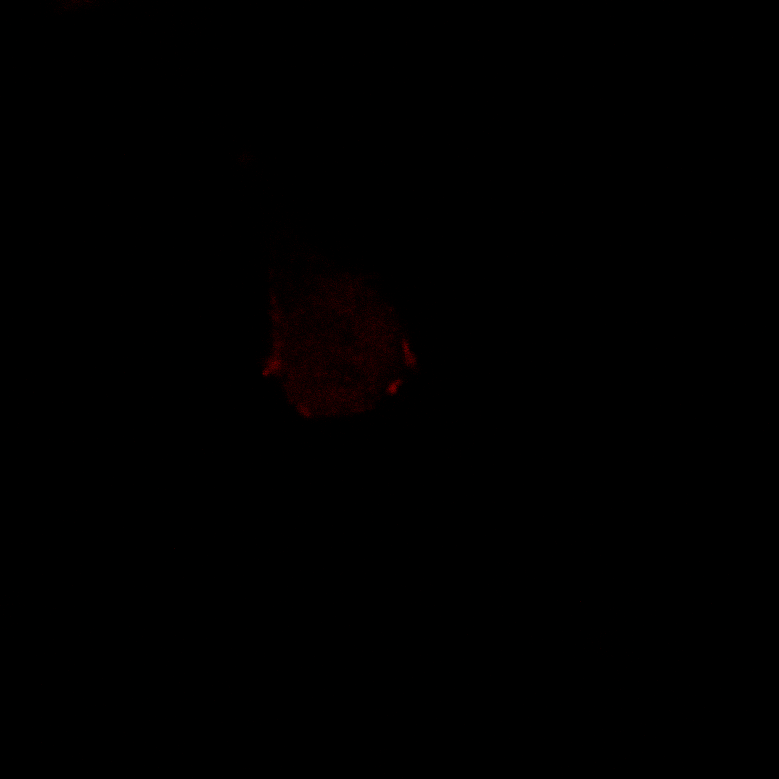

Supplement: Supplementary file 5 — Source data Fig. 2 [file 44319_2024_305_MOESM5_ESM.zip › Figure 2/2K Amot expression in Con Mpp7 cKO/Mpp7 cKO IF/Mpp7cKO MuSCs 5X_AMOT.tif]

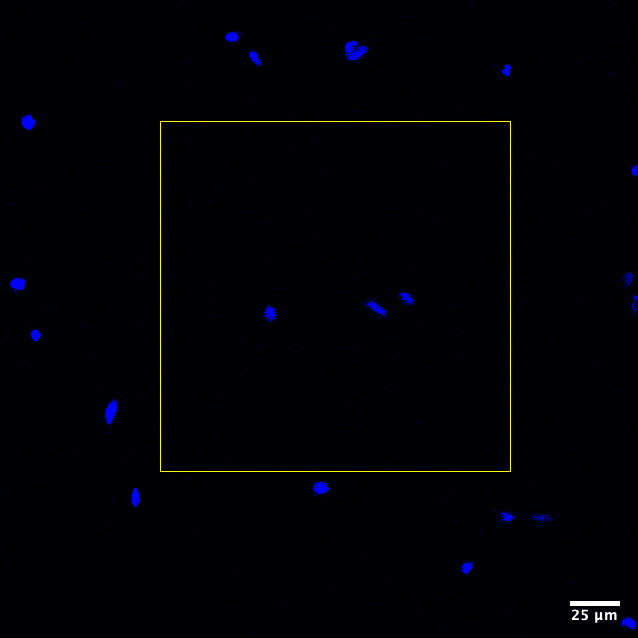

Supplement: Supplementary file 6 — Source data Fig. 3 [file 44319_2024_305_MOESM6_ESM.zip › Figure 3/3F/Mpp7 cKO/Figure 3F. Representative images of CARM1 expression in Mpp7cKO MuSCs_DAPI.tif]

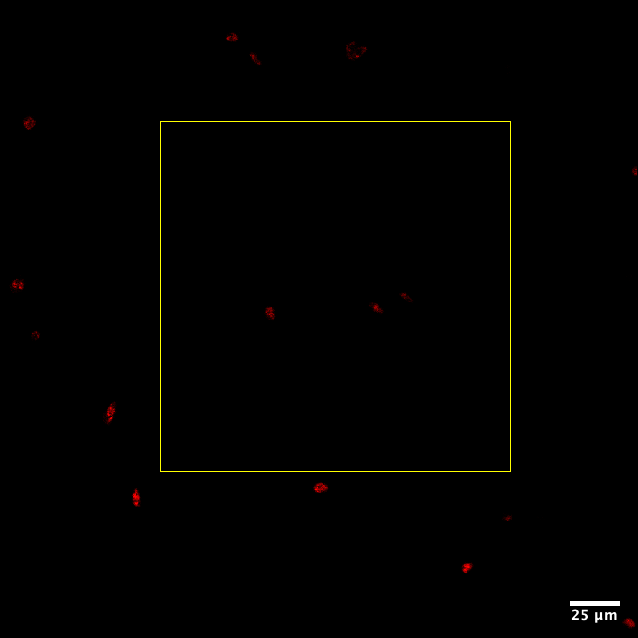

Supplement: Supplementary file 6 — Source data Fig. 3 [file 44319_2024_305_MOESM6_ESM.zip › Figure 3/3F/Mpp7 cKO/Figure 3F. Representative images of CARM1 expression in Mpp7cKO MuSCs_CARM1.tif]

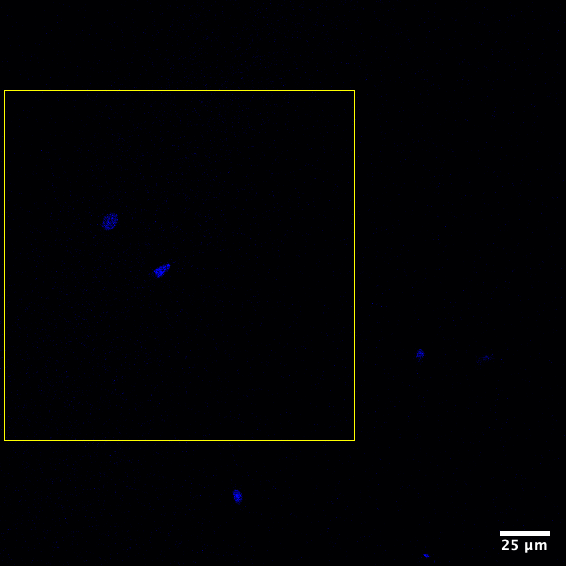

Supplement: Supplementary file 6 — Source data Fig. 3 [file 44319_2024_305_MOESM6_ESM.zip › Figure 3/3F/Amot cKO/Amot cKO MuSCs_DAPI.tif]

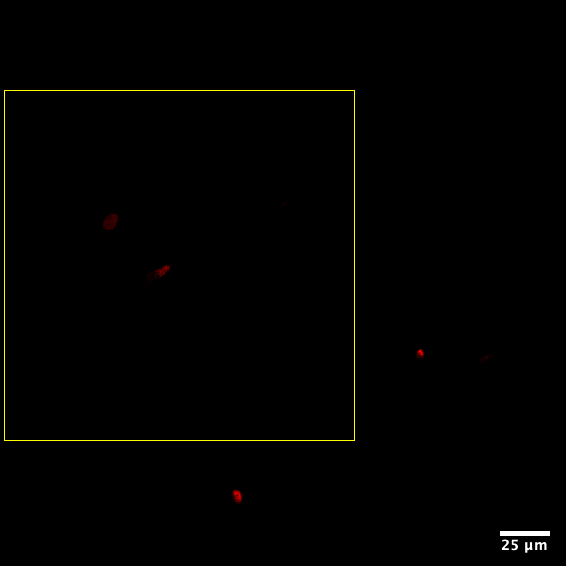

Supplement: Supplementary file 6 — Source data Fig. 3 [file 44319_2024_305_MOESM6_ESM.zip › Figure 3/3F/Amot cKO/Amot cKO MuSCs_CARM1.tif]

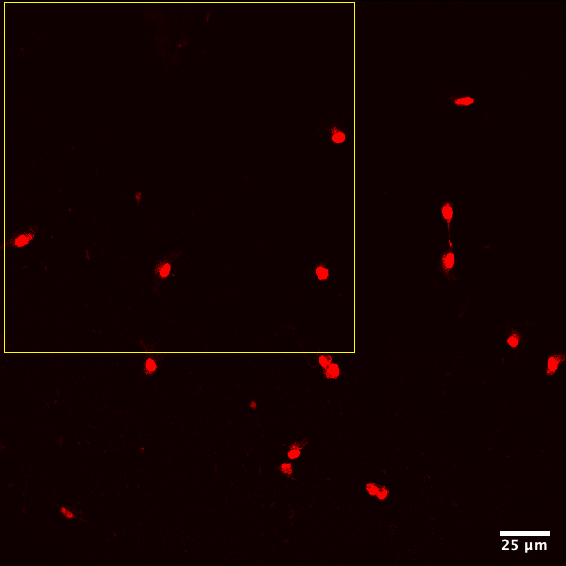

Supplement: Supplementary file 6 — Source data Fig. 3 [file 44319_2024_305_MOESM6_ESM.zip › Figure 3/3F/Con /Control MuSCs_CARM1.tif]

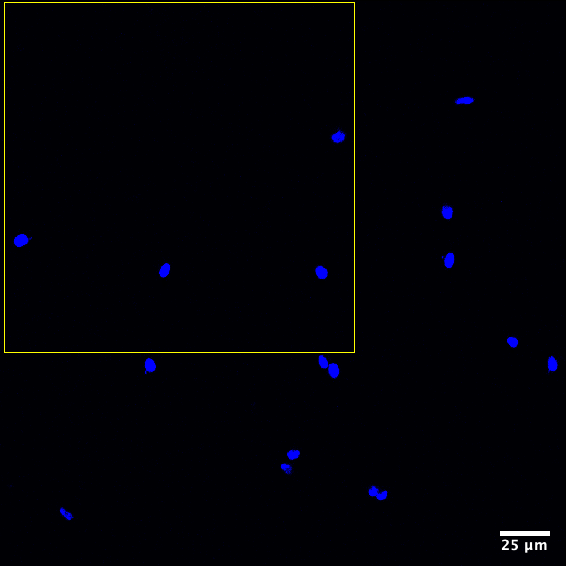

Supplement: Supplementary file 6 — Source data Fig. 3 [file 44319_2024_305_MOESM6_ESM.zip › Figure 3/3F/Con /Control MuSCs_DAPI.tif]

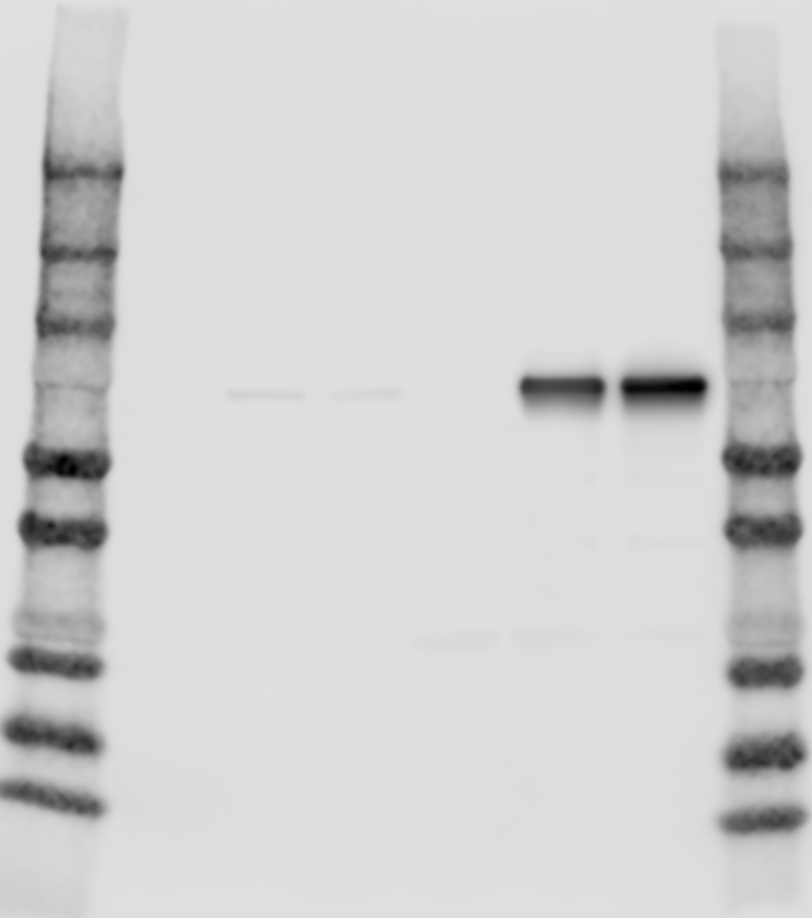

Supplement: Supplementary file 7 — Source data Fig. 4 [file 44319_2024_305_MOESM7_ESM.zip › Figure 4 /4E/blotting Flag-M7.tif]

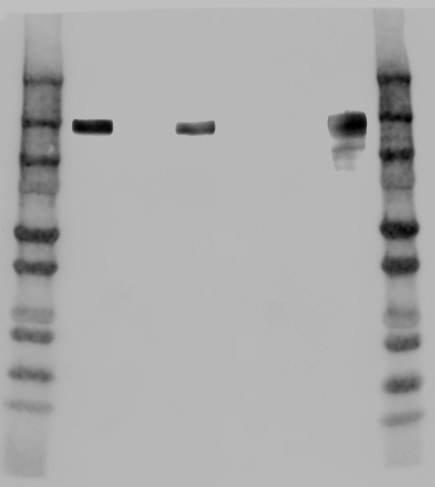

Supplement: Supplementary file 7 — Source data Fig. 4 [file 44319_2024_305_MOESM7_ESM.zip › Figure 4 /4E/blotting Ha-Amot.tif]

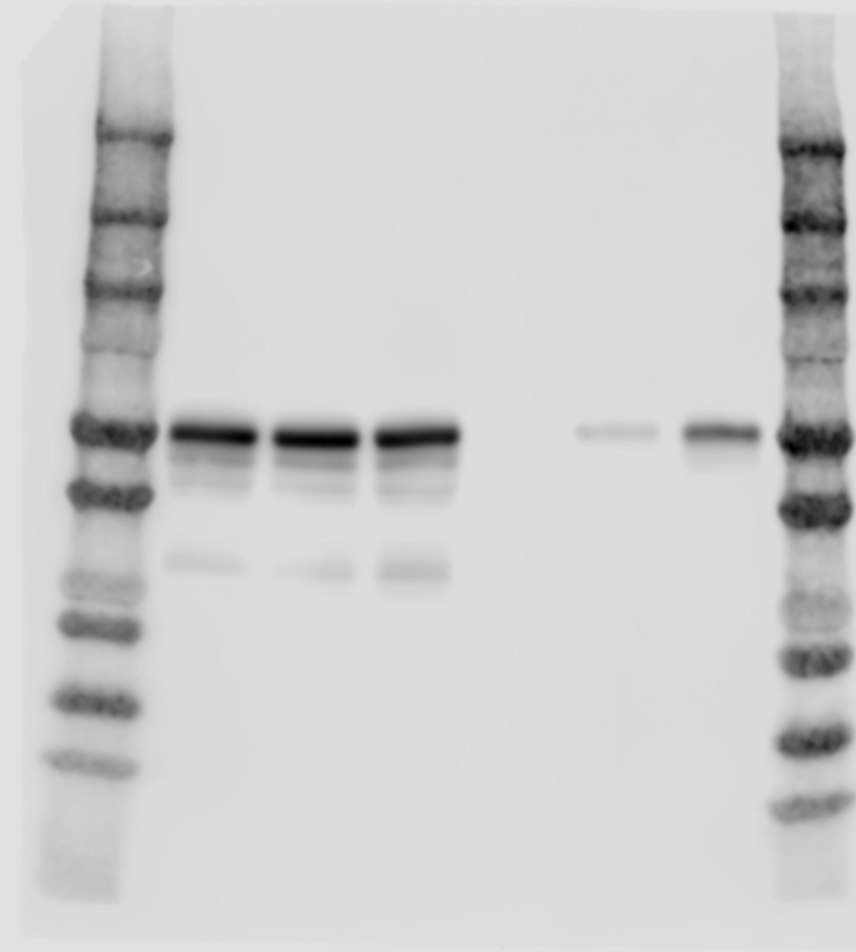

Supplement: Supplementary file 7 — Source data Fig. 4 [file 44319_2024_305_MOESM7_ESM.zip › Figure 4 /4E/blotting V5-Taz.tif]

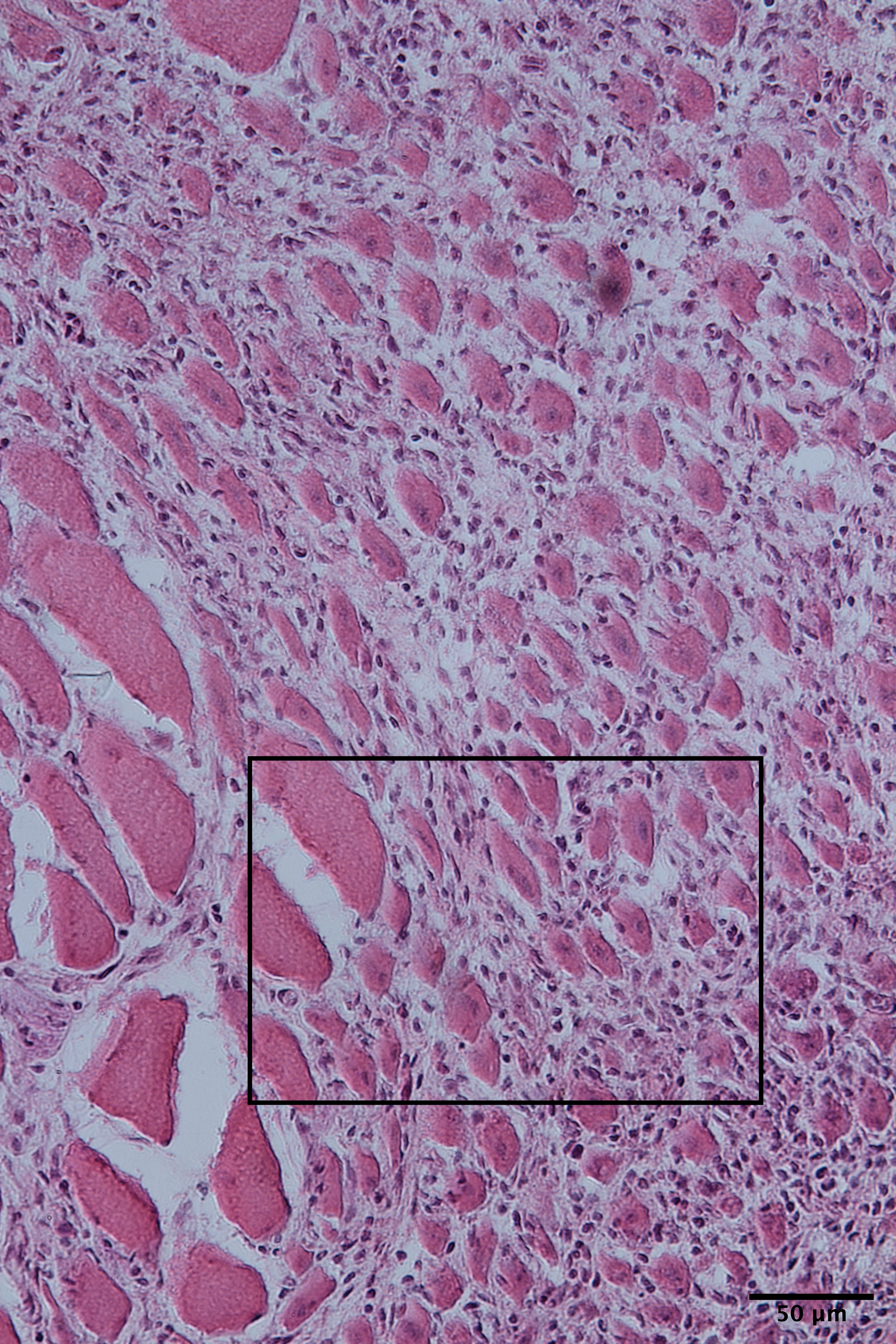

Supplement: Supplementary file 7 — Source data Fig. 4 [file 44319_2024_305_MOESM7_ESM.zip › Figure 4 /4A/4A Yap 5 dpi.jpg]

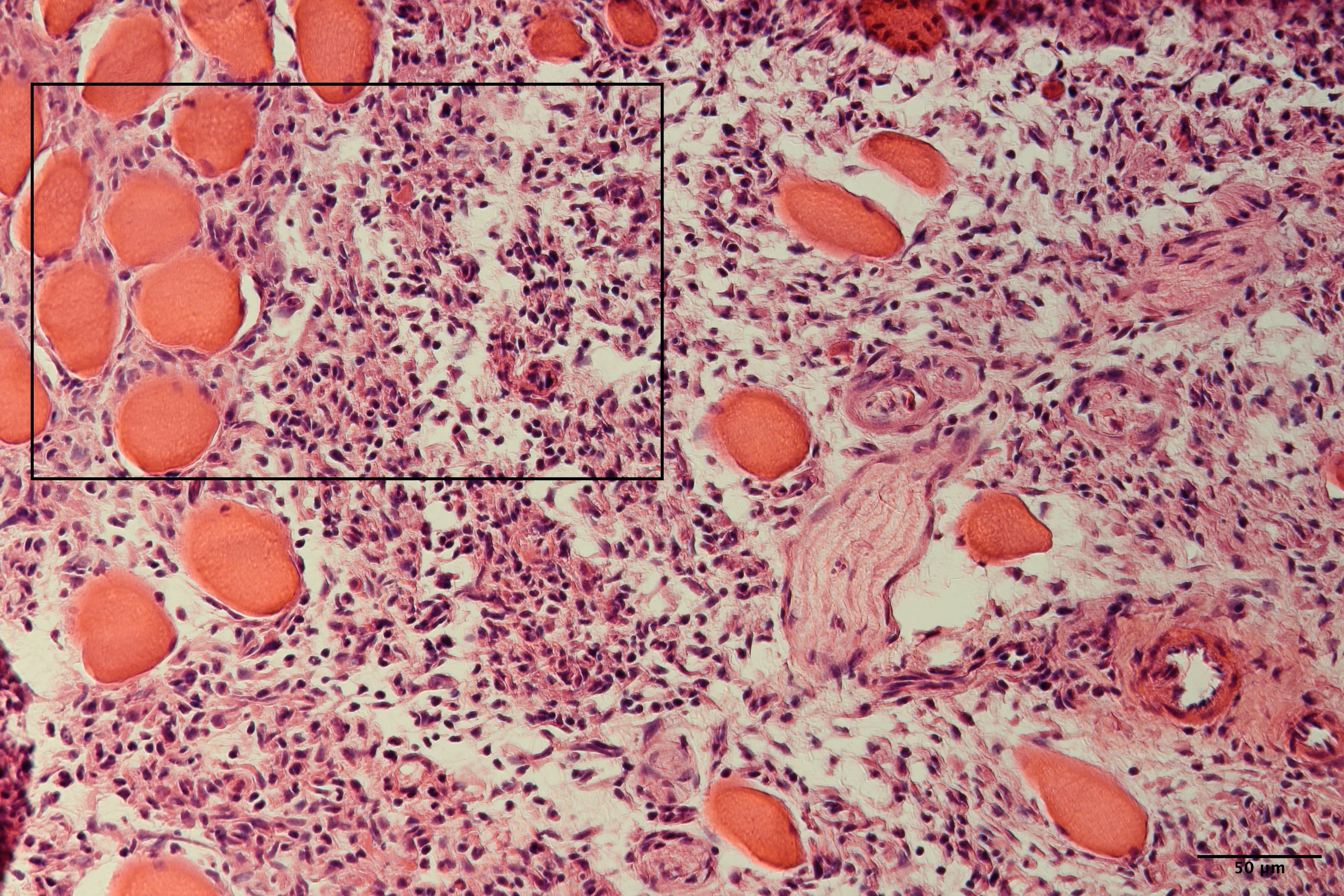

Supplement: Supplementary file 7 — Source data Fig. 4 [file 44319_2024_305_MOESM7_ESM.zip › Figure 4 /4A/2A YapTaz 2cKO.jpg]

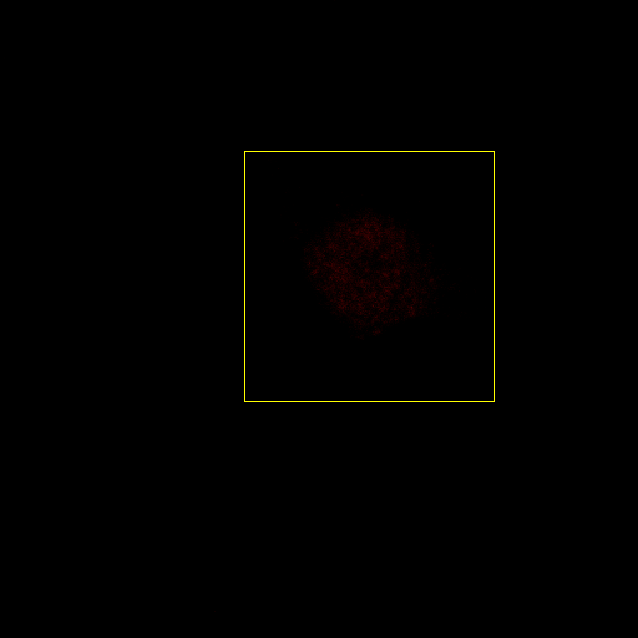

Supplement: Supplementary file 7 — Source data Fig. 4 [file 44319_2024_305_MOESM7_ESM.zip › Figure 4 /4C/Mpp7 cKO/Mpp7 cKO_TAZ 5X.tif]

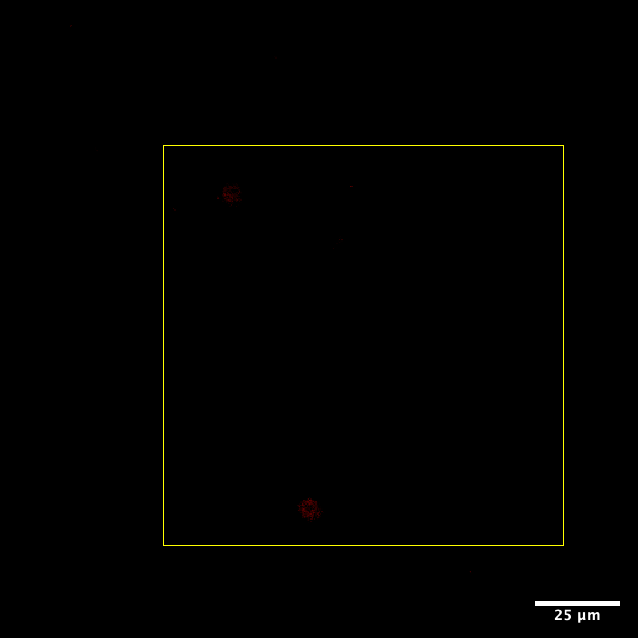

Supplement: Supplementary file 7 — Source data Fig. 4 [file 44319_2024_305_MOESM7_ESM.zip › Figure 4 /4C/Mpp7 cKO/Mpp7 cKO_TAZ .tif]

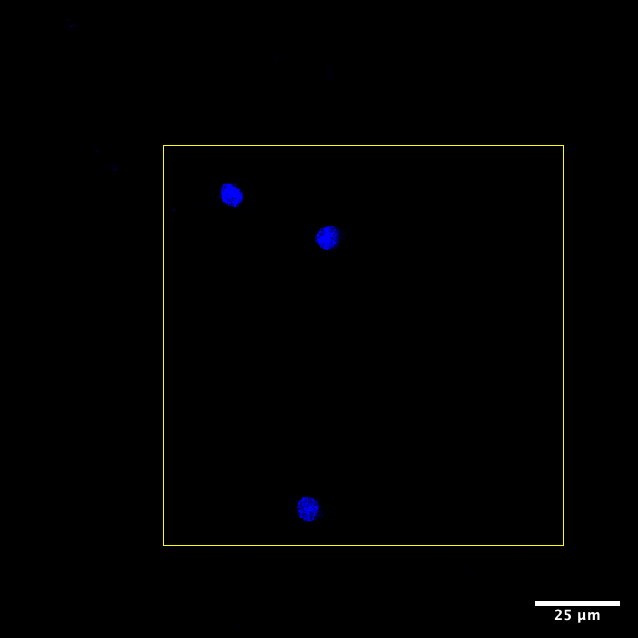

Supplement: Supplementary file 7 — Source data Fig. 4 [file 44319_2024_305_MOESM7_ESM.zip › Figure 4 /4C/Mpp7 cKO/Mpp7 cKO_DAPI.tif]

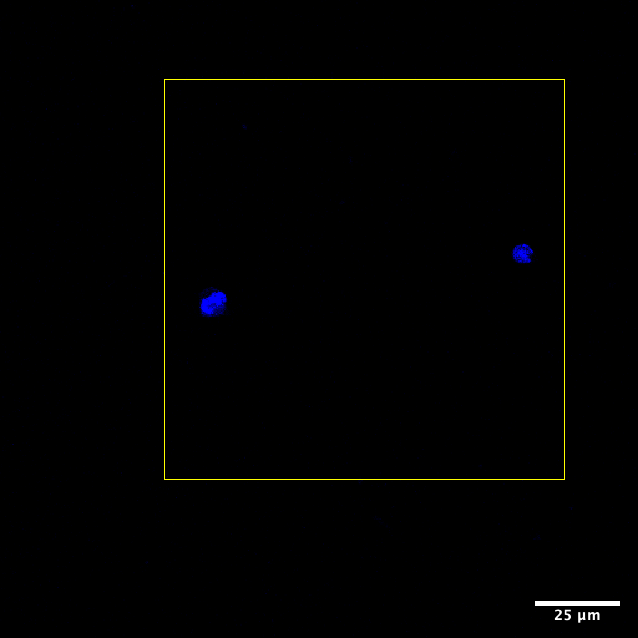

Supplement: Supplementary file 7 — Source data Fig. 4 [file 44319_2024_305_MOESM7_ESM.zip › Figure 4 /4C/Amot cKO/Amot cKO MuSCs_DAPI.tif]

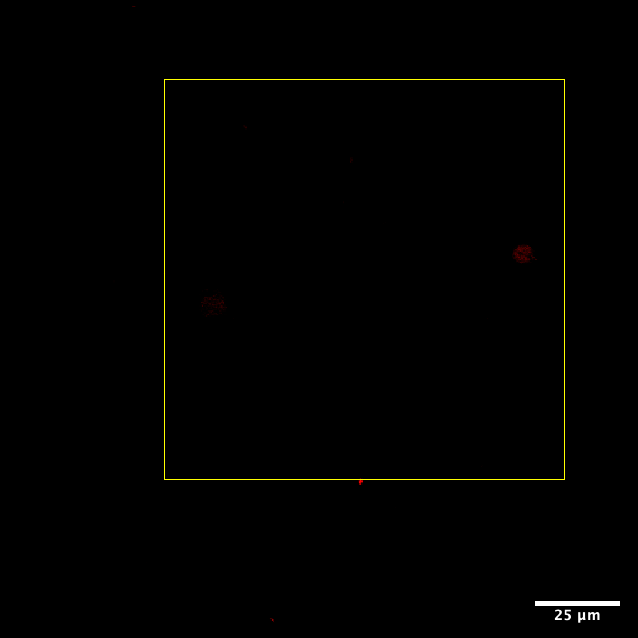

Supplement: Supplementary file 7 — Source data Fig. 4 [file 44319_2024_305_MOESM7_ESM.zip › Figure 4 /4C/Amot cKO/Amot cKO MuSCs_TAZ.tif]

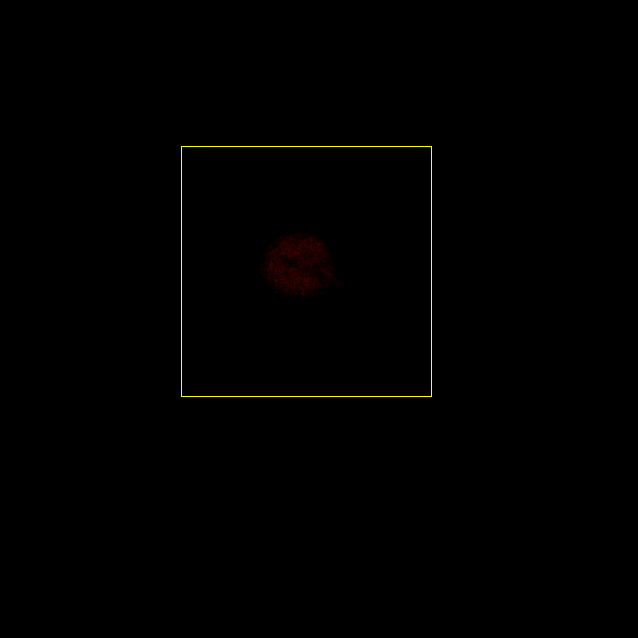

Supplement: Supplementary file 7 — Source data Fig. 4 [file 44319_2024_305_MOESM7_ESM.zip › Figure 4 /4C/Amot cKO/Amot cKO MuSCs_TAZ 5X.tif]

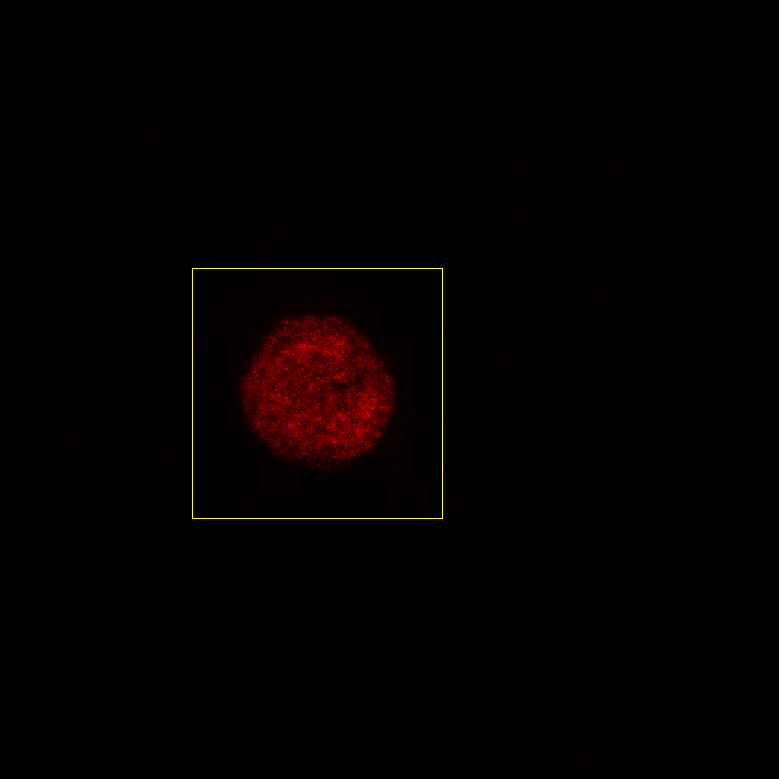

Supplement: Supplementary file 7 — Source data Fig. 4 [file 44319_2024_305_MOESM7_ESM.zip › Figure 4 /4C/Control/Con_TAZ_5X.tif]

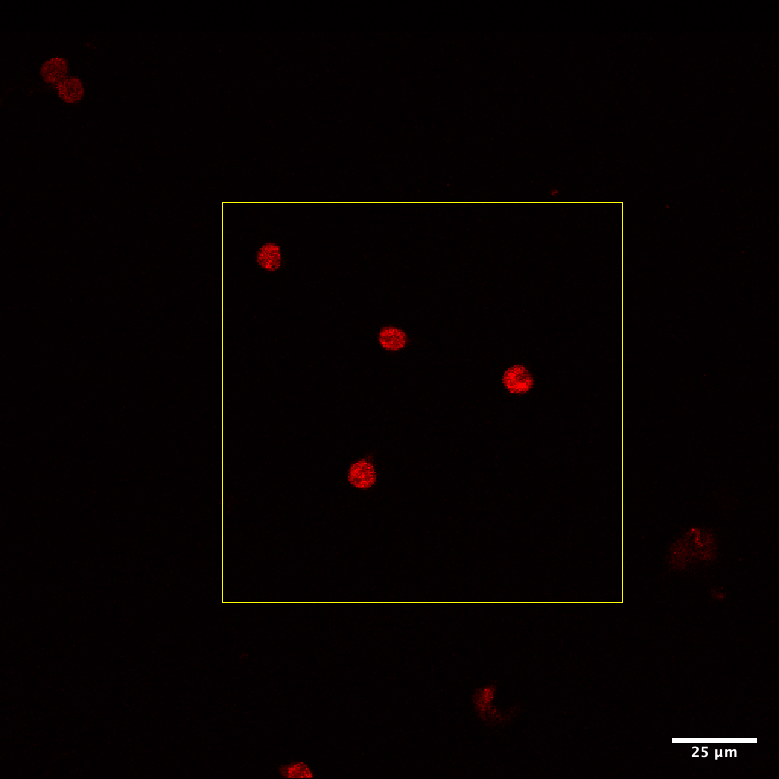

Supplement: Supplementary file 7 — Source data Fig. 4 [file 44319_2024_305_MOESM7_ESM.zip › Figure 4 /4C/Control/Con_TAZ.tif]

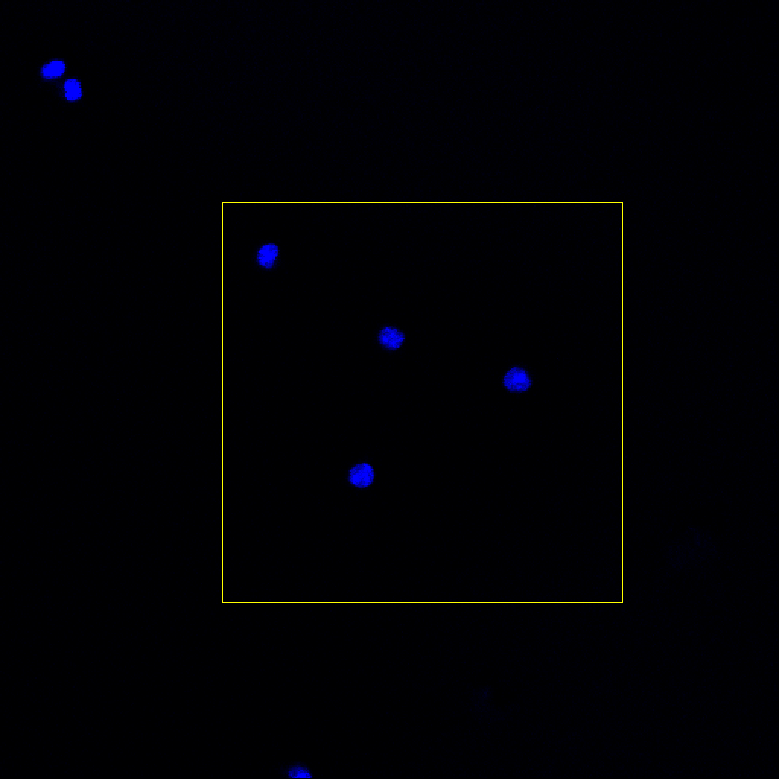

Supplement: Supplementary file 7 — Source data Fig. 4 [file 44319_2024_305_MOESM7_ESM.zip › Figure 4 /4C/Control/Con_DAPI.tif]

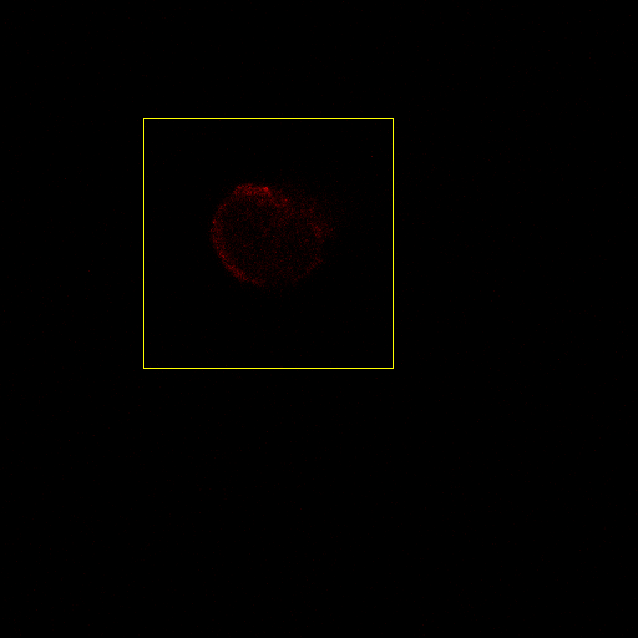

Supplement: Supplementary file 7 — Source data Fig. 4 [file 44319_2024_305_MOESM7_ESM.zip › Figure 4 /4D/Mpp7 cKO/Mpp7 cKO_YAP 5X.tif]

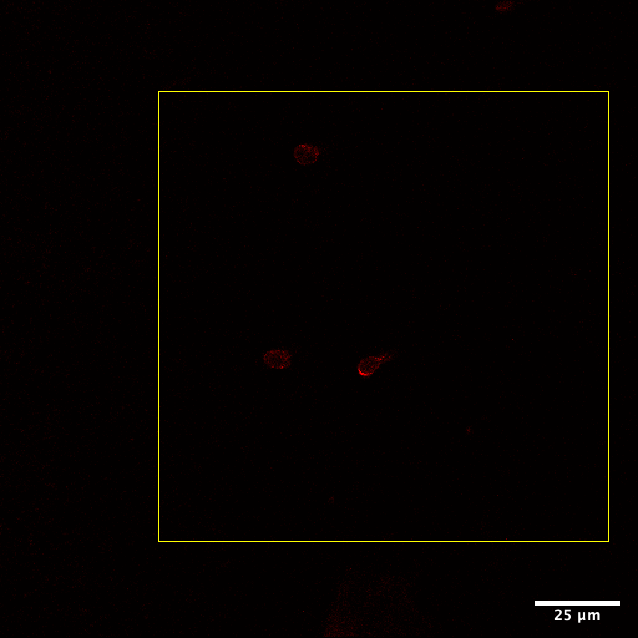

Supplement: Supplementary file 7 — Source data Fig. 4 [file 44319_2024_305_MOESM7_ESM.zip › Figure 4 /4D/Mpp7 cKO/Mpp7 cKO_YAP.tif]

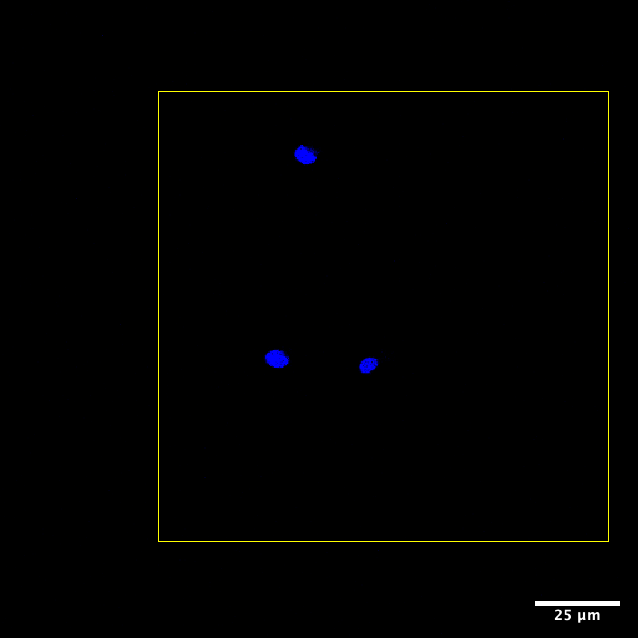

Supplement: Supplementary file 7 — Source data Fig. 4 [file 44319_2024_305_MOESM7_ESM.zip › Figure 4 /4D/Mpp7 cKO/Mpp7 cKO_DAPI.tif]

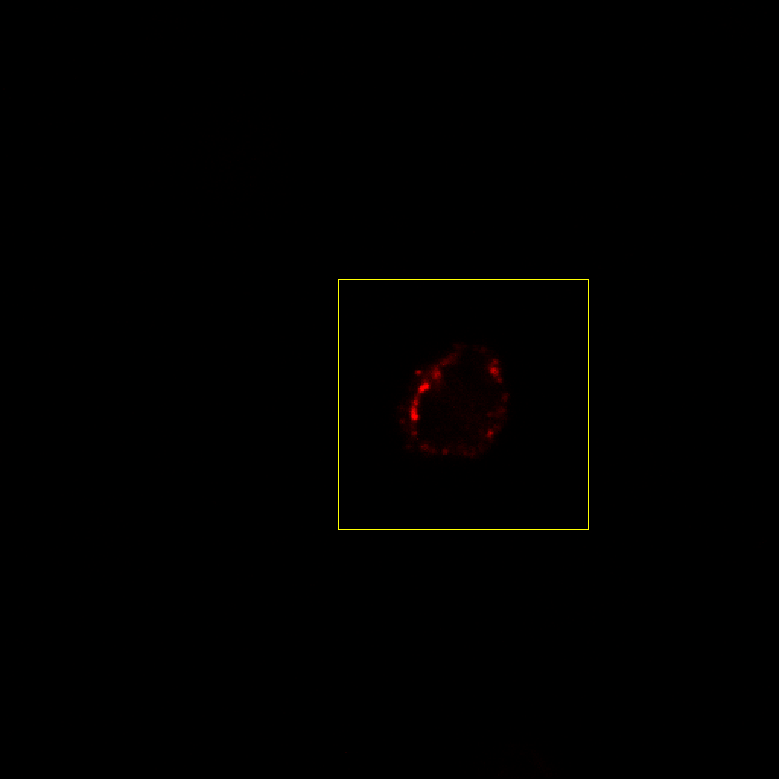

Supplement: Supplementary file 7 — Source data Fig. 4 [file 44319_2024_305_MOESM7_ESM.zip › Figure 4 /4D/Amot cKO/Amot cKO_YAP 5X.tif]

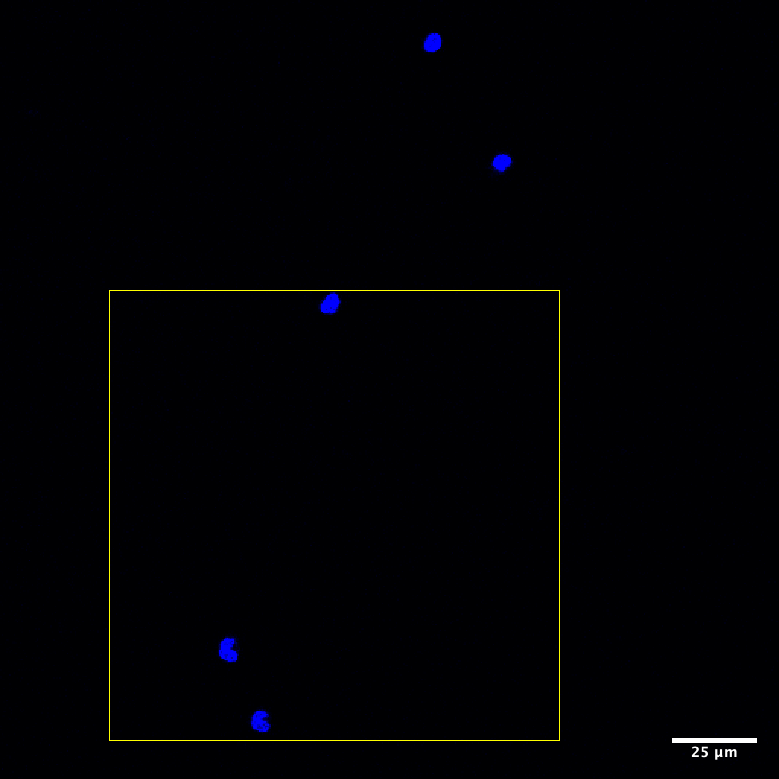

Supplement: Supplementary file 7 — Source data Fig. 4 [file 44319_2024_305_MOESM7_ESM.zip › Figure 4 /4D/Amot cKO/Amot cKO_DAPI.tif]

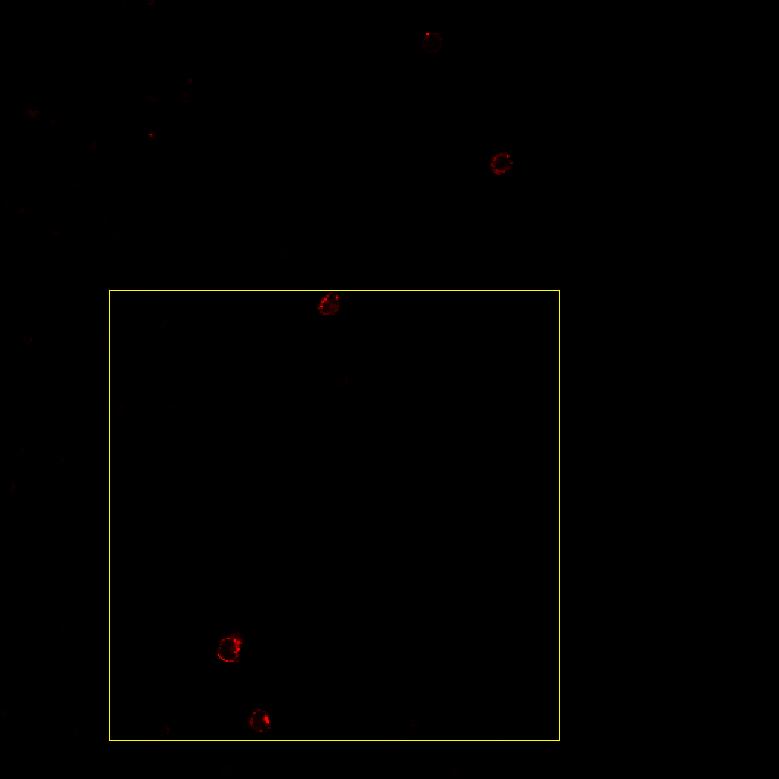

Supplement: Supplementary file 7 — Source data Fig. 4 [file 44319_2024_305_MOESM7_ESM.zip › Figure 4 /4D/Amot cKO/Amot cKO_YAP.tif]

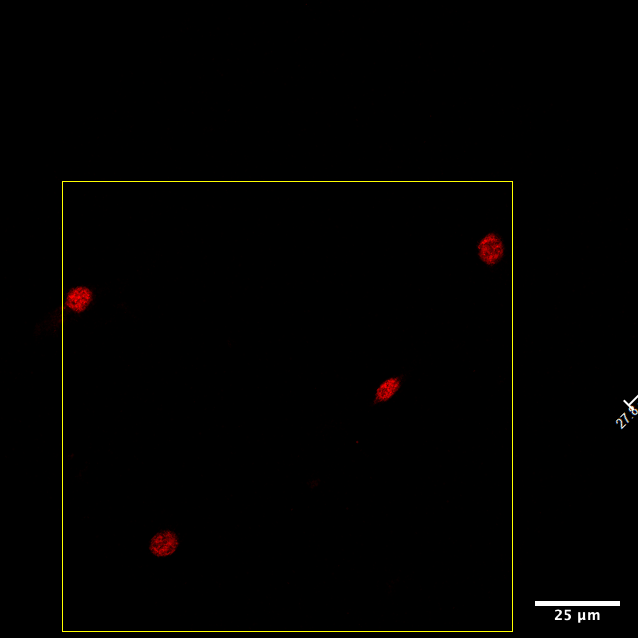

Supplement: Supplementary file 7 — Source data Fig. 4 [file 44319_2024_305_MOESM7_ESM.zip › Figure 4 /4D/Con/YAP.tif]

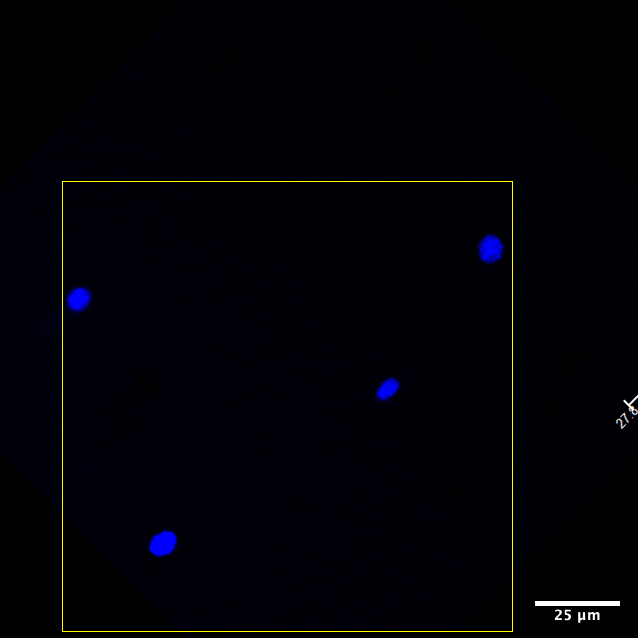

Supplement: Supplementary file 7 — Source data Fig. 4 [file 44319_2024_305_MOESM7_ESM.zip › Figure 4 /4D/Con/DAPI.tif]

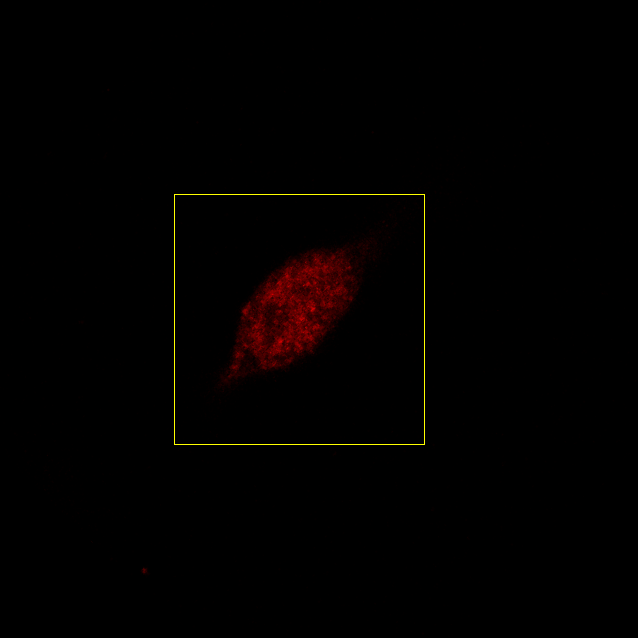

Supplement: Supplementary file 7 — Source data Fig. 4 [file 44319_2024_305_MOESM7_ESM.zip › Figure 4 /4D/Con/YAP 5X.tif]

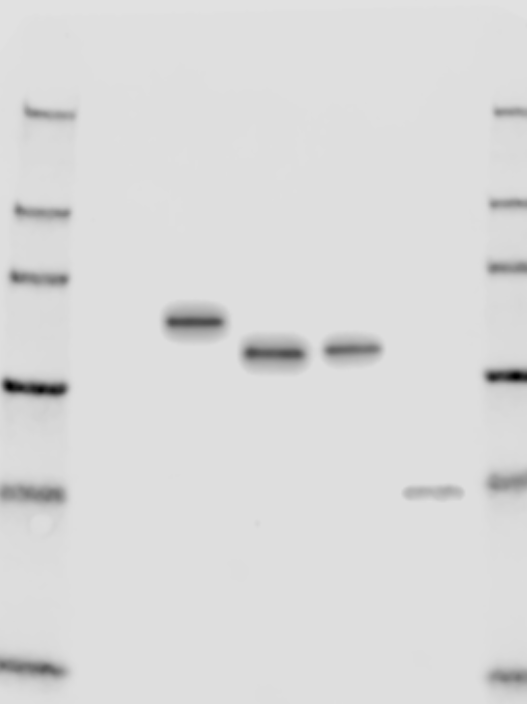

Supplement: Supplementary file 8 — Source data Fig. 5 [file 44319_2024_305_MOESM8_ESM.zip › Figure 5/5A/Input Flag-Mpp7 mutants.tif]

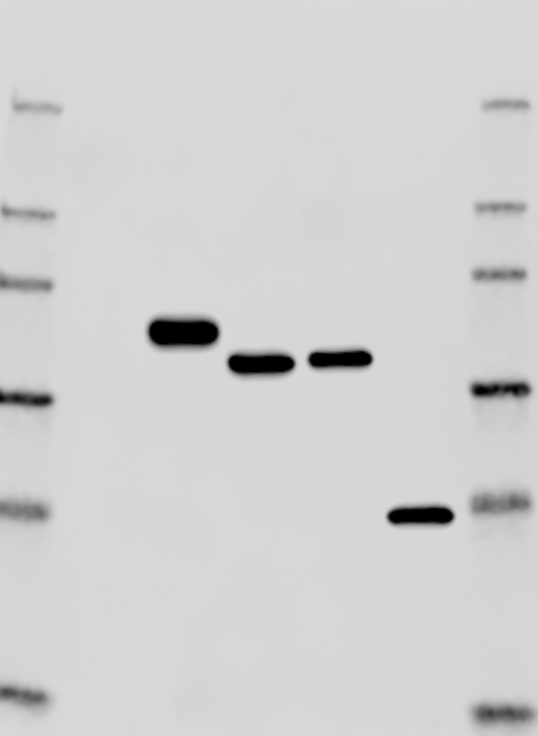

Supplement: Supplementary file 8 — Source data Fig. 5 [file 44319_2024_305_MOESM8_ESM.zip › Figure 5/5A/IP Flag-Mpp7.tif]

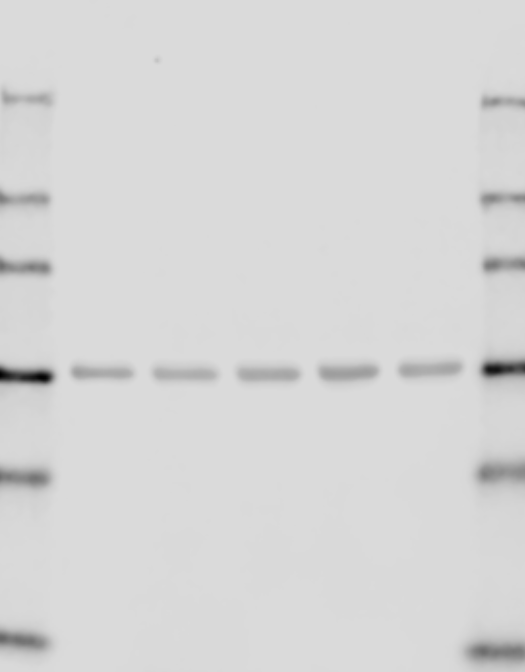

Supplement: Supplementary file 8 — Source data Fig. 5 [file 44319_2024_305_MOESM8_ESM.zip › Figure 5/5A/Input V5-TAZ.tif]

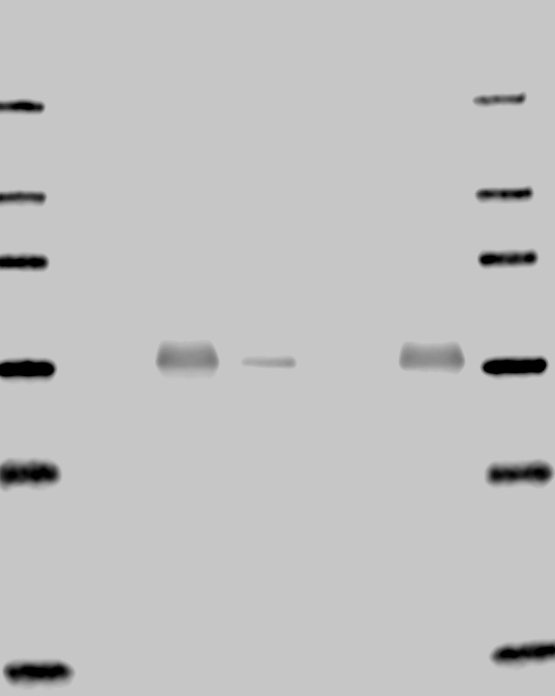

Supplement: Supplementary file 8 — Source data Fig. 5 [file 44319_2024_305_MOESM8_ESM.zip › Figure 5/5A/co-IPed TAZ.tif]

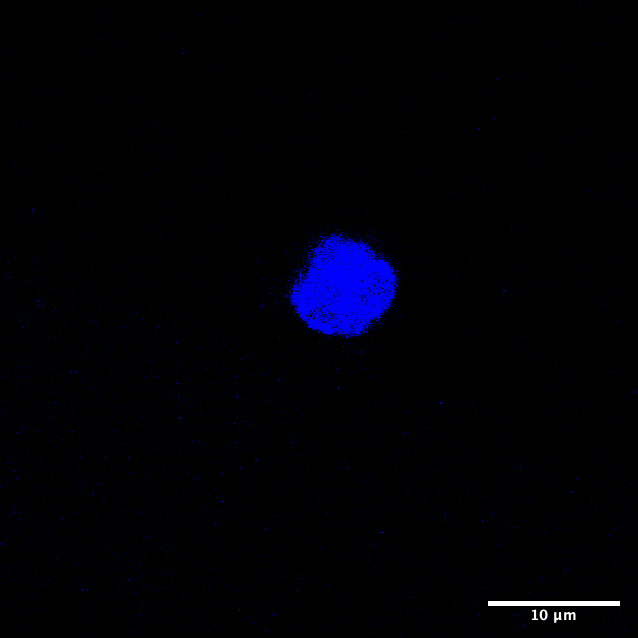

Supplement: Supplementary file 9 — Source data Fig. 6 [file 44319_2024_305_MOESM9_ESM.zip › Figure 6/6F/AMOT_3PY_DAPI .tif]

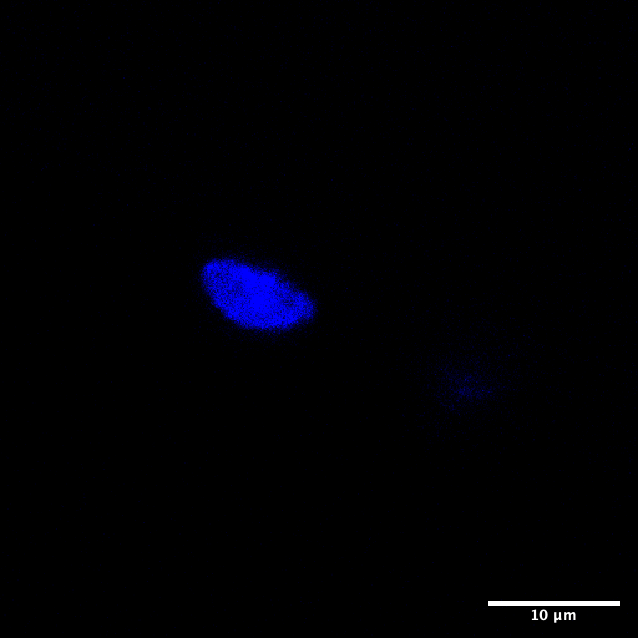

Supplement: Supplementary file 9 — Source data Fig. 6 [file 44319_2024_305_MOESM9_ESM.zip › Figure 6/6F/AMOT_S175A_DAPI.tif]

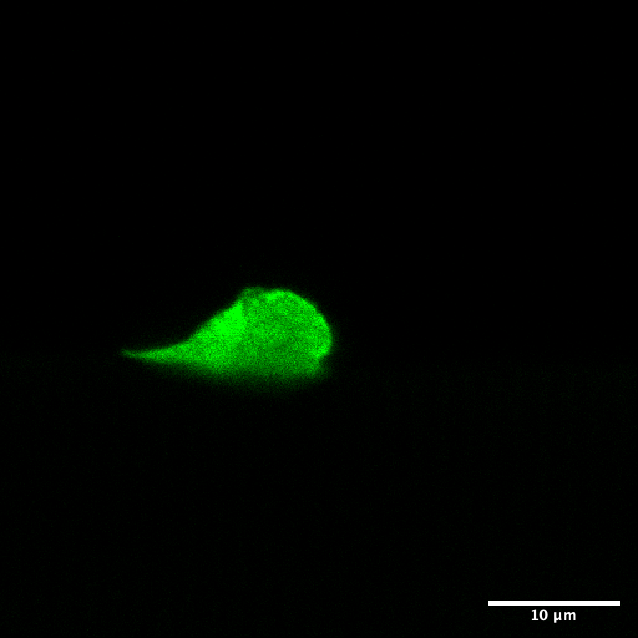

Supplement: Supplementary file 9 — Source data Fig. 6 [file 44319_2024_305_MOESM9_ESM.zip › Figure 6/6F/AMOT_S175E_YFP.tif]

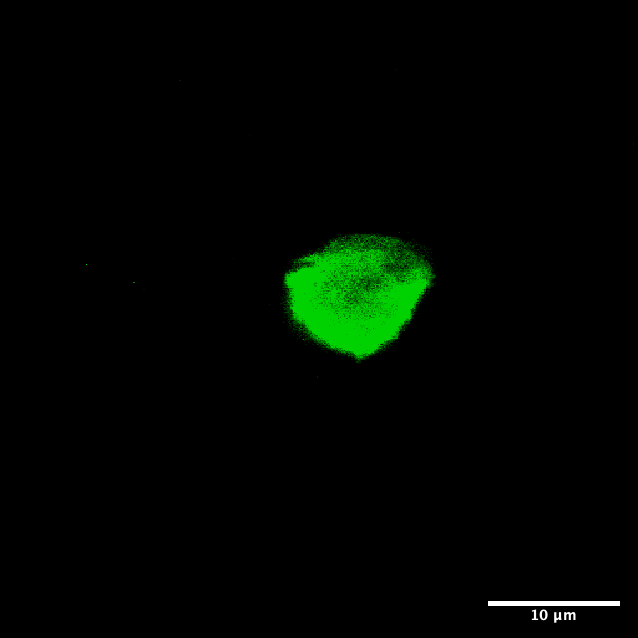

Supplement: Supplementary file 9 — Source data Fig. 6 [file 44319_2024_305_MOESM9_ESM.zip › Figure 6/6F/AMOT_WT_YFP.tif]

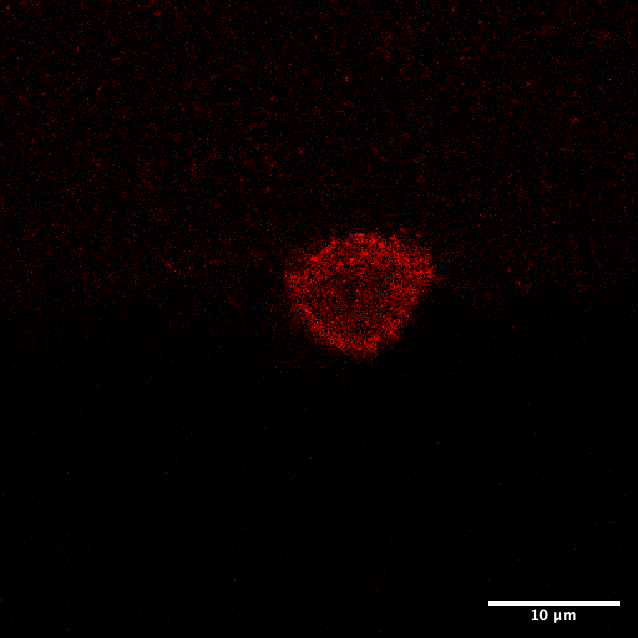

Supplement: Supplementary file 9 — Source data Fig. 6 [file 44319_2024_305_MOESM9_ESM.zip › Figure 6/6F/AMOT_WT_HA_AMOT .tif]

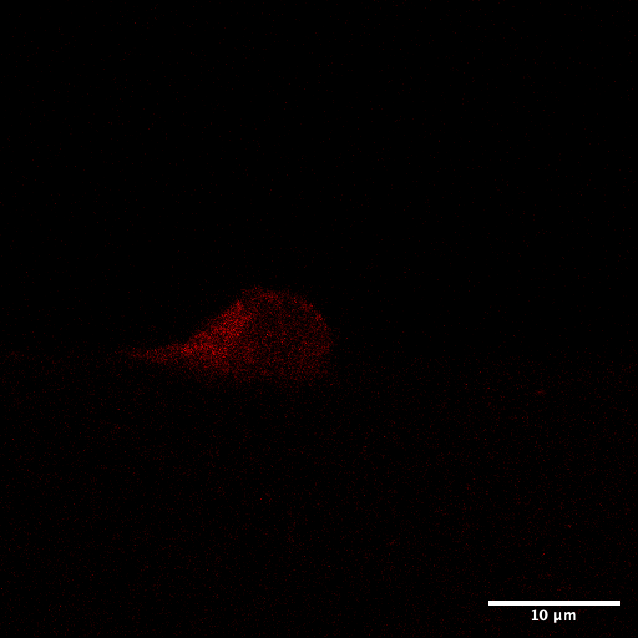

Supplement: Supplementary file 9 — Source data Fig. 6 [file 44319_2024_305_MOESM9_ESM.zip › Figure 6/6F/AMOT_S175E_HA_AMOT.tif]

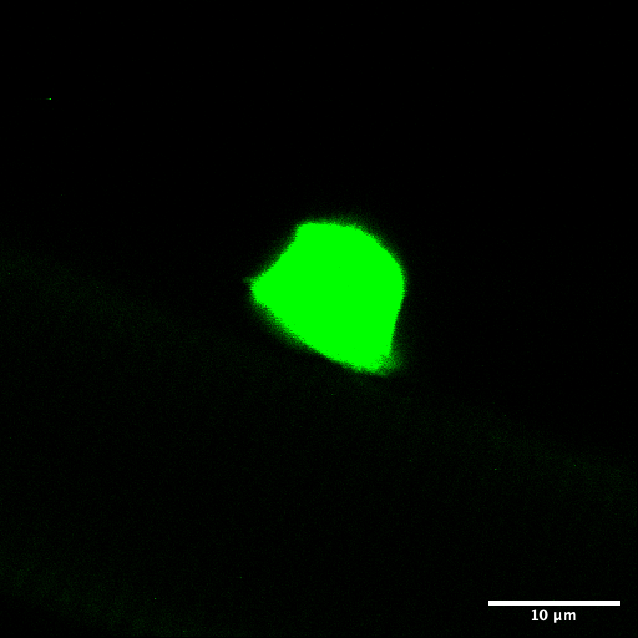

Supplement: Supplementary file 9 — Source data Fig. 6 [file 44319_2024_305_MOESM9_ESM.zip › Figure 6/6F/AMOT_3PY _YFP.tif]

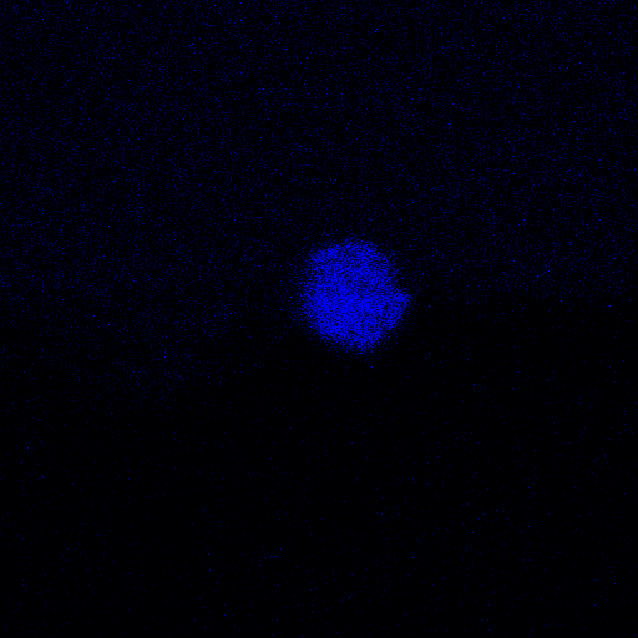

Supplement: Supplementary file 9 — Source data Fig. 6 [file 44319_2024_305_MOESM9_ESM.zip › Figure 6/6F/AMOT_WT_DAPI.tif]

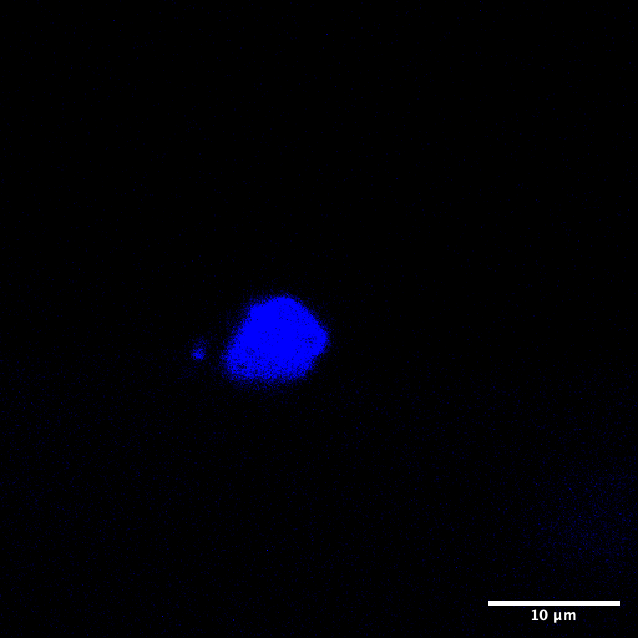

Supplement: Supplementary file 9 — Source data Fig. 6 [file 44319_2024_305_MOESM9_ESM.zip › Figure 6/6F/AMOT_S175E_DAPI .tif]

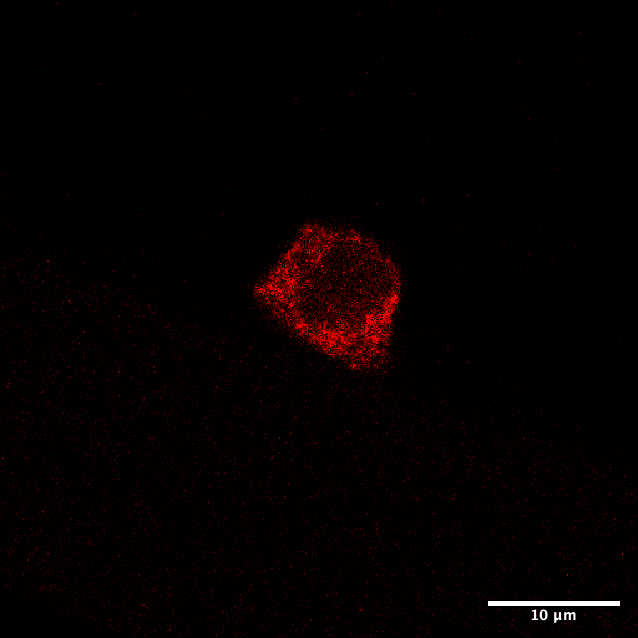

Supplement: Supplementary file 9 — Source data Fig. 6 [file 44319_2024_305_MOESM9_ESM.zip › Figure 6/6F/AMOT_3PY_HA_AMOT .tif]

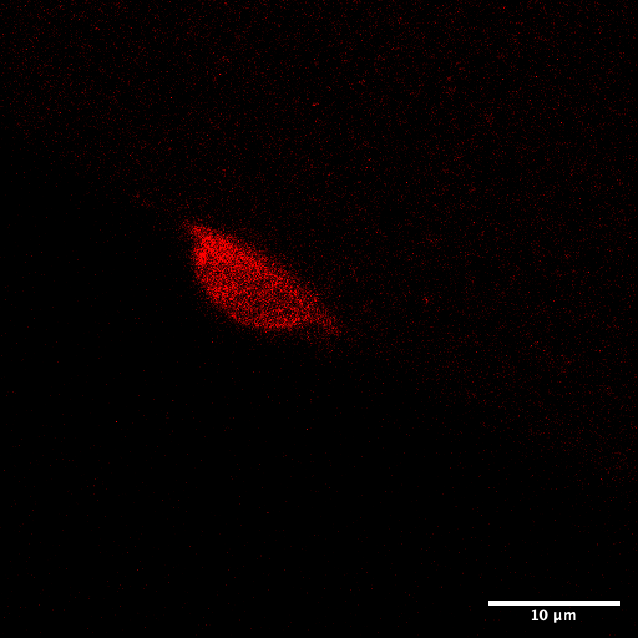

Supplement: Supplementary file 9 — Source data Fig. 6 [file 44319_2024_305_MOESM9_ESM.zip › Figure 6/6F/AMOT_S175A_HA_AMOT.tif]

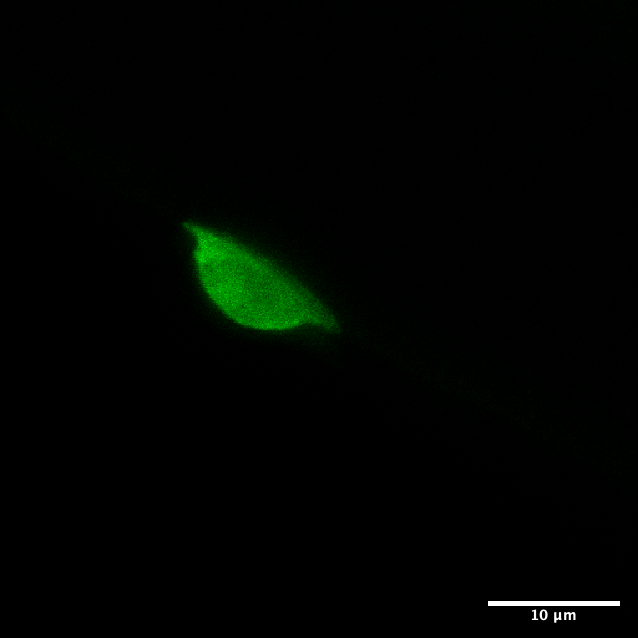

Supplement: Supplementary file 9 — Source data Fig. 6 [file 44319_2024_305_MOESM9_ESM.zip › Figure 6/6F/AMOT_S175A_YFP.tif]

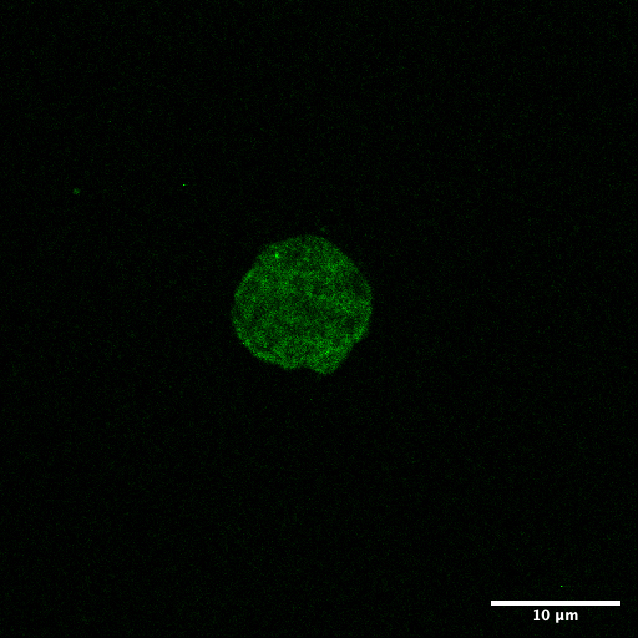

Supplement: Supplementary file 9 — Source data Fig. 6 [file 44319_2024_305_MOESM9_ESM.zip › Figure 6/6B/Jasp_MPP7.tif]

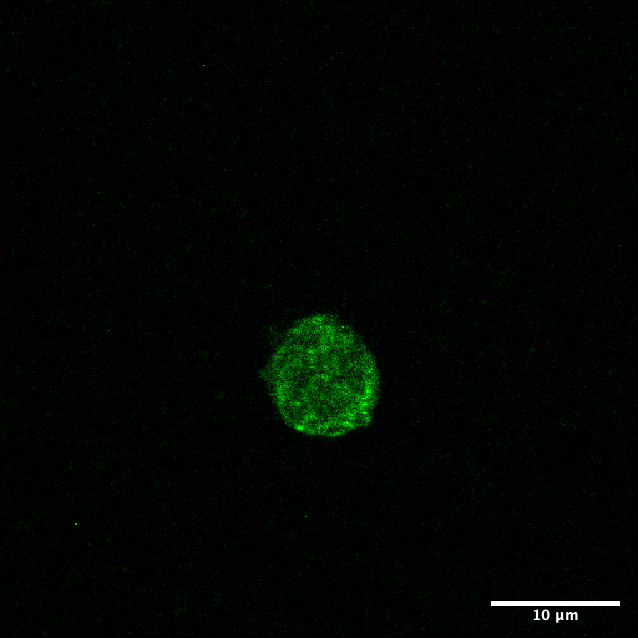

Supplement: Supplementary file 9 — Source data Fig. 6 [file 44319_2024_305_MOESM9_ESM.zip › Figure 6/6B/Nar_MPP7.tif]

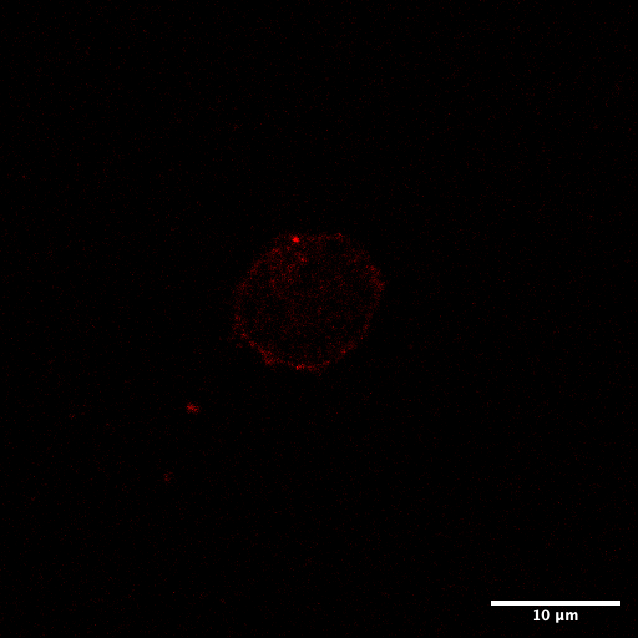

Supplement: Supplementary file 9 — Source data Fig. 6 [file 44319_2024_305_MOESM9_ESM.zip › Figure 6/6B/DMSO_AMOT.tif]

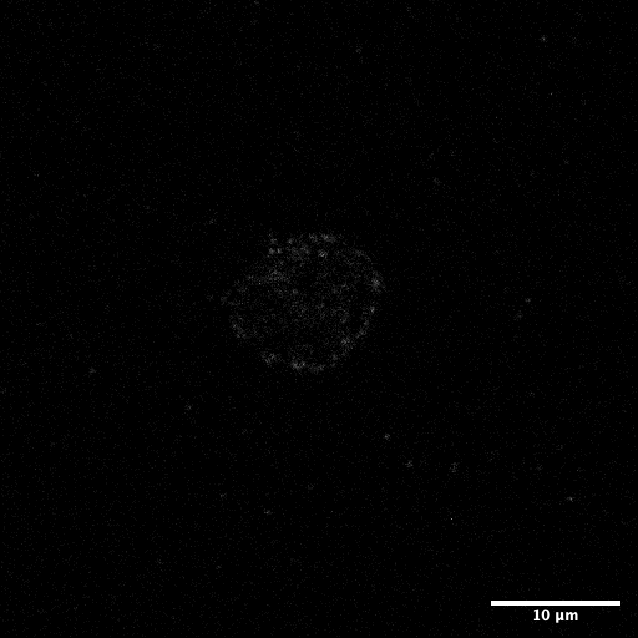

Supplement: Supplementary file 9 — Source data Fig. 6 [file 44319_2024_305_MOESM9_ESM.zip › Figure 6/6B/DMSO_YFP.tif]

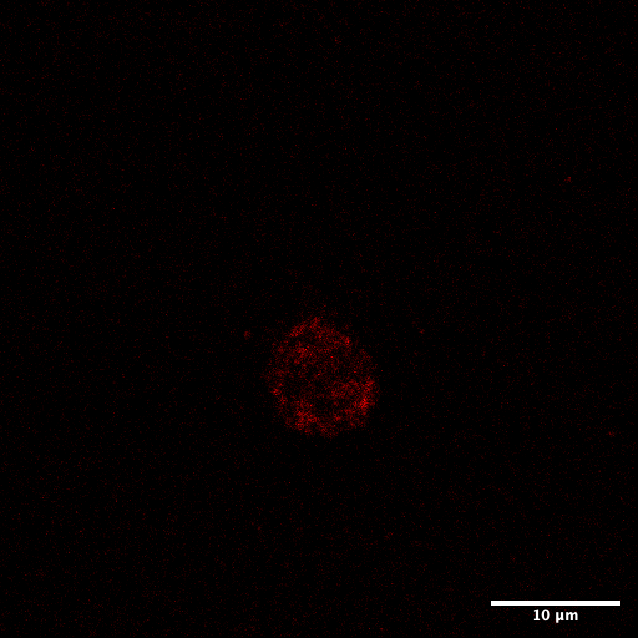

Supplement: Supplementary file 9 — Source data Fig. 6 [file 44319_2024_305_MOESM9_ESM.zip › Figure 6/6B/Nar_AMOT.tif]

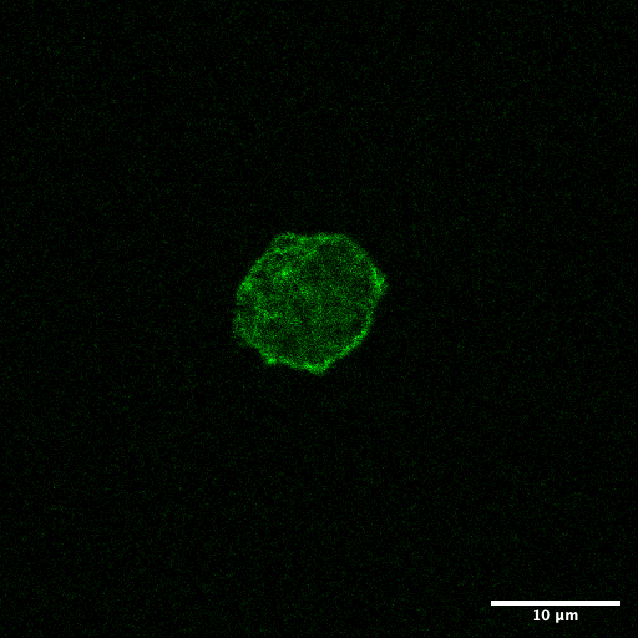

Supplement: Supplementary file 9 — Source data Fig. 6 [file 44319_2024_305_MOESM9_ESM.zip › Figure 6/6B/DMSO_MPP7.tif]

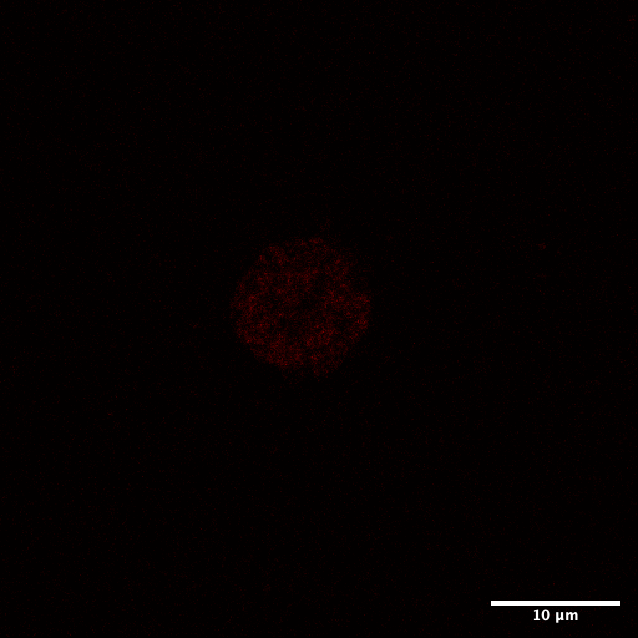

Supplement: Supplementary file 9 — Source data Fig. 6 [file 44319_2024_305_MOESM9_ESM.zip › Figure 6/6B/Jasp_AMOT.tif]

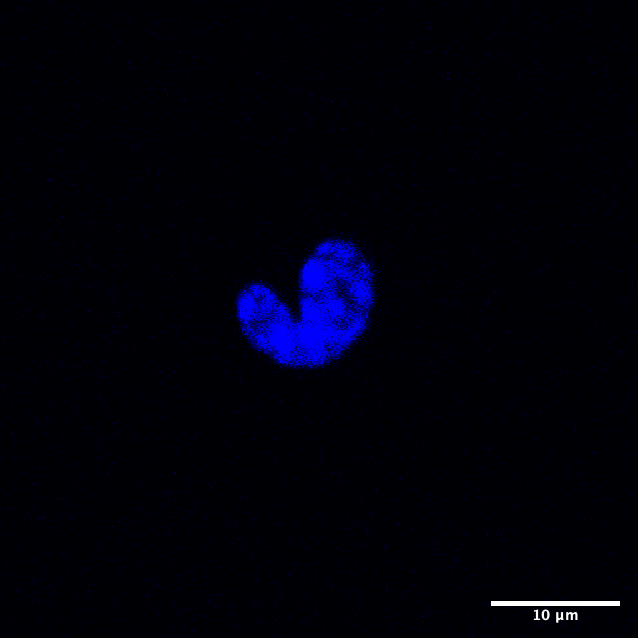

Supplement: Supplementary file 9 — Source data Fig. 6 [file 44319_2024_305_MOESM9_ESM.zip › Figure 6/6B/DMSO_DAPI.tif]

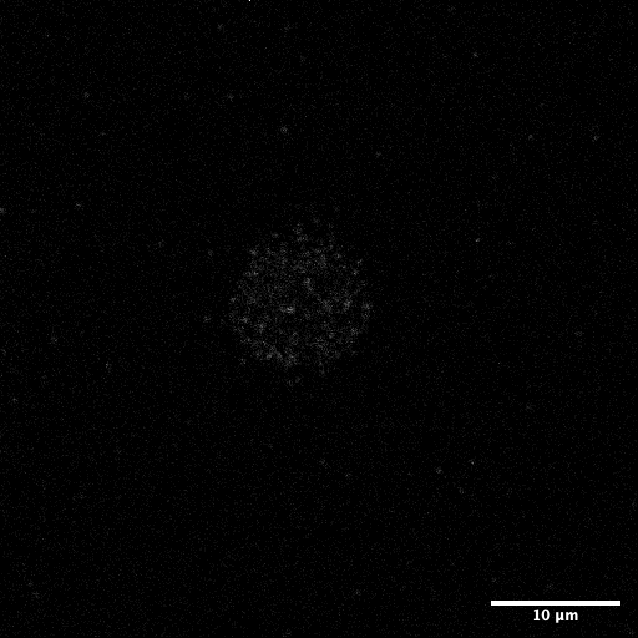

Supplement: Supplementary file 9 — Source data Fig. 6 [file 44319_2024_305_MOESM9_ESM.zip › Figure 6/6B/Jasp_YFP.tif]

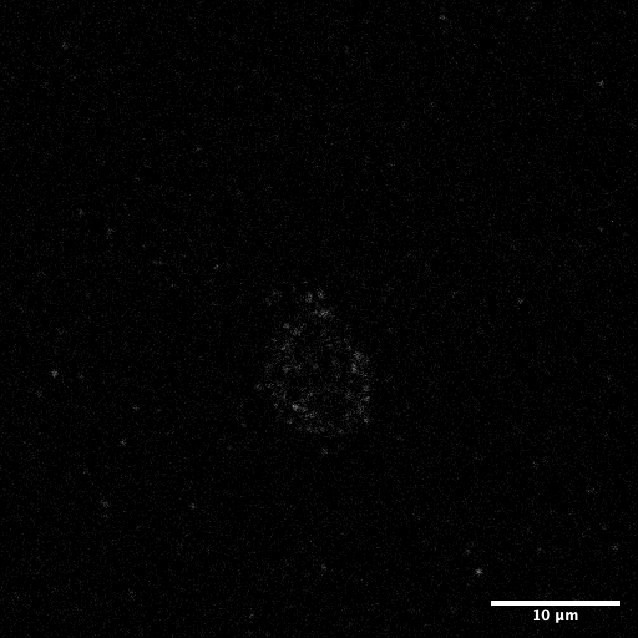

Supplement: Supplementary file 9 — Source data Fig. 6 [file 44319_2024_305_MOESM9_ESM.zip › Figure 6/6B/Nar_YFP.tif]

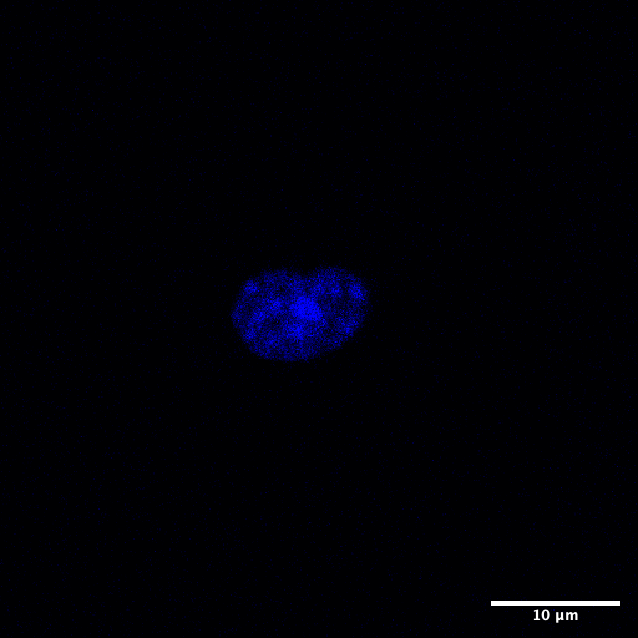

Supplement: Supplementary file 9 — Source data Fig. 6 [file 44319_2024_305_MOESM9_ESM.zip › Figure 6/6B/Jasp_DAPI.tif]

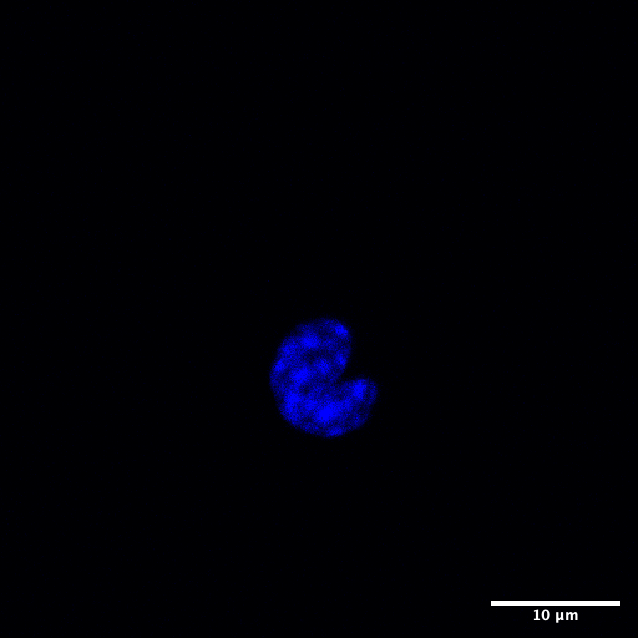

Supplement: Supplementary file 9 — Source data Fig. 6 [file 44319_2024_305_MOESM9_ESM.zip › Figure 6/6B/Nar_DAPI.tif]

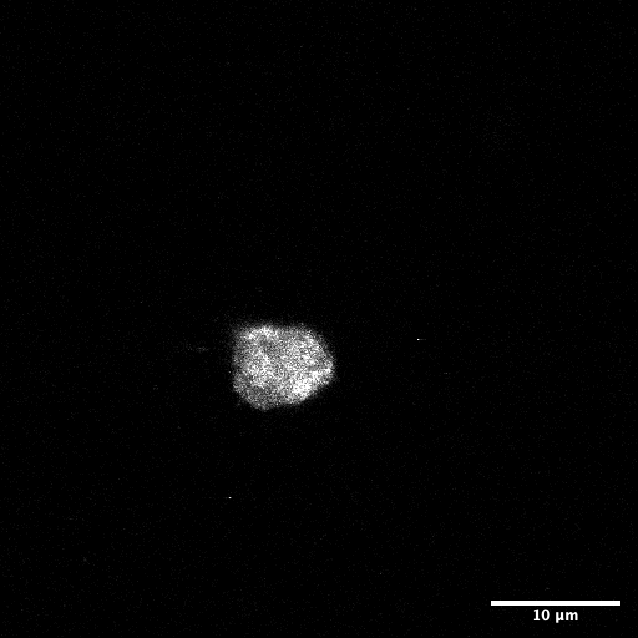

Supplement: Supplementary file 9 — Source data Fig. 6 [file 44319_2024_305_MOESM9_ESM.zip › Figure 6/6D/DMSO_YFP.tif]

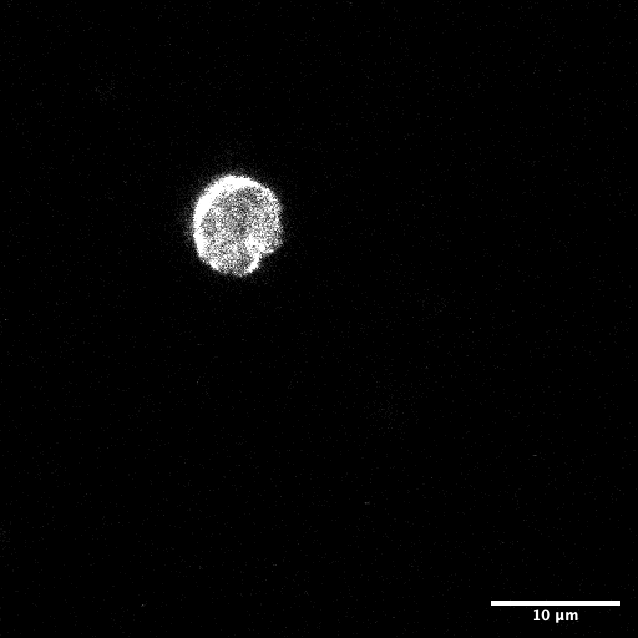

Supplement: Supplementary file 9 — Source data Fig. 6 [file 44319_2024_305_MOESM9_ESM.zip › Figure 6/6D/Cyto_B_YFP.tif]

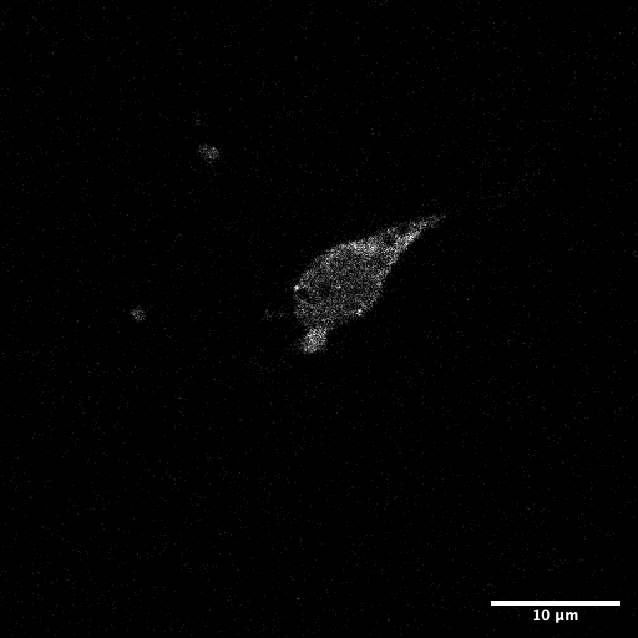

Supplement: Supplementary file 9 — Source data Fig. 6 [file 44319_2024_305_MOESM9_ESM.zip › Figure 6/6D/Bleb_YFP.tif]

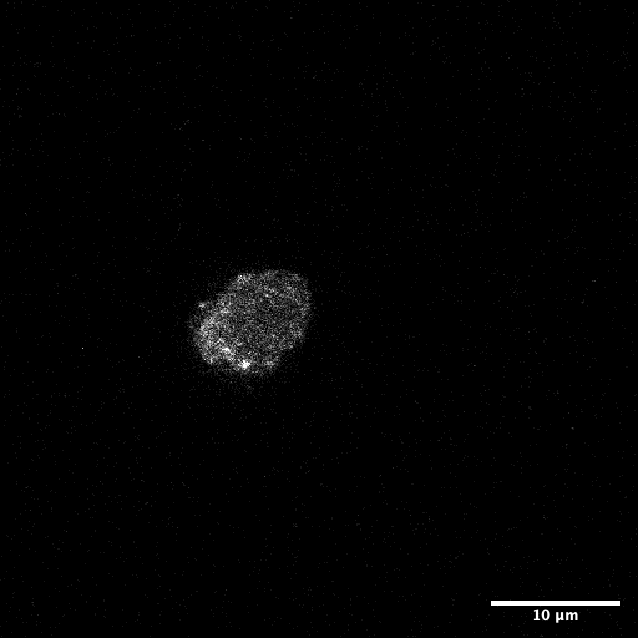

Supplement: Supplementary file 9 — Source data Fig. 6 [file 44319_2024_305_MOESM9_ESM.zip › Figure 6/6D/Y27632_YFP.tif]

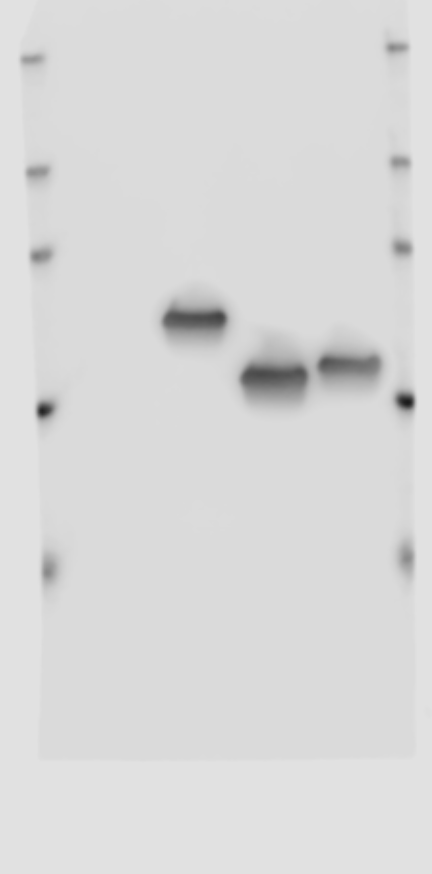

Supplement: Supplementary file 10 — Source data Fig. 7 [file 44319_2024_305_MOESM10_ESM.zip › Figure 7/7F Input FLAG-MPP7 mutants.tif]

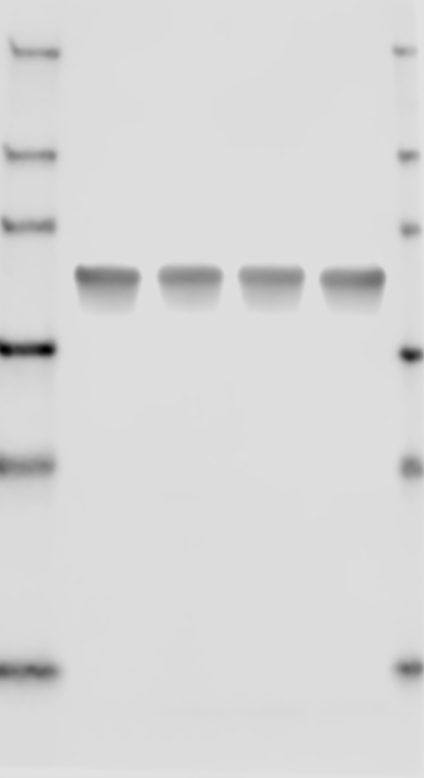

Supplement: Supplementary file 10 — Source data Fig. 7 [file 44319_2024_305_MOESM10_ESM.zip › Figure 7/7F Input HA-YY1.tif]

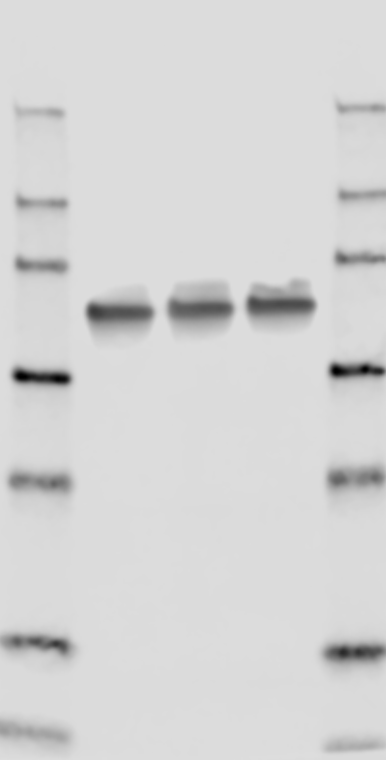

Supplement: Supplementary file 10 — Source data Fig. 7 [file 44319_2024_305_MOESM10_ESM.zip › Figure 7/7E Input HA-YY1.tif]

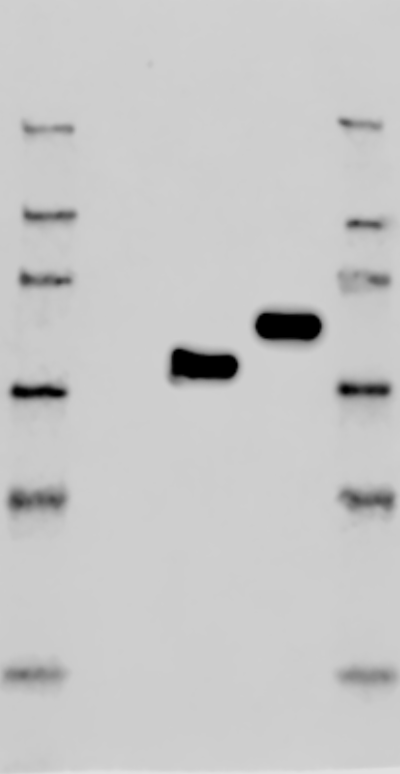

Supplement: Supplementary file 10 — Source data Fig. 7 [file 44319_2024_305_MOESM10_ESM.zip › Figure 7/7E IP FLAG-TAZ.tif]

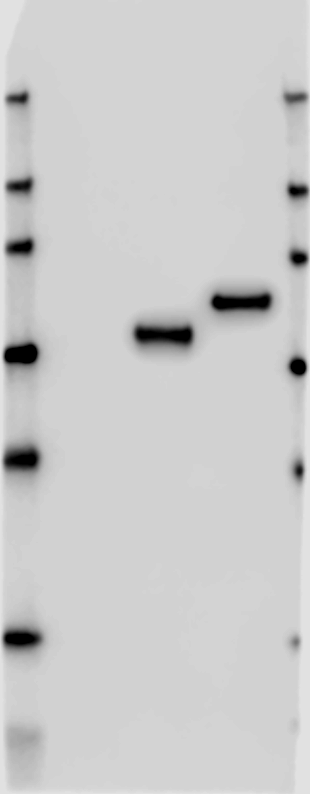

Supplement: Supplementary file 10 — Source data Fig. 7 [file 44319_2024_305_MOESM10_ESM.zip › Figure 7/7E Input Flag-Taz.tif]

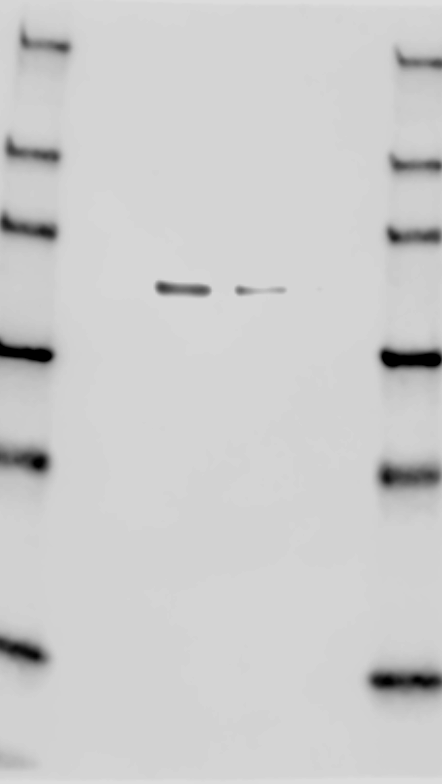

Supplement: Supplementary file 10 — Source data Fig. 7 [file 44319_2024_305_MOESM10_ESM.zip › Figure 7/7F co-IPed HA-YY1.tif]

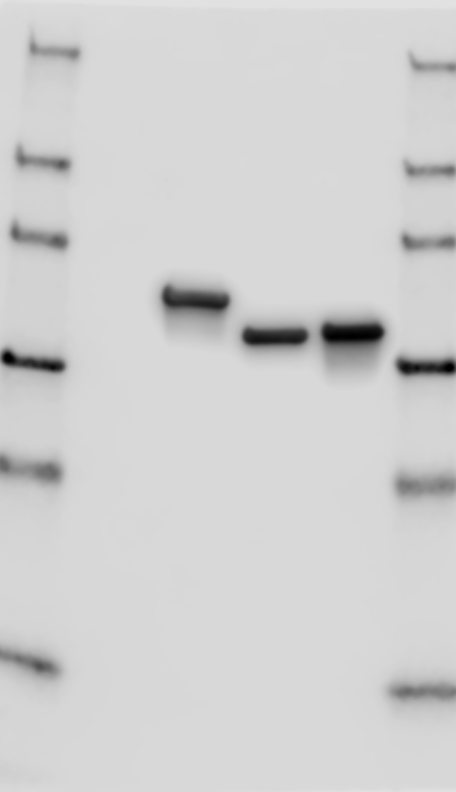

Supplement: Supplementary file 10 — Source data Fig. 7 [file 44319_2024_305_MOESM10_ESM.zip › Figure 7/7F IP FLAG-MPP7 mutants.tif]

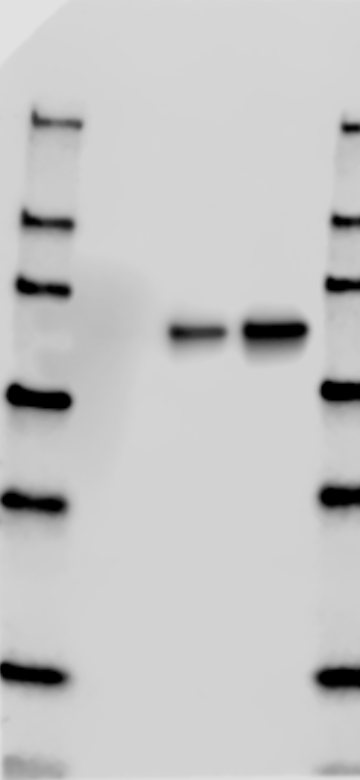

Supplement: Supplementary file 10 — Source data Fig. 7 [file 44319_2024_305_MOESM10_ESM.zip › Figure 7/7E co-IPed HA-YY1.tif]

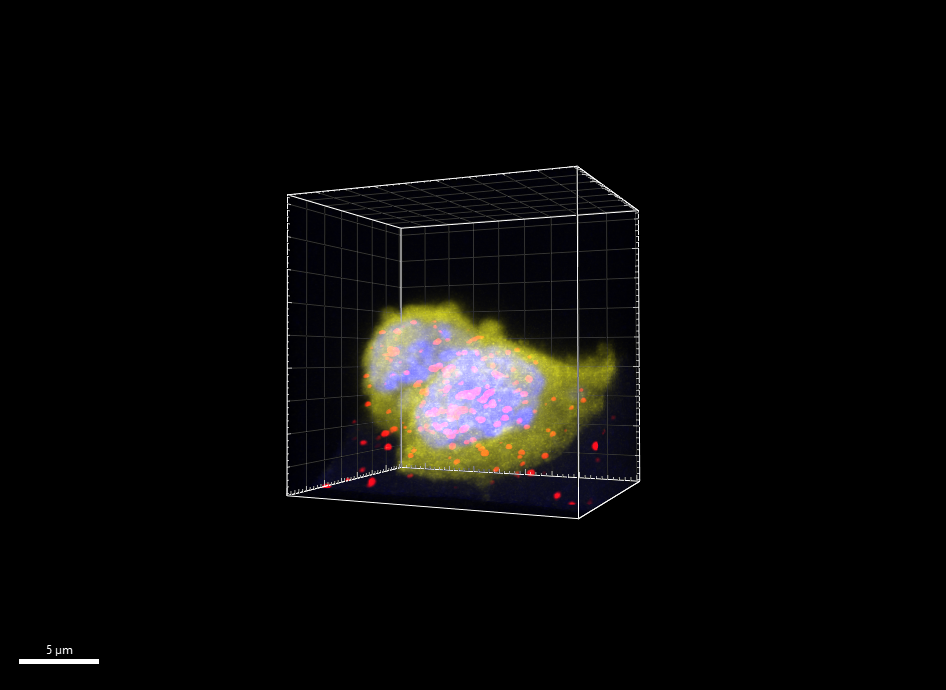

Supplement: Supplementary file 11 — Source data Fig. 8 [file 44319_2024_305_MOESM11_ESM.zip › Figure 8/Fig 8g Mpp7cKO+L27-TAZ PAX7-CARM1 GFP 3D PLA.png]

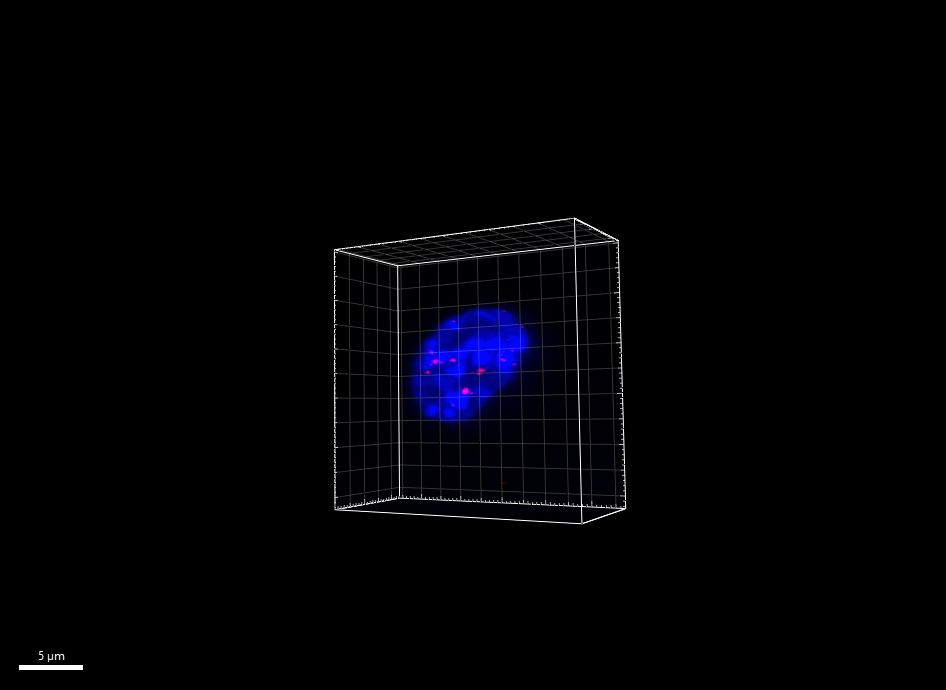

Supplement: Supplementary file 11 — Source data Fig. 8 [file 44319_2024_305_MOESM11_ESM.zip › Figure 8/Fig 8e Mpp7cKO YY1-YAPTAZ 3D PLA.png]

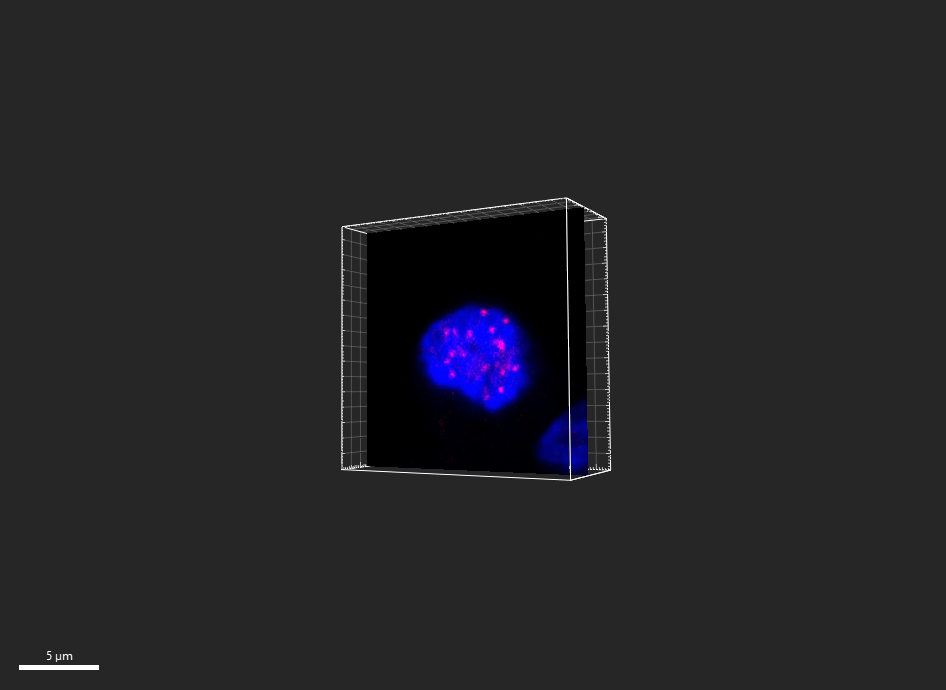

Supplement: Supplementary file 11 — Source data Fig. 8 [file 44319_2024_305_MOESM11_ESM.zip › Figure 8/Fig 8e Control YY1-YAPTAZ Transverse.png]

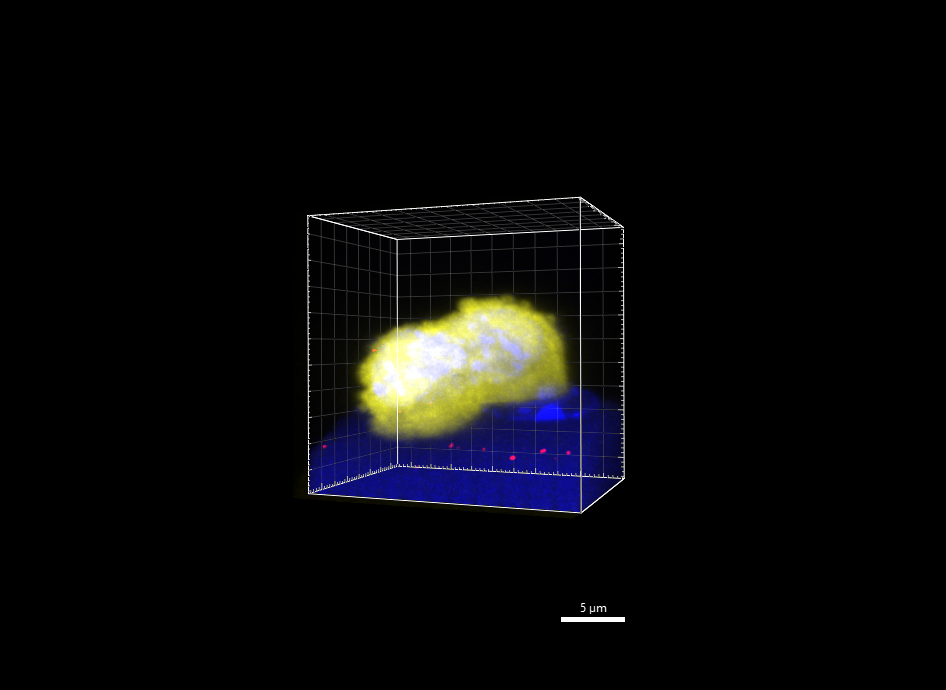

Supplement: Supplementary file 11 — Source data Fig. 8 [file 44319_2024_305_MOESM11_ESM.zip › Figure 8/Fig 8g Mpp7cKO+EV PAX7-CARM1 GFP 3D PLA.png]

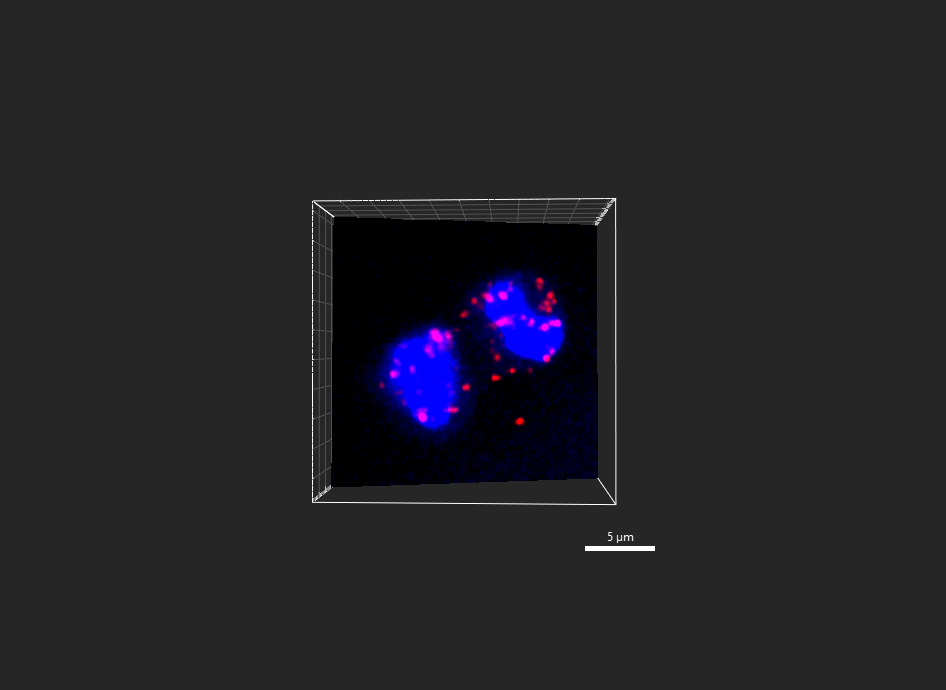

Supplement: Supplementary file 11 — Source data Fig. 8 [file 44319_2024_305_MOESM11_ESM.zip › Figure 8/Fig 8f YFP PAX7-CARM1 Transverse.png]

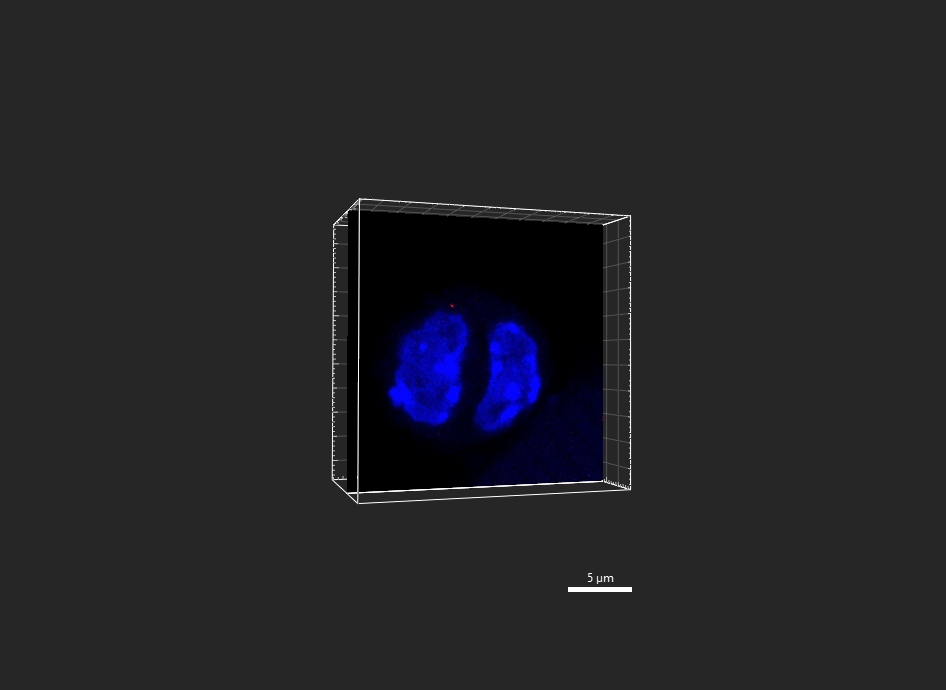

Supplement: Supplementary file 11 — Source data Fig. 8 [file 44319_2024_305_MOESM11_ESM.zip › Figure 8/Fig 8f Mpp7cKO PAX7-CARM1 Transverse.png]

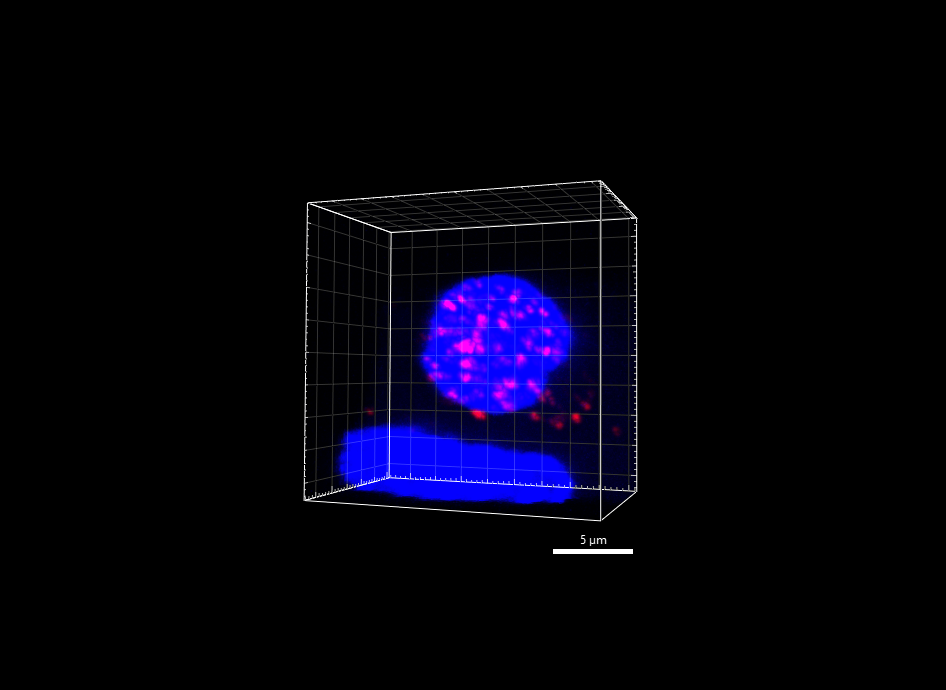

Supplement: Supplementary file 11 — Source data Fig. 8 [file 44319_2024_305_MOESM11_ESM.zip › Figure 8/Fig 8d MPP7-YY1 3D PLA.png]

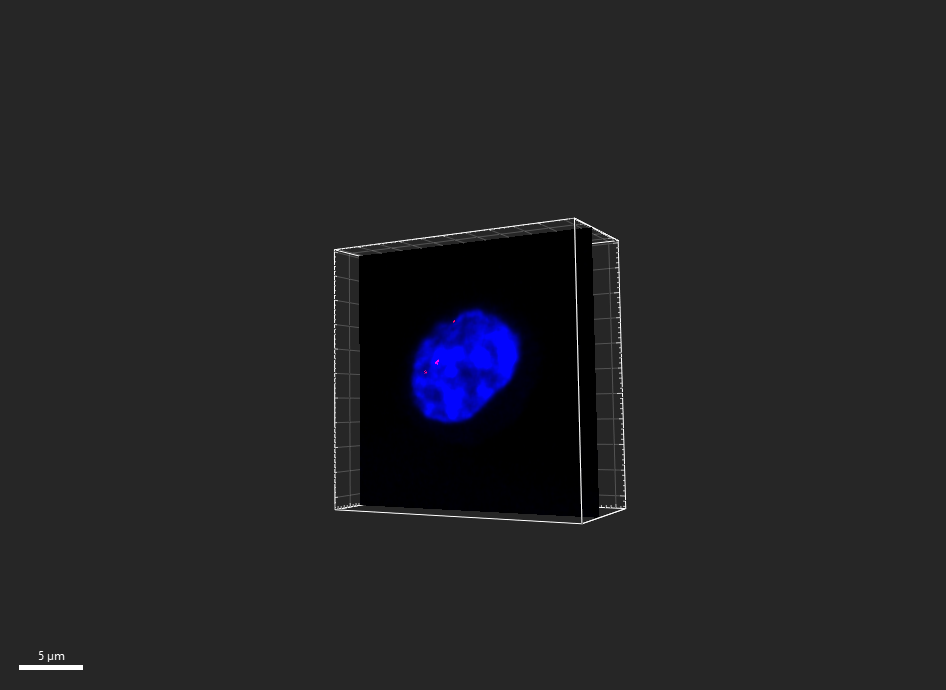

Supplement: Supplementary file 11 — Source data Fig. 8 [file 44319_2024_305_MOESM11_ESM.zip › Figure 8/Fig 8e Mpp7cKO YY1-YAPTAZ Transverse.png]

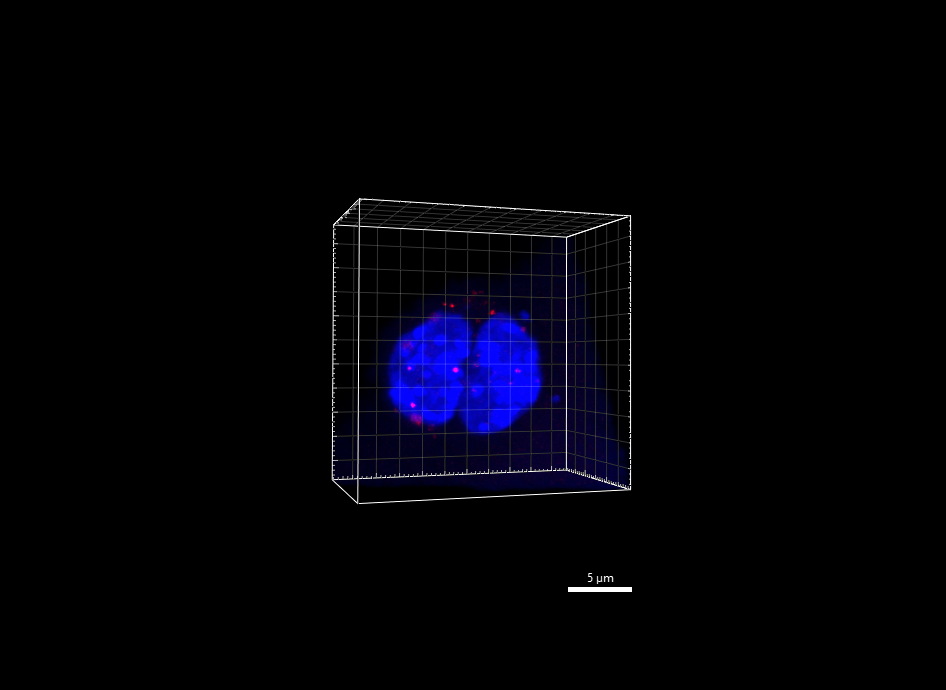

Supplement: Supplementary file 11 — Source data Fig. 8 [file 44319_2024_305_MOESM11_ESM.zip › Figure 8/Fig 8f Mpp7cKO PAX7-CARM1 3D PLA.png]

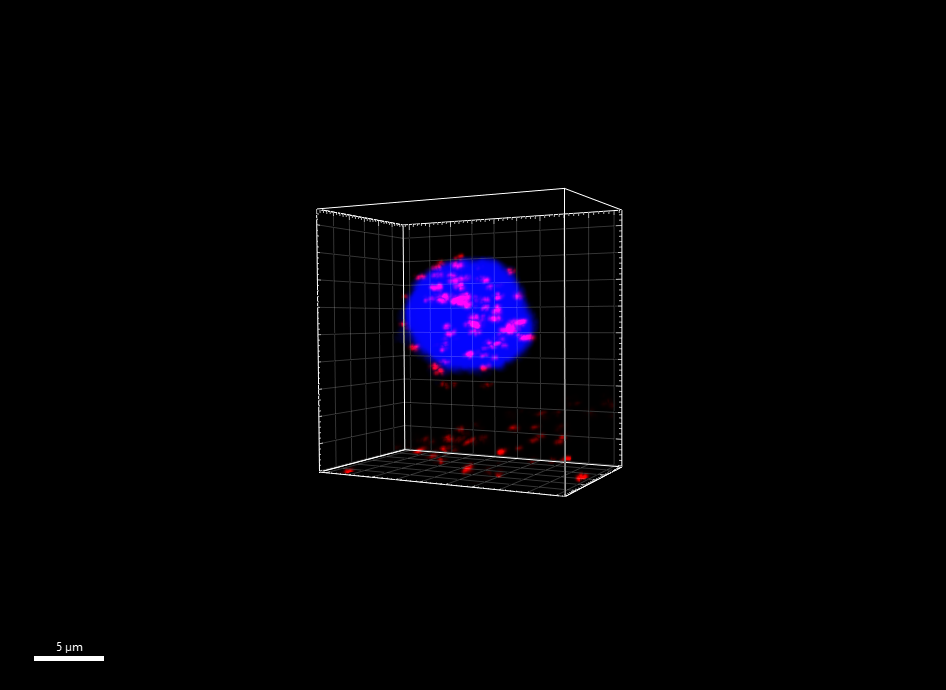

Supplement: Supplementary file 11 — Source data Fig. 8 [file 44319_2024_305_MOESM11_ESM.zip › Figure 8/Fig 8c MPP7-YAPTAZ 3D PLA.png]

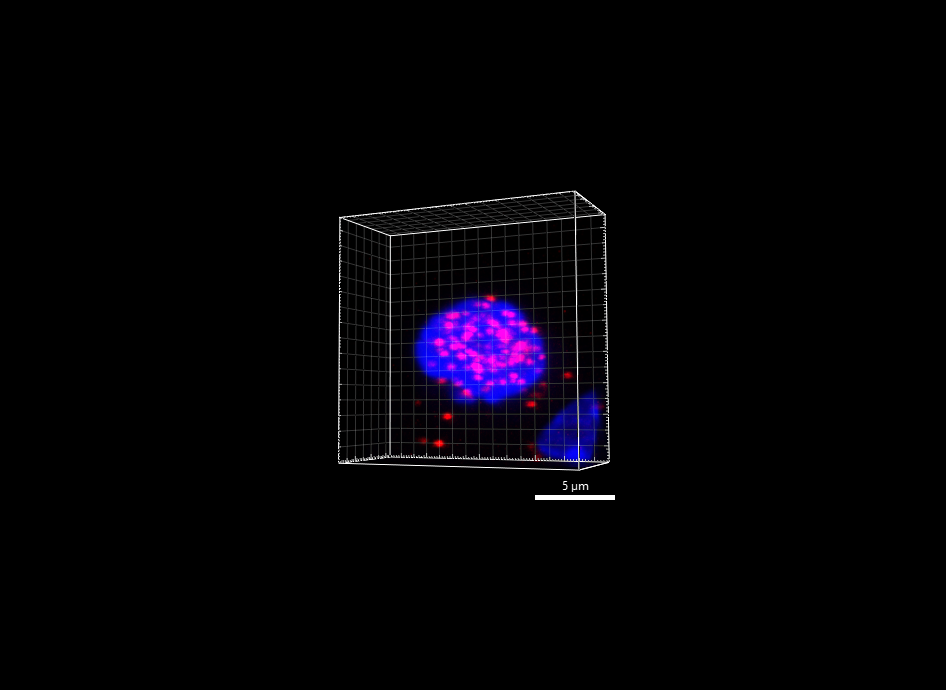

Supplement: Supplementary file 11 — Source data Fig. 8 [file 44319_2024_305_MOESM11_ESM.zip › Figure 8/Fig 8e Control YY1-YAPTAZ 3D PLA.png]

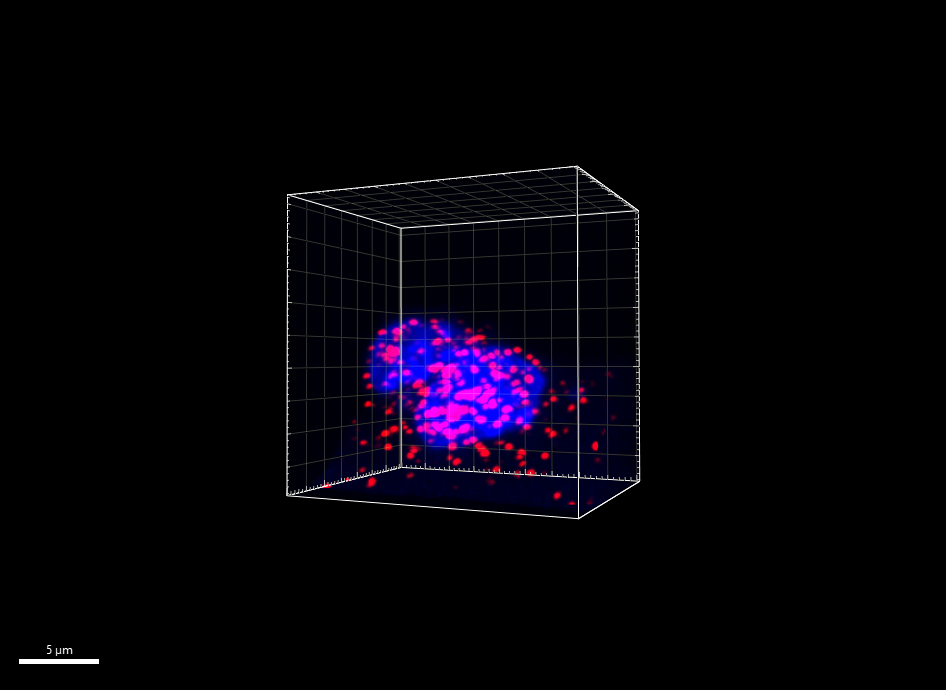

Supplement: Supplementary file 11 — Source data Fig. 8 [file 44319_2024_305_MOESM11_ESM.zip › Figure 8/Fig 8g Mpp7cKO+L27-TAZ PAX7-CARM1 3D PLA.png]
