# Supplementary figures and images for: The L27 domain of MPP7 enhances TAZ-YY1 cooperation to renew muscle stem cells (part 2 of 2)
Source: EMBO Rep. 2024 Nov 4;25(12):5667–86. doi: 10.1038/s44319-024-00305-4 (PMC11624273; doi:10.1038/s44319-024-00305-4)

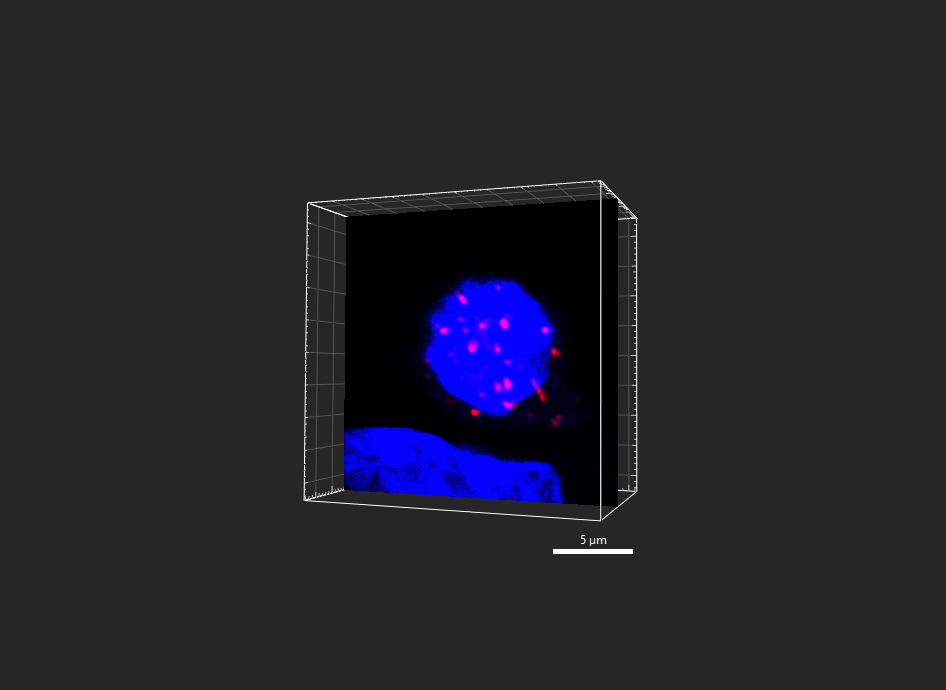

Supplement: Supplementary file 11 — Source data Fig. 8 [file 44319_2024_305_MOESM11_ESM.zip › Figure 8/Fig 8d MPP7-YY1 Transverse.png]

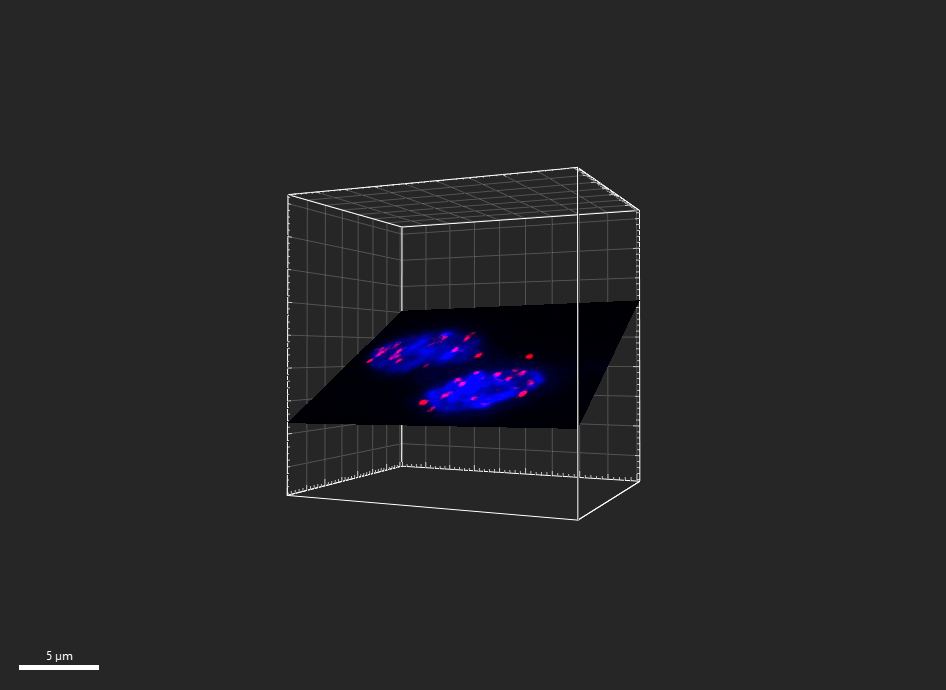

Supplement: Supplementary file 11 — Source data Fig. 8 [file 44319_2024_305_MOESM11_ESM.zip › Figure 8/Fig 8g Mpp7cKO+L27-TAZ PAX7-CARM1 Transverse.png]

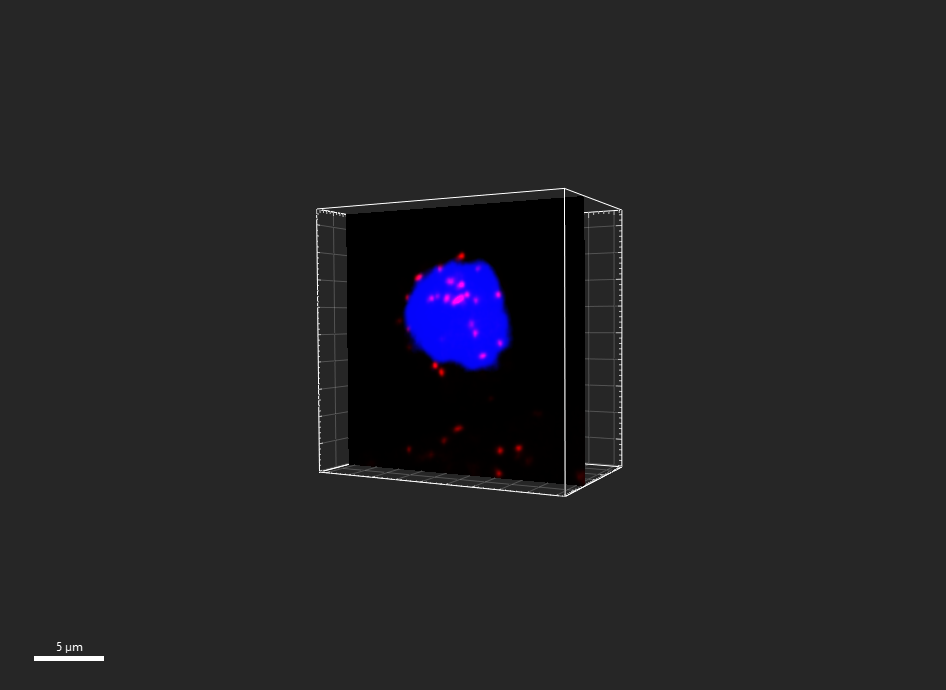

Supplement: Supplementary file 11 — Source data Fig. 8 [file 44319_2024_305_MOESM11_ESM.zip › Figure 8/Fig 8c MPP7-YAPTAZ Transverse.png]

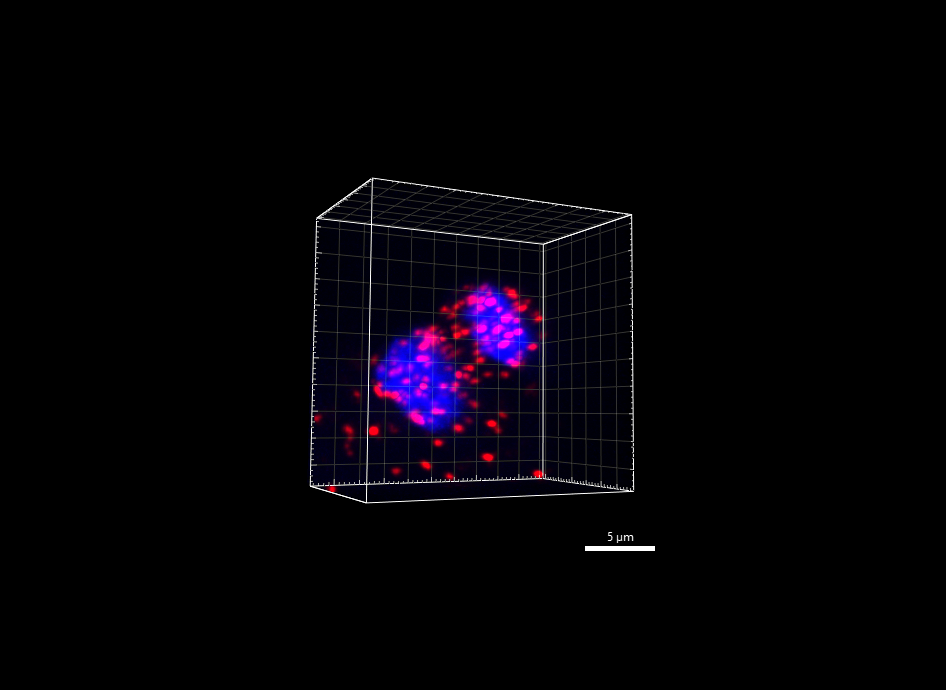

Supplement: Supplementary file 11 — Source data Fig. 8 [file 44319_2024_305_MOESM11_ESM.zip › Figure 8/Fig 8f YFP PAX7-CARM1 3D PLA.png]

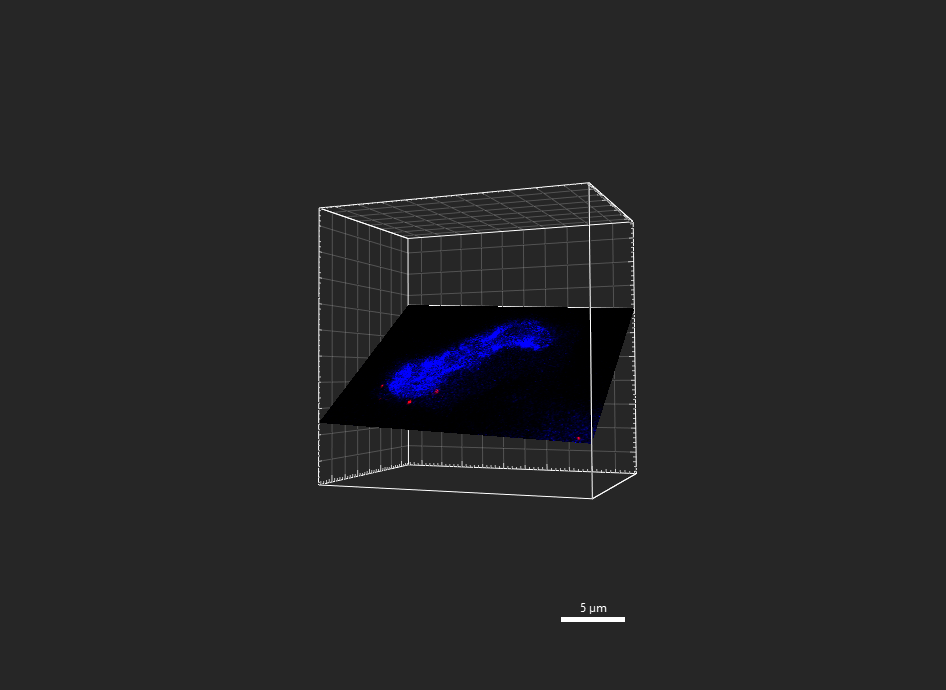

Supplement: Supplementary file 11 — Source data Fig. 8 [file 44319_2024_305_MOESM11_ESM.zip › Figure 8/Fig 8g Mpp7cKO+EV PAX7-CARM1 Transverse.png]

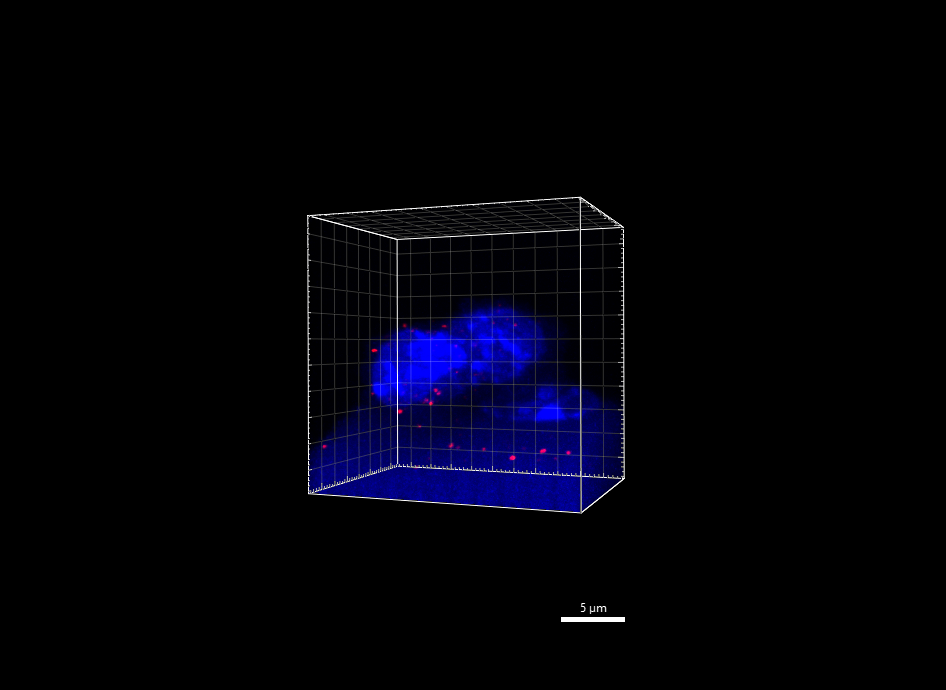

Supplement: Supplementary file 11 — Source data Fig. 8 [file 44319_2024_305_MOESM11_ESM.zip › Figure 8/Fig 8g Mpp7cKO+EV PAX7-CARM1 3D PLA.png]
